# Neuroimaging and neurophysiologic biomarkers for diagnosis and prognosis of depressive disorders, bipolar disorder, anxiety disorders, obsessive compulsive disorder, posttraumatic stress disorder, and substance use disorder: an evidence map

**DOI:** 10.1186/s12888-025-07429-4

**Published:** 2026-05-06

**Authors:** Catherine Sowerby, Adrienne Landsteiner, Kristen Ullman, Maylen Anthony, Caleb Kalinowski, Michele R. Spoont, Scott Sponheim, Kelvin Lim, Jose V. Pardo, Timothy J. Wilt, Wei Duan-Porter

**Affiliations:** 1 Minneapolis VA Health Care System, Center for Care Delivery and Outcomes Research, Minneapolis, MN USA; 2 University of Minnesota School of Medicine, Department of Medicine, Minneapolis, MN USA; 3 University of Minnesota School of Medicine, Department of Psychiatry and Behavioral Sciences, Minneapolis, MN USA; 4 Minneapolis VA Health Care System, Division of Psychology, Minneapolis, MN USA; 5 Minneapolis VA Health Care System, Geriatric Research Education and Clinical Center, Minneapolis, MN USA; 6 Minneapolis VA Health Care System, Cognitive Neuroimaging Unit, Minneapolis, MN USA; 7 University of Minnesota School of Public Health, Division of Health Policy and Management, Minneapolis, MN USA

**Keywords:** Mental health, Medical neurobiology, Psychiatry, Neuroscience, Medical sciences

## Abstract

**Background:**

Advancements in precision medicine, particularly the use of neuroimaging and neurophysiologic techniques, may improve diagnosis, prognosis, and treatment of mental health disorders. Recent efforts to develop large neuroimaging datasets have yielded promising results for identifying mental health biomarkers. This scoping review identifies and characterizes studies of neuroimaging and neurophysiologic techniques used to address a variety of mental health disorders.

**Methods:**

We searched MEDLINE and Embase (January 2010-September 2023). Eligible studies examined neuroimaging and neurophysiologic techniques (e.g., magnetic resonance imaging [MRI] or electroencephalogram [EEG]) for diagnosis, prognosis, and/or treatment response for eligible mental health disorders. From eligible studies, we abstracted information on populations, clinical settings, imaging techniques, study designs, outcomes, and analytic approaches.

**Results:**

From 58,824 unique search results, we identified 441 eligible primary studies and 27 systematic reviews addressing mental health disorders. Most studies focused on depressive disorders (*k* = 320 primary studies [17 systematic reviews]); fewer examined bipolar disorders (*k* = 61 [3]), posttraumatic stress disorder (PTSD; *k* = 39 [2]), obsessive compulsive disorder (OCD; *k* = 26 [1]), anxiety disorders (*k* = 22 [3]), or substance use disorders (SUD; *k* = 25 [0]). Three-quarters of primary studies used MRI-based techniques and 20% employed EEG. Two-thirds of studies focused on diagnosis (nearly all cross-sectional); the remaining studies mostly addressed symptom response to various treatments, including antidepressants and psychotherapy. Most primary studies were small (*N* < 100; *k* = 263), and generally included y oung and middle-aged adults; only 5 focused on older adults (sample mean age ≥ 65). Studies were most commonly conducted in China (*k* = 181), the United States (*k* = 83), or Canada (*k* = 22).

**Conclusions:**

Although many eligible studies evaluated MRI or EEG for diagnosis and/or treatment response for depressive disorders, most were small and cross-sectional. There was less existing evidence examining other neuroimaging techniques or focusing on other mental health disorders (PTSD, OCD, anxiety disorders, or SUD). Given these evidence gaps, it is likely premature to implement neuroimaging and neurophysiologic tests in clinical settings. To determine clinical utility, future research should use large samples in longitudinal designs and investigate a broader set of disorders.

**Trial registration:**

10.17605/OSF.IO/5PHG2. Clinical trial number: not applicable.

**Supplementary Information:**

The online version contains supplementary material available at 10.1186/s12888-025-07429-4.

## Background

In the past several decades, there have been significant advancements in precision medicine, specifically the use of biomarkers in diagnosis, prognosis, and treatment of medical conditions. Precision medicine aims to personalize care based on more detailed knowledge of individual variations in disease mechanism and prognosis [1]. For treatment of mental health disorders, precision medicine has primarily focused on identifying drug metabolic pathways and other genomic factors in treatment response [2]. Other approaches use neuroimaging techniques and neuropsychological measurements to assess brain structure, function, and activity [3].

Recent efforts to systematically collect and examine large neuroimaging datasets have yielded promising results for identifying biomarkers for individuals with psychiatric disorders. Several ongoing large-scale, population-based studies also aim to advance precision medicine, including the United Kingdom (UK) Biobank database [4, 5]. Despite advances in the development and availability of neuroimaging and neurophysiologic assessment techniques, substantial challenges exist in application to clinical practice [2, 3, 6]. Larger studies and newer paradigms are likely needed for translation into clinical settings, particularly due to clinical heterogeneity and symptom overlap among psychiatric disorders [6].

In response to congressional mandates to advance and, if appropriate, implement precision medicine for mental health treatment of United States (US) veterans, the Department of Veterans Affairs (VA) convened a workgroup to identify targets for future research and clinical demonstration projects. As a critical first step to understanding the state of research on using neuroimaging and neurophysiologic biomarkers to support diagnosis and/or treatment for psychiatric disorders, the VA Evidence Synthesis Program (ESP) conducted an evidence map. An evidence map is well suited to characterize the current evidence base, particularly when it may consist largely of exploratory studies [7, 8]. An evidence map is also the most appropriate method for meeting the broad scope of assessing the state of the science across numerous techniques and disorders [7, 8].

To facilitate future research and investment in precision medicine approaches to mental health diagnosis and treatment, we summarize the main results of the VA ESP evidence map evaluating the use of neuroimaging and neurophysiologic biomarkers in the diagnosis and treatment of depressive disorders, bipolar disorders, anxiety disorders, obsessive-compulsive disorder (OCD), posttraumatic stress disorder (PTSD), and substance use disorders (SUD) [9]. We provide descriptive information about the existing studies and highlight weaknesses and gaps in the evidence, as determined by the volume and characteristics of studies.

## Methods

The protocol (developed *a priori)* is registered with Open Science Framework (S1 File; 10.17605/OSF.IO/5PHG2). Clinical trial number: not applicable.

### Scope and key question

The main goals of the VA ESP report were to guide research policy and potential clinical demonstration projects of neuroimaging and neurophysiologic tests for a variety of mental health disorders and traumatic brain injury (TBI) [9]. As an initial step towards understanding the evidence base for this broad scope, we conducted an evidence map to describe the characteristics of the existing published literature. In this paper, we focus on results addressing diagnosis and prognosis of mental health disorders:

Key Question: *What are the quantity*,* distribution*,* and characteristics of evidence assessing the accuracy and utility of neuroimaging and neurophysiologic biomarkers in the diagnosis and clinical management of the following disorders: depressive disorders*,* bipolar disorder*,* anxiety disorders*,* OCD*,* PTSD*,* and SUD?*

### Data sources and searches

We searched MEDLINE and Embase from January 2010 to September 2023 (search strategy in S2 File). We elected to search for articles published after 2010 because a key goal of this review was to understand whether there were more recent advances within the evidence base on neuroimaging and neurophysiologic techniques for psychiatric disorders. We also searched websites for VA ESP and Agency for Healthcare Research and Quality Evidence-based Practice Centers to identify relevant reviews.

### Study selection

Eligible English-language studies and reviews included adults with at least one disorder of interest, employed at least one test of interest (e.g., magnetic resonance imaging [MRI], including functional MRI [fMRI], and evoked potentials and electroencephalogram [EEG]), and assessed diagnostic accuracy compared with gold standard assessments, clinical prognosis, and/or treatment response. Exclusion criteria were pediatric populations, mental health symptoms or cognitive functioning only in neurodegenerative disorders or intracranial injury, and exclusive use of cross-sectional data for prognosis or treatment response. Detailed eligibility criteria are provided in Table 1.

Abstracts were uploaded in DistillerSR [10] and screened with the assistance of machine-learning tools (DistillerSR Artificial Intelligence System [DAISY]) in three separate phases involving a variable number of human reviewers. In the first phase, two human reviewers were required to agree on exclusion (abstracts could be included by single human reviewer); approximately 12,000 abstracts were reviewed, and this phase also served as training for DAISY. The second phase occurred when the rate of inclusion had fallen below 5% and DAISY predicted likelihood of inclusion was ≤ 0.4 for all remaining articles. During this phase, a single human reviewer screened about 7,000 abstracts, with DAISY serving as the second reviewer; DAISY predicted likelihood of inclusion was 0.2–0.3 for these articles. Finally, no human reviewer screened the remaining abstracts with DAISY predicted likelihood < 0.2 (25,912 abstracts); before this point, the inclusion rate had been 0.066% for the last 1,526 abstracts screened by a human reviewer. We conservatively estimate that an additional 17 abstracts may have been missed for Full-text review, due to stopping of single human review of abstracts in this last phase. Full-text review was primarily conducted by a single human reviewer and DAISY acting as the second reviewer, after two initial pilot rounds to ensure common understanding and operationalization of eligibility criteria. Approximately 50% of articles included at full-text review were also evaluated again by a second human reviewer. The use of machine-learning tools in screening and selection of studies has been documented to have good sensitivity, while enabling more efficient processing of a large amount of published evidence [11–13].

**Table 1:** Eligibility criteria for selection of studies

	Inclusion criteria	Exclusion criteria
Population	*N* ≥ 30, ≥ 18 years of age or older, with the following conditions: • Depression • Anxiety (including OCD, phobias and panic disorders) • Posttraumatic stress disorder (PTSD) • Substance use disorder (SUD) • Bipolar disorder • Traumatic brain injury (TBI)	• Pediatric populations or mixed pediatric and adult populations without stratified data • Non-human studies • Stroke patients (CVA) • Multiple sclerosis • Intracranial hemorrhage (intracranial etiology, e.g., burst aneurysm) • Neurodegenerative conditions (e.g., dementia, Parkinson’s disease) except if assessed as outcomes or subsets of eligible conditions
Neuroimaging or Neurophysiologic Technique	• Magnetic resonance imaging (MRI) • Functional magnetic resonance imaging (fMRI) • Diffusion tensor imaging (DTI) • Perfusion weighted imaging (PWI) • Magnetic resonance spectroscopy (MRS) • Positron emission tomography (PET) • Single photon emission computed tomography (SPECT) • Arterial spin labeling (ASL) • Magnetoencephalography (MEG) • Evoked potentials and electroencephalogram (EEG) • Paired pulse transcranial magnetic stimulation (ppTMS)	NA
Outcomes	Diagnostic accuracy compared with: • Validated structured clinical interviews (e.g., MINI, SCID-5, WHO WMH-CIDI) • Validated clinician reported instruments of symptoms (e.g., CAPS, HDRS, HAM-A) • Patient-reported measures of mental health symptoms (e.g., PCL-5, PHQ-9, HADS, BDI, GAD-7, AUDIT) • Measures of cognition, other psychiatric symptoms (e.g., delusions, hallucinations) Prognosis and treatment response: • Change in symptoms, cognition, functioning (e.g., SF-36, WHODAS) • Sobriety/abstinence or reduction in substance use (SUD only) • Recurrence or relapse (study must define criteria and use validated measures) • Sensitivity (vs. lack of response) to treatment • Self-harm behaviors or suicide risk • Adverse events and side effects	Only between-group differences (e.g., in neuroimaging findings) or cross-sectional correlations with symptom severity
Timing	Published 2010 or later	Earlier than 2010
Setting	Any	
Study design	Observational studies, trials, and systematic reviews	Study protocols, case reports, abstracts, editorials; for prognosis and treatment response outcomes, cross-sectional studies

*Abbreviations*. *ASL* Arterial spin labeling, *AUDIT* Alcohol screening questionnaire, *BDI* Beck Depression Inventory, *CAPS* Clinically Administered Posttraumatic Stress Disorder Scale, *CVA* Cerebrovascular accident, *DTI* Diffusion tensor imaging, *EEG* Evoked potentials and electroencephalogram, *fMRI* Functional magnetic resonance imaging, *GAD-7* General Anxiety Disorder-7, *HADS* Hospital Anxiety and Depression Scale, *HAM-A* Hamilton Anxiety Rating Scale, *HDRS* Hamilton Depression Rating Scale, *MEG* Magnetoencephalography, *MINI* Mini International Neuropsychiatric Interview, *MRI* Magnetic resonance imaging, *MRS* Magnetic resonance spectroscopy, *OCD* Obsessive compulsive disorder, *PCL-5* Posttraumatic Stress Disorder Checklist for Diagnostic and Statistical Manual of Mental Disorders, 5th Edition, *PET* Positron emission tomography, *PHQ-9* Patient Health Questionnaire-9, *ppTMS* Paired pulse transcranial magnetic stimulation, *PTSD* Posttraumatic Stress Disorder, *PWI* Perfusion weighted imaging, *SCID-5* Structured Clinical Interview for the Diagnostic and Statistical Manual of Mental Disorders, 5th Edition, *SF-36* 36-Item Short Form Survey, *SPECT* Single photon emission computed tomography, *SUD* Substance use disorder, *TBI* Traumatic brain injury, *WHO* World Health Organization, *WHODAS* World Health Organization Disability Assessment Schedule, *WMH-CIDI* World Mental Health Composite International Diagnostic Interview

### Data abstraction

We abstracted the following data: population characteristics; neuroimaging tests and/or EEG; clinical setting; comparator for diagnostic accuracy, diagnostic, prognostic, and/or treatment response outcomes; study design; and analytic methods (e.g., statistical models and diagnostic standards). Data from ~ 50% of articles were verified by a second reviewer.

### Quality assessment and summary of results

As appropriate for evidence maps, we did not conduct formal quality or risk of bias assessments of individual studies. We present results organized by conditions of interest, focusing on characteristics of study populations, outcomes, and study designs (including analytic methods). We also note methodological limitations and evidence gaps.

## Results

### Overview

**Fig. 1:**
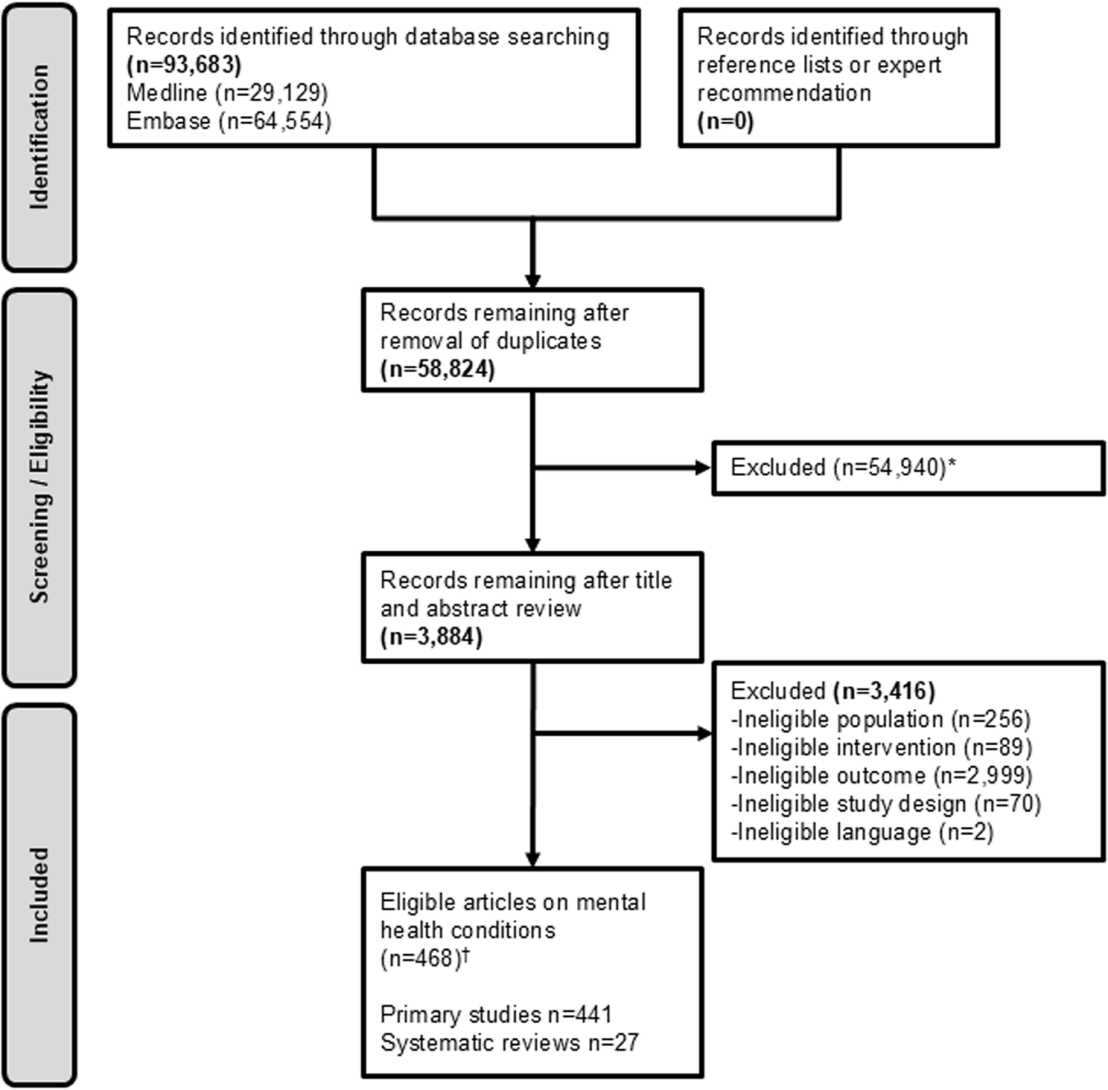
Search & selection of eligible studies. *n=27,915 excluded with probability score <0.2 by machine-learning algorithm DAISY on the DistillerSR platform (Evidence Partners). †There were 9 other eligible articles on traumatic brain injury that were included in the original VA ESP report [9]

From 58,824 unique search results, we identified 441 eligible primary studies and 27 systematic reviews on mental health conditions (Fig. 1). Most articles focused on depressive disorders (*k=*320primary studies and 17 systematic reviews [14–30]). Fewer primary studies addressed bipolar disorder, anxiety disorders, OCD, PTSD, or SUD. Systematic reviews also addressed anxiety disorders (*k=*3) [31–33], bipolar disorders (*k=*4) [34–37], PTSD (*k=*2) [38, 39], or OCD (*k=*1) [40] (S4 File). Most systematic reviews employed MRI-based techniques (*k=*16) [15, 17, 18, 23–26, 28, 31, 33, 35–37, 39, 41, 42] or EEG (*k=*5) [14, 21, 27, 29, 30], or included multiple types of neuroimaging data (*k=*7) [19, 20, 22, 32, 34, 38, 40].

**Fig. 2:**
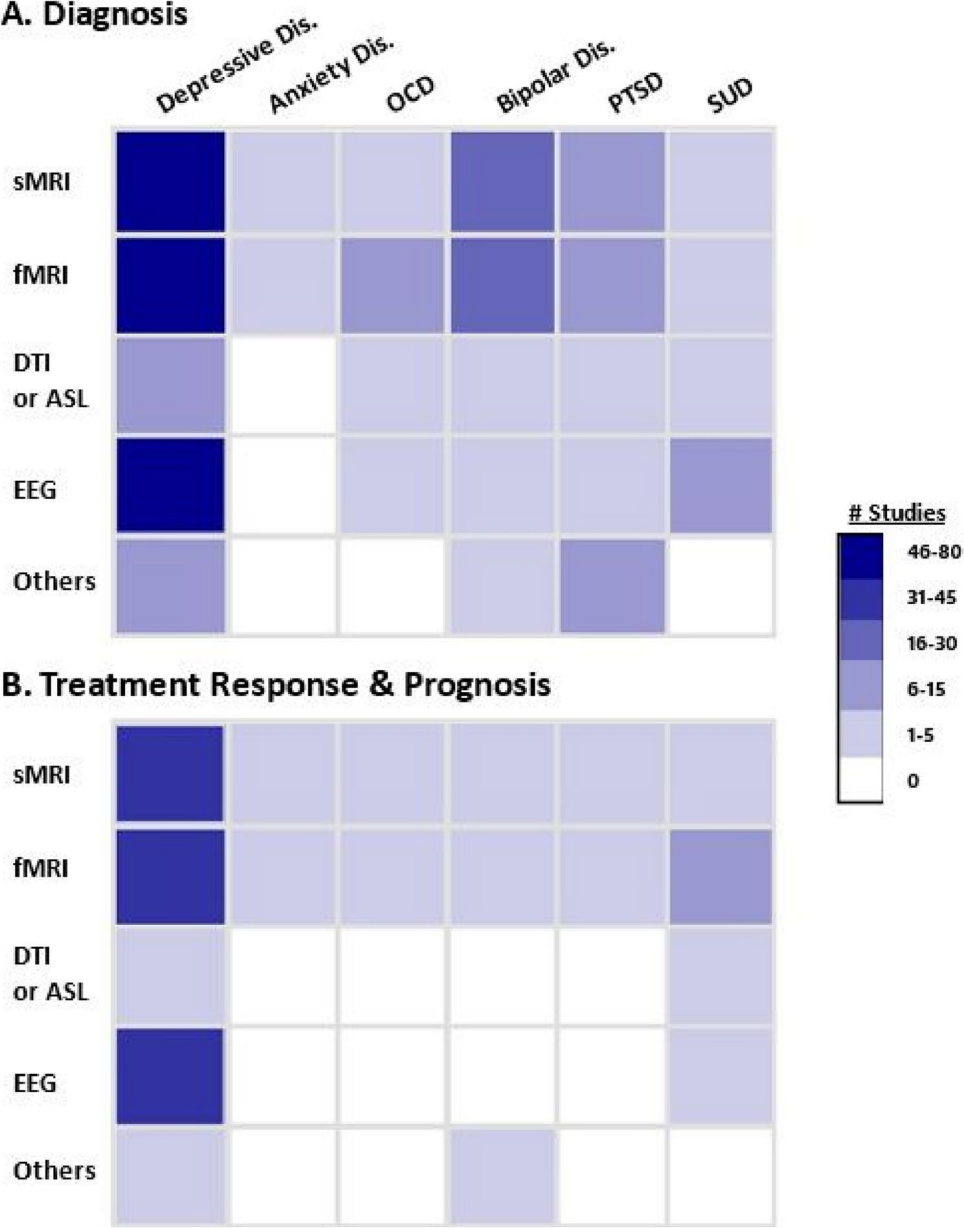
Number of primary studies using neuroimaging or neurophysiologic data to evaluate (**A**) diagnosis or (**B**) treatment response and prognosis for various mental health disorders. *Abbreviations*. ASL= arterial spin labeling; dis=disorder; DTI=diffusion tensor imaging; EEG=electroencephalogram; fMRI=functional magnetic resonance imaging; MRI=magnetic resonance imaging; OCD=obsessive compulsive disorder; PTSD=posttraumatic stress disorder; sMRI=structural magnetic resonance imaging; SUD=substance use disorder. Notes. “Others” category includes positron emission tomography (PET), single-photon emission computerized tomography (SPECT), and magnetoencephalography (MEG)

Figure 2 depicts the distribution of primary studies and Table 2 summarizes the population characteristics and methods. Citations and detailed characteristics are provided in S5 File. Below, we first describe primary studies focusing on depressive disorders, and then more briefly present evidence on other mental health disorders.

**Table 2:** Summary characteristics of primary studies

Study characteristics	Depressive disorders (k = 320)	Anxiety disorders^a^ (k = 22)	OCD (k = 26)	Bipolar disorders (k = 61)	PTSD (k = 39)	SUD (k = 25)
*Imaging technique*						
MRI-based techniques:						
sMRI	94	7	8	30	9	6
fMRI	118	11	16	28	15	11
DTI or ASL	14	―	1	5	1	2
EEG	100	4	2	1	11	9
Others^b^	18	―	―	4	6	―
*Outcomes*						
Diagnosis	201	16	22	53	31	14
Prognosis	9	1	―	1	1	9
Treatment response^c^	119	7	4	7	7	2
*Study design & methods*						
Cross-sectional	178	13	17	49	25	11
Cohort/longitudinal observational	126	9	9	12	12	13
Randomized controlled trial (RCT)	16	―	―	―	2	1
Used machine learning	181	12	10	38	18	7
Models validated	216	15	15	36	18	12
*Country*						
US/Canada	68	7	1	14	20	12
China	131	10	20	23	8	4
UK/Europe	44	2	1	12	2	5
Others^d^	72	3	3	11	6	3
NR	5	―	1	1	3	1
*Sample sizes (total N)* ^*e*^						
30–99	187	17	14	28	21	17
100–200	82	5	10	25	10	6
201–500	33	―	2	6	6	1
501–1000	5	―	―	―	―	―
> 1,000	9	―	―	1	1	1
NR	4	―	―	1	1	―
*Age (mean or median*,* years)*						
18–25	17	7	4	8	2	―
26–44	231	14	14	44	26	15
45–64	35	―	―	4	5	6
> 65	6	―	―	1	―	―
NR	28	5	1	2	6	4
*% Women*						
0–15	2	―	―	―	11	10
16–40	16	6	10	4	5	10
41–70	226	17	8	48	14	2
> 70	36	2	―	4	5	―
Not reported	38	3	1	4	4	3
*Other groups included*						
Medication free	94	13	12	4	8	4
First episode	24	―	―	2	―	―
Treatment resistant	30	―	2	5	―	―
Healthy controls	219	14	21	43	27	14

*Abbreviations*. *ASL* Arterial spin labeling, *DTI* Diffusion tensor imaging, *EEG* Electroencephalogram, *MRI* Magnetic resonance imaging, *OCD* Obsessive compulsive disorder, *PTSD* Posttraumatic stress disorder, *RCT* Randomized controlled trial, *SUD* Substance use disorder, *UK* United Kingdom, *US* United States

^a^Includes general anxiety disorder, panic disorder, and social anxiety disorder

^b^Includes positron emission tomography (PET), single-photon emission computerized tomography (SPECT), and magnetoencephalography (MEG)

^c^For SUD, this was abstinence vs. relapse after or during treatment

^d^Includes other countries not included in categories above, as well as studies done in multiple countries

^e^Also includes healthy controls if among participants

### Depressive disorders

**Fig. 3:**
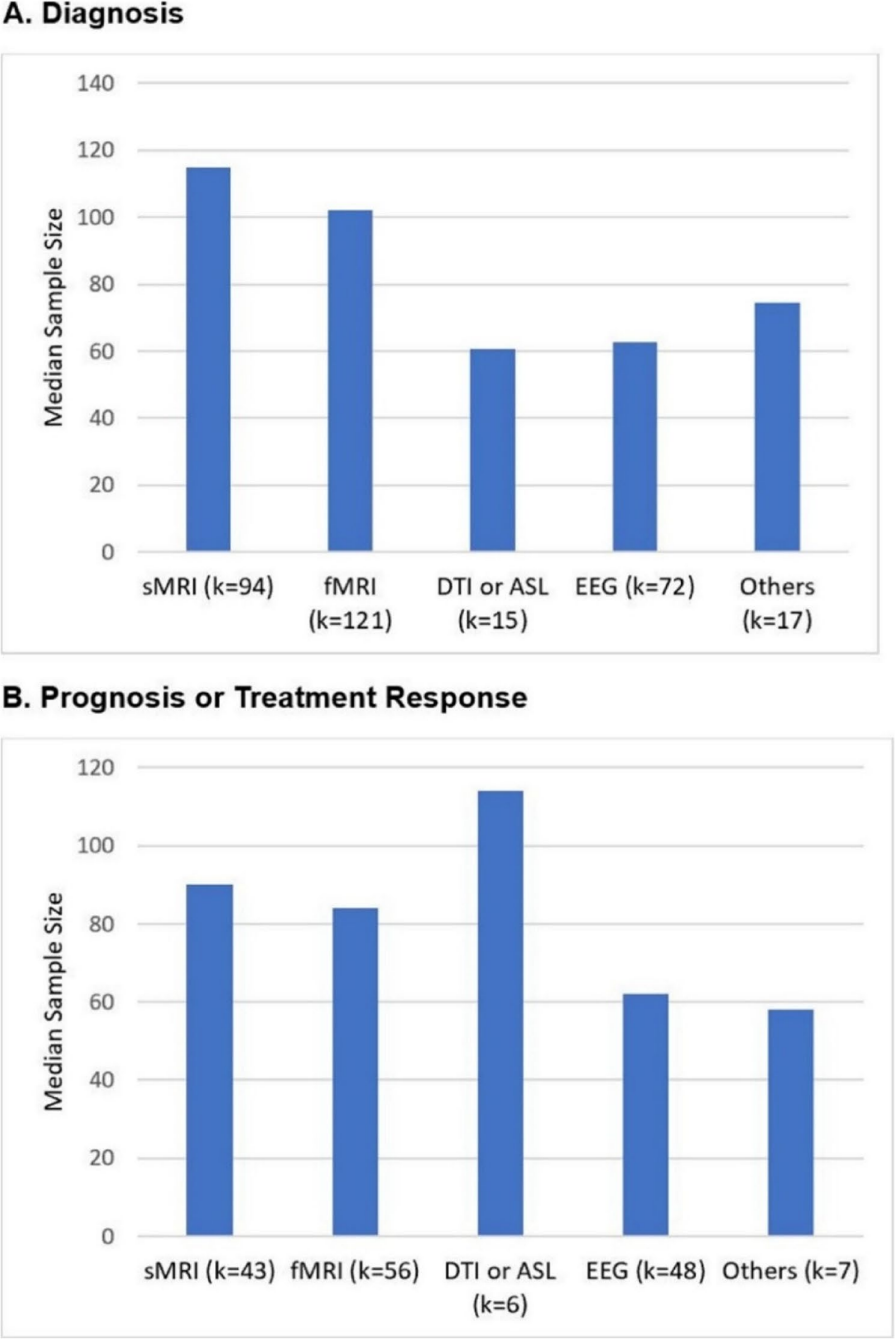
Median sample size of included studies evaluating (**A**) diagnosis or (**B**) treatment response or prognosis for depressive disorders. *Abbreviations*. ASL = arterial spin labeling; DTI = diffusion tensor imaging; EEG = electroencephalogram; fMRI = functional magentic resonance imaging; sMRI = structural magnetic resonance imaging. *Notes*. “Others” category includes positron emission tomography (PET), single-photon emission computerized tomography (SPECT), and magnetoencephalography (MEG). Number of included studies is indicated for each type of imaging or neurophysiologic data

 Two thirds of the primary studies focusing on depressive disorders employed structural and/or functional MRI (*k*=212) (Table 2). Remaining studies used EEG (*k*=100) or other neuroimaging techniques. Diagnostic accuracy was the most common outcome assessed (*k*=201, 63%) and most studies were small with total *N*<100 (*k*=187, 58%). Only nine studies had total *N*>1000 (see Figure 3). Study populations consisted of predominantly young and middle-aged adults, with substantial proportions of women. Studies were conducted in various countries, most commonly in China (*k*=131).

#### Primary studies evaluating diagnosis

Table 3 summarizes characteristics of 201 studies evaluating diagnosis of depressive disorders. Of 142 studies using MRI-based techniques, the largest proportion used structural MRI (k=64), fMRI (k=78; 67 resting and 11 task-specific), or both (k=5). A few used other MRI-based techniques like DTI (k=8) and ASL (k=2). Most MRI studies were cross-sectional (k=133). More than half the studies had N<100 (k=122) and only 11 had N>1000. Most included healthy controls (k=193), while a fifth also included participants with bipolar disorder (k=32). A quarter of studies focused on medication-free participants (k=54), variably defined as those who were medication naive or those not on medications at the time of the study. Few included participants during their first episode of depression (k=22). The most common diagnostic standards were clinician assessments (eg, Hamilton Depression Rating Scale [HAM-D; k=112]) or structured interviews (eg, Structured Clinical Interview for DSM [SCID; k=111] and Mini-International Neuropsychiatric Interview [MINI; k=22]). Fewer studies used patient-reported measures such as the Beck Depression Inventory (BDI; k=25).

**Table 3:** Summary characteristics of primary studies evaluating diagnosis of depressive disorders

Study characteristics	sMRI (k = 64)	fMRI (k = 78)	ASL or DTI (k = 10)	EEG (k = 55)	Other techniques^a^ (k = 11)
*Depression subgroups*					
Medication free	16	27	2	5	4
First episode	7	12	--	2	1
Treatment resistant	1	3	--	―	―
*Other groups included*					
Healthy controls	57	71	8	47	10
Bipolar disorder patients	16	10	3	1	2
*Country*					
US/Canada	9	7	4	6	3
China	35	51	5	20	6
UK/Europe	10	9	1	9	―
Other^b^	9	10	―	18	2
Not reported	1	1	―	2	―
*Sample sizes (total N)*					
30–99	26	36	7	46	7
100–249	26	23	1	6	2
250–499	6	8	1	3	―
500–999	1	5	―	―	―
> 1,000	4	5	1	―	1
Not reported	1	1	―	―	1
*Diagnostic accuracy standard*					
Clinician interviews	61	67	9	35	11
Clinician assessments	58	75	8	36	8
Patient-reported outcomes	14	31	1	41	3
*Study design*					
Cross-sectional	55	69	9	49	10
Cohort/longitudinal	9	9	1	5	1
RCT	―	―	―	1	―
*Analytic methods*					
Sensitivity/specificity	55	69	9	48	8
Machine learning	42	58	7	34	4
Models validated	46	60	9	42	8

*Abbreviations*. *ASL * Arterial spin labeling, *DTI* Diffusion tensor imaging, *EEG* Electroencephalogram, *fMRI* Functional magnetic resonance imaging, *RCT* Randomized controlled trial, *sMRI* Structural magnetic resonance imaging (structural or functional), *UK* United Kingdom, *US* United States

^a^Includes positron emission tomography (PET), single-photon emission computerized tomography (SPECT), and magnetoencephalography (MEG)

^b^Includes other countries not included in categories above, as well as studies done in multiple countries

Fifty-five studies employed EEG and nearly all were cross-sectional. Most were also very small, with only nine having N≥100 (range 30–400). All but eight included healthy controls (k=47); only two studies focused on participants in their first episode of depression. Clinician assessments (HAM-D and Montgomery-Asberg Depression Rating Scale [MADRS]) were the most frequently used diagnostic standard (k=28).

Eleven studies used other neuroimaging techniques, including magnetoencephalography (MEG; k=7), positron emission tomography (PET; k=2), and single-photon emission computerized tomography (SPECT; k=1). All but one were cross-sectional and most were small (N=41-108), with the single exception of the SPECT study (N=4,541). MEG studies were conducted in China or Taiwan, while PET and SPECT studies occurred in the US. MEG and PET studies included healthy controls, while the SPECT study compared data of individuals with cognitive disorders, depression, or both. Four MEG studies focused on medication-free patients, all of which used clinician assessments (HAM-D) or structured interviews (SCID or MINI) as the diagnostic standard.

#### Primary studies evaluating treatment response & prognosis

Eighty-nine studies evaluated association of neuroimaging or neurophysiologic changes with treatment response and most of these used MRI-based techniques (k=57; see Table 4 for summary characteristics of studies evaluating treatment response). Twenty studies used structural MRI, 31 used fMRI (15 resting, 14 task-specific, and two both resting and task), two used both, and five studies used DTI. Most studies evaluated response to antidepressants (k=58), while others examined psychotherapy, electroconvulsive therapy (ECT), repetitive transcranial magnetic stimulation (rTMS), transcranial direct current stimulation (tDCS), theta burst stimulation (TBS), and inpatient multi-modal treatment.

**Table 4:** Summary of characteristics of included studies evaluating treatment response in depressive disorders

Study characteristics	Response to treatments				
	Antidepressants (k = 71)	Psychotherapies (k = 5)	ECT (k = 10)	rTMS (k = 25)	Other^a^ (k = 10)
*Imaging technique*					
sMRI	21	―	8	1	1
fMRI	23	4	3	6	5
DTI	4	―	―	―	1
EEG	23	1	―	17	4
Others^b^	5	―	―	2	―
*Depression subgroups*					
Medication free	36	2	―	4	3
Treatment resistant	5	―	7	14	1
*Other groups included*					
Healthy controls	33	3	2	5	1
Bipolar disorder	―	―	4	2	1
*Country*					
US/Canada	23	5	5	8	2
China	21	―	1	1	2
UK/Europe	11	―	1	5	2
Other^c^	16	―	3	10	4
NR	―	―	―	1	―
*Sample sizes (total N)*					
30–99	39	3	8	21	5
100–249	27	2	2	3	4
250–499	5	―	―	―	1
500–999	―	―	―	―	―
> 1,000	―	―	―	1	―
*Study design*					
Cohort/longitudinal	60	5	10	23	9
RCT	11	―	―	2	1
*Analytic methods*					
ROC (or sensitivity/specificity)	61	3	8	24	10
Machine learning	22	―	8	11	4
Models validated	36	―	9	16	5

*Abbreviations*. *DTI* Diffusion tensor imaging, *ECT* Electroconvulsive therapy, *MRI* Magnetic resonance imaging (structural or functional), *NR* Not reported, *ROC* Receiver operating curve, *rTMS* Repetitive transcranial magnetic stimulation, *UK * United Kingdom, *US* United States

^a^Includes 1 study on transcranial direct current stimulation (tDCS), 1 study on theta burst stimulation (TBS) vs. repetitive transcranial magnetic stimulation (rTMS), and 1 using multi-modal inpatient treatment

^b^Includes positron emission tomography (PET), single-photon emission computerized tomography (SPECT), and magnetoencephalography (MEG)

^c^Includes other countries not included in categories above, as well as studies done in multiple countries

Five studies evaluated other neuroimaging and neurophysiologic techniques in response to antidepressants (MEG *k* = 1 and PET *k =* 2), or rTMS (SPECT *k =* 2, conducted by a single research group). Sample sizes were very small (*N* = 33–58). The SPECT studies also included individuals with bipolar disorder.

Finally, two studies used MRI to evaluate prognosis in depressive disorders. One evaluated the association between changes in hippocampal volumes and symptoms over two years for US adults 60 years or older at baseline, diagnosed with depression (*N* = 72) or not (*N* = 90). The second study used task-specific fMRI and machine learning methods to classify symptom trajectories for 118 young and middle-aged Dutch adults (mean age 36–43 years) over two years.

### Other conditions

#### Anxiety disorders

Four studies evaluated diagnosis of anxiety disorders, all using either structural MRI (*k =* 1) or fMRI (*k =* 3; one resting and two task-specific). These addressed general anxiety disorder (*k =* 3), social anxiety disorder (*k =* 1), and panic disorder (*k =* 1). Studies were small (*N* = 40–93) and included young adults with substantial representation of women. All included healthy controls and used the SCID and/or the Hamilton Anxiety Rating Scale (HAM-A) as gold standards. Three studies were cross-sectional and the last was a longitudinal cohort. All assessed model accuracy and three conducted model validation; two used machine learning methods

Six studies evaluated structural MRI (*k* = 2) and fMRI (*k* = 4; all task-specific) in response to psychotherapy (*k* = 4), antidepressants (*k* = 1), and a computer-based behavioral intervention (*k* = 1). These addressed general anxiety disorder (*k* = 4), social anxiety disorder (*k* = 1), and panic disorder (*k* = 2). All but one study had total *N* < 100 (range = 34–135). Participants were young adults, and women were well represented (four studies had more than 50%). All but one undertook model validation and two employed machine learning methods

#### Obsessive compulsive disorder (OCD)

Seventeen studies evaluated diagnosis of OCD, with four using structural MRI, 11 using resting-state fMRI, and one each with DTI and EEG. All studies included young adults and had *N* < 200. Most included healthy controls (*k* = 14) and half had 16–40% women participants (*k* = 10). The most commonly used diagnostic standard was the Yale Brown Obsessive Compulsive Scale (Y-BOCS; *k =* 15), though 11 also used SCID. Nearly all studies evaluated model accuracy (*k =* 14), while seven undertook model validation and five used machine learning.

Only two studies evaluated treatment response in OCD. A Korean study employed baseline structural MRI and machine learning methods to predict response after antidepressants for 4 months in 56 participants. One US study used machine learning methods to examine the association of resting-state fMRI changes with symptoms after four weeks of cognitive behavioral therapy for 42 US adults.

#### Bipolar disorders

Forty-one studies evaluated diagnosis for bipolar disorders; nearly all used MRI-based techniques (*k =* 20 structural MRI, *k =* 18 fMRI [12 resting and six task-specific], *k =* 3 DTI, and *k =* 2 ASL). One study evaluated EEG for diagnosis. Half of studies were small with total sample sizes less than 100 (*k* = 23); only three studies had more than 250 participants (range 251–441). More than half of studies also included participants with unipolar depression (*k =* 27) and healthy controls (*k* = 23). Most were cross-sectional (*k =* 31), while three were longitudinal (to confirm symptoms and diagnosis over 1–2 years). Sixteen studies used structured clinical interviews (MINI and/or SCID) and standardized clinician assessments (Young Mania Rating Scale [YMRS]) as the diagnostic standard. Another 18 studies used only structured interviews, and three used only YMRS. About half of diagnostic studies used machine learning methods (*k =* 25) and undertook model validation (*k =* 24).

All four prognostic studies also included participants with depressive disorders, and thus were discussed above in the section on depressive disorders studies. Briefly, these studies all used MRI-based techniques to evaluate outcomes after ECT, were small (*N* = 33–122), and included middle-aged adults (mean age range 39–56). Three studies used machine learning and validated models.

#### Posttraumatic stress disorder (PTSD)

Thirty studies focused on PTSD, with most evaluating diagnosis (*k* = 24). Fourteen diagnostic studies employed MRI-based techniques, five used EEG, and six used other techniques (*k* = 2 SPECT, *k* = 1 PET, and *k* = 3 MEG [one of these also used MRI]). Most diagnostic studies were small, with the majority having *N* < 100 (*k =* 15). The remaining sample sizes were 116–217 (*k =* 8) and 2,137 for one large database study. Six studies included participants who also had TBI. Of the 24 studies evaluating diagnosis, 11 used structured interviews (SCID) and 12 used clinician assessments (Clinician Administered PTSD Scale [CAPS]) as the diagnostic standard. Some also used patient-reported outcome measures such as the PTSD Checklist (PCL; *k =* 7). Nearly all studies were cross-sectional in design, with only two being cohort studies. All studies evaluated model accuracy, 10 undertook model validation, and nine used machine learning methods.

Six prognostic studies all used either structural MRI (*k =* 2) or fMRI (*k =* 4; two resting and two task-specific). Sample sizes ranged from 53–135, with most having *N* < 100 (*k =* 5). One study included alcohol use disorder (AUD). All studies included relatively young adults and reported on predictive models for response to psychotherapy treatment, using CAPS to assess PTSD severity. One study included both psychotherapy and TMS. One study used machine learning methods, and three assessed model accuracy and performed model validation.

#### Substance use disorders (SUD)

Twenty studies addressed SUD, with the majority focusing on AUD (*k* = 12, 60%). Remaining studies focused on cocaine use disorder (*k* = 3), opioid use disorder (*k* = 2, and methamphetamine use disorder (*k* = 3). About half evaluated diagnosis (*k =* 9), while the rest estimated prediction of relapse (*k =* 6) or treatment response (*k =* 5). Of the nine studies that evaluated diagnostic accuracy, three used MRI-based techniques and six used EEG. Most were small with *N* < 100 (*k* = 6) and all included healthy controls. Six used structured interviews (SCID) and three used patient-reported outcomes (Alcohol Use Disorders Identification Test [AUDIT]) as the diagnostic standard. All studies were cross-sectional and evaluated model accuracy, five undertook model validation, and six used machine learning methods.

Of the 11 prognostic studies, two used structural MRI, six used fMRI (4 resting and 2 task-specific), and one used both in addition to ASL. The remaining two studies used EEG. Most studies were small with *N* < 100 (*k* = 8); one study had a total sample size greater than 1000 (*N* = 1,376). All study samples had less than 40% women and a mean age less than 55 years; only one study reported race. Seven studies assessed model accuracy, three undertook model validation, and five used machine learning methods.

## Discussion

Our evidence map found that the majority of existing studies used MRI-based techniques to evaluate depressive disorders. Anxiety disorders had the smallest number of studies. Most studies were small, focused on diagnosis, and cross-sectional. Even longitudinal studies often lacked important data on history of exposures, symptoms, and treatments. Very few included only participants with their first episode or new onset of symptoms. Additionally, no existing study evaluated prediction of adverse events, despite this being an important factor in patient and clinician decisions to stop or switch therapies. These methodological limitations have been previously noted and likely contribute to poor replicability and validity of precision medicine studies in mental health [6, 43, 44]. Whereas most existing studies included less than 100 participants, current estimates are that thousands of individuals are needed to provide stable and valid results regarding important associations between neuroimaging biomarkers and complex clinical phenotypes [6].

A recent analysis of neural heterogeneity showed that extreme regional variation of brain deviations characterizes mental illness [45], which may explain the well-established clinical heterogeneity of psychiatric disorders [46, 47]. Despite the high degree of neural heterogeneity between individual cases, case-control studies often rely on group mean comparisons that ignore this variation [46–48]. Longitudinal and person-specific data on brain networks, symptoms, and exposures are likely needed to further clarify clinical phenotypes, along with consideration of transdiagnostic dimensional approaches [45, 49–51].

Other challenges include high cost and technical difficulty of assessments and differing measures of symptoms and treatment response [6, 52–54]. Furthermore, to account for changes in brain structure and functioning over time, studies should use comparisons with age-standardized findings developed from large populations [43], instead of small samples of age-matched controls. Recent efforts to systematically collect and examine large neuroimaging datasets have yielded more promising results [6, 43]. Thus, future work may produce insights that improve diagnosis and treatment outcomes in mental health.

In order to use precision medicine approaches to improve mental health treatment, the evidence base needs to be greatly expanded. Specifically, we need better evidence from larger, longitudinal studies, such as the Adolescent Brain Cognitive Development (ABCD) study in the US [55], the UK Biobank [5], and the Human Connectome Project [56]. These large-scale studies require substantial upfront and ongoing investments, as well as fundamental changes in research organization and incentives to promote cooperation and data sharing [53, 54, 57]. It may be possible to build on existing large studies that collect neuroimaging data by adding more detailed assessments of mental health symptoms and relevant exposures, or vice versa. Alternatively, it may be possible to pool results from smaller studies if they used standardized common data models, study protocols, and biomarker assessment techniques in clearly defined clinical populations.

We sought to identify and describe the evidence for a broad range of neuroimaging and neurophysiologic biomarkers in the diagnosis, prognosis, and/or treatment response for a variety of mental health conditions. Although this evidence map makes an important contribution by describing the current evidence base and existing gaps, there are several limitations. As typical for evidence maps, we did not formally assess the quality of included studies or extract detailed results on the analytic models (e.g., control variables or performance metrics). Additionally, we limited our search to English-language studies published in 2010 or later, and thus, our map does not include evidence published in non-English languages or before 2010. Finally, we employed machine learning techniques in identification of relevant studies; it is possible that we missed some eligible studies.

## Conclusions

The evidence base on neuroimaging and neurophysiologic biomarkers for mental health disorders is large but predominantly focused on using MRI to evaluate depressive disorders. Existing studies had substantial methodological limitations, including small sample sizes and cross-sectional designs. These concerns indicate that it is likely premature to apply neuroimaging and neurophysiologic tests to evaluate and treat mental health conditions in clinical settings. Future research is needed to more rigorously evaluate the utility of these biomarkers in the diagnosis and management of mental health conditions across a wide spectrum of patient populations and clinical settings.

## Supplementary Information

Supplementary Material 1. S1 File. Protocol. S2 File. Search Strategy. S3 File. AI Methods Description. S4 File. Included Systematic Reviews by Mental Health Condition and Outcome. S5 File. Detailed Characteristics of Included Primary Studies. S6 File. Supplementary Materials References

## Data Availability

All data generated or analyzed during this study are included in this published article and its supplementary information files.

## Abbreviations

ABCD: Adolescent Brain Cognitive Development
ASL: arterial spin labeling
AUD: Alcohol use disorder
AUDIT: Alcohol Use Disorders Identification Test
BDI: Beck Depression Inventory
CAPS: Clinician Administered Posttraumatic Stress Disorder Scale
CVA: Cerebrovascular accident
DAISY: DistillerSR’s Artificial Intelligence System
DTI: diffusion tensor imaging
EEG: electroencephalogram
ESP: Evidence Synthesis Program
fMRI: functional magnetic resonance imaging
GAD-7: General Anxiety Disorder-7
HAM-A: Hamilton Anxiety Rating Scale
HAM-D/HDRS: Hamilton Depression Rating Scale
k: Number of studies
MADRS: Montgomery-Asberg Depression Rating Scale
MEG: Magnetoencephalography
MINI: Mini-International Neuropsychiatric Interview
MRI: Magnetic resonance imaging
MRS: Magnetic resonance spectroscopy
N: Sample size
OCD: obsessive compulsive disorder
PCL: Posttraumatic Stress Disorder Checklist
PET: Positron emission tomography
PHQ-9: Patient Health Questionnaire-9
ppTMS: Paired pulse transcranial magnetic stimulation
PTSD: Posttraumatic stress disorder
PWI: Perfusion weighted imaging
RCT: Randomized controlled trial
ROC: Receiver operating curve
rTMS: Repetitive transcranial magnetic stimulation
SCID: Structured Clinical Interview for the Diagnostic and Statistical Manual of Mental Disorders (DSM)
SF-36: 36-Item Short Form Survey
sMRI: structural magnetic resonance imaging
SPECT: Single photon emission computed tomography
SUD: substance use disorder
TBI: Traumatic brain injury
TBS: Theta burst stimulation
tDCS: Transcranial direct current stimulation
TMS: Transcranial magnetic stimulation
UK: United Kingdom
US: United States
VA: Department of Veterans Affairs
WHO: World Health Organization
WHODAS: World Health Organization Disability Assessment Schedule
WMH-CIDI: World Mental Health Composite International Diagnostic Intervie
Y-BOCS: Yale Brown Obsessive Compulsive Scale
YMRS: Young Mania Rating Scale
