# Supplementary material for: Neuroimaging and neurophysiologic biomarkers for diagnosis and prognosis of depressive disorders, bipolar disorder, anxiety disorders, obsessive compulsive disorder, posttraumatic stress disorder, and substance use disorder: an evidence map
Source: BMC Psychiatry. 2026 May 6;26:375. doi: 10.1186/s12888-025-07429-4 (PMC13147643; doi:10.1186/s12888-025-07429-4)
Supplement: Supplementary file 1 — Supplementary Material 1. S1 File. Protocol. S2 File. Search Strategy. S3 File. AI Methods Description. S4 File. Included Systematic Reviews by Mental Health Condition and Outcome. S5 File. Detailed Characteristics of Included Primary Studies. S6 File. Supplementary Materials References [file 12888_2025_7429_MOESM1_ESM.pdf]

SUPPLEMENTARY MATERIALS

Table of Contents

Supplementary File 1. Protocol 1

Supplementary File 2. Search Strategy 3

Supplementary File 3. AI Methods Description 5

Supplementary File 4. Included Systematic Reviews by Mental Health Disorder and Outcome 7

Supplementary File 5. Disorders Addressed by Included Primary Studies 8

References 21

Supplementary File 1. Protocol

Project Title: Neuroimaging and Neurophysiologic Biomarkers for Mental Health: An Evidence Map

Project Start Date: March 1, 2022

Review Team: Wei (Denise) Duan-Porter, Kristen Ullman, Adrienne Landsteiner, Katie Sowerby, Maylen Anthony, Caleb Kalinowski, Tim Wilt (Minneapolis, MN ESP)

Content Experts: Michele Spoont, Scott Sponheim, Kelvin Lim, Jose Pardo

Operational Partners: Stuart Hoffman, Vetisha McClair, Suma Muralidhar, Clifford Smith, Emily Hartwell

Review Question: What are the quantity, distribution, and characteristics of evidence assessing the accuracy and utility of neuroimaging and neurophysiologic biomarkers in the diagnosis and clinical management of following conditions:

- Depression
- Anxiety
- Posttraumatic stress disorder (PTSD)
- Substance use disorder (SUD)
- Bipolar disorder
- Traumatic brain injury (TBI)

Databases searched: MEDLINE and Embase using MeSH and text terms for imaging studies and conditions of interest

Types of study to be included: RCTs, observational studies, systematic reviews

Condition or domain being studied: Neuroimaging for diagnosis/prognosis of mental health conditions (as defined by SHA section 305)

Participants/population: Adults ≥18 years of age with the following conditions:

- Depression
- Anxiety (including OCD, phobias, and panic disorders)
- Posttraumatic stress disorder (PTSD)
- Substance use disorder (SUD)
- Bipolar disorder
- Traumatic brain injury (TBI)

Tests of interest:

- Magnetic resonance imaging (MRI)
- Functional magnetic resonance imaging (fMRI)
- Diffusion tensor imagine (DTI)
- Perfusion weighted imaging (PWI)
- Magnetic resonance spectroscopy (MRS)

- Positron emission tomography (PET)
- Single photon emission computed tomography (SPECT)
- Arterial spin labeling (ASL)
- Magnetoencephalography (MEG)
- Evoked potentials and electroencephalogram (EEG)
- Paired pulse transcranial magnetic stimulation (ppTMS)

Comparator(s): N/A

Context: Primary studies, published 2010 or later, with N >=30, of neuroimaging for diagnosis or to provide prognostic information for eligible disorders.

Outcome(s):

Diagnostic accuracy compared with:

- Validated structured clinical interviews (eg, MINI, SCID-5, WHO WMH-CIDI)
- Validated clinician reported instruments (eg, HDRS, HAM-A)
- Patient-reported measures of mental health symptoms (eg, PCL-5, PHQ-9, HADS, BDI, GAD-7, NSI, TBI-QoL)
- Measures of cognition, other psychiatric symptoms (eg, delusions, hallucinations)

Prognosis and treatment response:

- Change in symptoms, cognition, functioning (eg, SF-36, WHODAS)
- Sobriety/abstinence or reduction in substance use (SUD only)
- Recurrence or relapse (study must define criteria and use validated measures)
- Sensitivity (vs lack of response) to treatment
- Self-harm behaviors or suicide risk
- Adverse events and side effects

Data extraction (selection and coding): We extracted the following information from eligible studies:

- N (or # of studies for reviews [k])
- Test(s) studied (eg, MRI, EEG)
- Condition(s) (eg, depression, anxiety)
- Outcomes (diagnostic and/or prognostic; analytic methods)
- Demographics and other population characteristics (eg, age, sex/gender, race, past exposures)
- Genetic information captured (Y/N)

Strategy for data synthesis: Evidence map will summarize available evidence but not to include effect estimates, quality rating of individual studies, or formal synthesis (eg, summary effects and certainty of evidence).

Supplementary File 2. Search Strategy

OVID MEDLINE and EMBASE from January 2010 to September 2023

1	exp Stress Disorders, Post-Traumatic/cl, di, dg, rh, th [Classification, Diagnosis, Diagnostic Imaging, Rehabilitation, Therapy]
2	exp combat disorder/ or (post* stress dis* or ptsd or combat disord*).kw,tw.
3	exp Depression/cl, di, dg, rh, th [Classification, Diagnosis, Diagnostic Imaging, Rehabilitation, Therapy]
4	exp Depressive Disorder/cl, di, dg, rh, th [Classification, Diagnosis, Diagnostic Imaging, Rehabilitation, Therapy]
5	(depress* or dysthymi* or MDD or major-depress* dis* or TRD or TRS).tw,kw.
6	Substance-Related Disorders/cl, di, dg, rh, th [Classification, Diagnosis, Diagnostic Imaging, Rehabilitation, Therapy]
7	((problem adj2 (alcohol or drink$ or drug$ or substance)) or (substance adj2 abuse) or substance adj2disorder or ((alcohol or drug or tobacco) adj2 (abuse or addiction or disorder))).mp.
8	((Subst* adj2 disorder*) or SUD).kw,tw.
9	Anxiety/cl, di, dg, rh, th [Classification, Diagnosis, Diagnostic Imaging, Rehabilitation, Therapy]
10	Anxiety Disorders/cl, di, dg, rh, th [Classification, Diagnosis, Diagnostic Imaging, Rehabilitation, Therapy]
11	(anxiety or anxio* or phobi* or agoraphobi* or panic or neurosis or neuroses or neurotic or psychoneuro* or post-trauma* or stress disorder or obsessi* or compul* or OCD or obsessive compulsive disorde* or generalized anxiety disorder or GAD).tw,kw.
12	Bipolar Disorder/cl, di, dg, rh, th [Classification, Diagnosis, Diagnostic Imaging, Rehabilitation, Therapy]
13	((bipolar adj2 dis*) or manic-depress*).kw,tw.
14	Brain Injuries, Traumatic/cl, di, dg, rh, th [Classification, Diagnosis, Diagnostic Imaging, Rehabilitation, Therapy]
15	(brain injur* or TBI or concuss* or head injur* or post-concuss*).tw,kw.
16	or/1-15
17	exp Functional Neuroimaging/ or functional neuroimaging.tw,kw.
18	exp Magnetic Resonance Imaging/ or (Magnetic Resonance Imaging or MRI or fMRI).tw,kw.
19	(diffusion tensor imag* or DTI).kw,tw.
20	(voxel-based morphometry or VBM).tw,kw.
21	(tractograph* or tractometr*).kw,tw.
22	(arterial spin label* or ASL).tw,kw.
23	exp tomography, emission-computed/ or ((positron* adj4 tomograph*) or emission-computed or single photon or SPECT).tw,kw.
24	exp magnetic resonance spectroscopy/ or (magnetic resonance spectroscopy or MR spectroscopy or MRS).tw,kw.
25	exp electroencephalography/ or (electroencephalograph* or EEG).tw,kw.
26	exp magnetoencephalography/ or (magnetoencephalograph* or MEG).tw,kw.
27	exp Evoked Potentials/ or (evoked response* or evoked potential*).tw,kw.
28	or/17-27
29	16 and 28
30	((animal or animals or canine* or cat or cats or dog or dogs or feline or goat or hamster* or horse or lamb or lambs or mice or monkey or monkeys or mouse or murine or pig or pigs or piglet* or porcine

	or primate* or rabbit* or rats or rat or rodent* or sheep* or veterinar*) not (human* or patient*)).ti,kf,jw.
31	(Animals/ or Models, Animal/ or Disease Models, Animal/) not Humans/
32	30 or 31
33	29 not 32
34	limit 33 to (english language and yr="2010 - 2022")
35	limit 34 to (addresses or autobiography or bibliography or biography or case reports or comment or dictionary or directory or editorial or interactive tutorial or interview or legal cases or legislation or news or newspaper article or patient education handout or periodical index or personal narratives or portraits or video-audio media or webcasts)
36	34 not 35
37	limit 36 to (juvenile or infan$ or child$)
38	36 not 37

Supplementary File 3. AI Methods Description

Study Selection

After duplicates were removed, citations were uploaded into DistillerSR (Evidence Partners, Ottawa, Canada). Abstracts were screened with the assistance of DistillerSR's Artificial Intelligence System (DAISY) in 2 separate phases. In the first phase, 2 reviewers were required to exclude an abstract at screening (while only 1 reviewer was needed to include for full-text review) until the DAISY-predicted score for likelihood of inclusion was less than 0.4 and the inclusion rate had fallen to less than 5%. Approximately 12,000 abstracts were reviewed in phase 1. In the second phase, for abstracts with DAISY-predicted scores for likelihood of inclusion of 0.2–0.3 ($k \approx$ 7,000 abstracts), 1 reviewer decided on inclusion for full-text review. The remaining abstracts with DAISY-predicted likelihood scores less than 0.2 were not further reviewed for eligibility (k = 25,912). Based on an inclusion rate of 0.00066 for the last batch of abstracts evaluated during phase 2 (k = 1,526), we conservatively estimate that an additional 17 abstracts may have been potentially included for full-text review, if we had continued with 1-reviewer evaluation of those abstracts with likelihood scores of less than 0.2.

For full-text review, we undertook 2 initial pilot rounds where 5-6 reviewers separately determined eligibility for 10–15 articles in each round. We discussed articles to reach consensus on eligibility, with further clarification on operationalization of inclusion and exclusion criteria. Eligibility of remaining articles was determined by 1 reviewer, with ~50% of these also undergoing evaluation by a second reviewer.

Eligible populations included adults (≥18 years of age) with at least 1 of the conditions of interest, as noted in KQ above. Eligible articles also evaluated at least 1 neuroimaging or neurophysiological test of interest (*eg*, magnetic resonance imaging [MRI], including functional MRI [fMRI], diffusion tensor imaging [DTI], positron emission tomography [PET], single photon emission computed tomography [SPECT], and evoked potentials and electroencephalogram [EEG]) for diagnostic accuracy, clinical prognosis, and/or treatment response. Exclusion criteria included pediatric populations, evaluation of mental health symptoms or cognitive functioning only in the context of neurodegenerative conditions (*eg*, Alzheimer's dementia or Parkinson's disease) or intracranial injury (*eg*, due to ischemic or hemorrhagic stroke). We also excluded studies attempting to evaluate prognostic patterns using exclusively cross-sectional data (*eg*, comparing current differences in neuroimaging or neurophysiological patterns between patients with depression in remission vs those with treatment-resistant depression). There are very substantial validity concerns with use of cross-sectional data to evaluate predictors of treatment response or general prognosis, which has been noted previously.[2]

Data Abstraction

We abstracted the following data from all eligible studies: population characteristics (*eg*, condition and method of diagnosis, sample size, demographic data (*eg*, mean or median age, proportion of women, focus on Veterans or combat exposure), neuroimaging test and/or EEG being evaluated (and genetic data if used), outcomes addressed (clinical diagnosis and/or prognosis), and study design (*eg*, cross-sectional or cohort, analytic methods used to assess diagnostic accuracy). To verify accuracy of abstracted results, data from ~50% of articles were over-read by a second reviewer.

Quality Assessment and Summary of Results

We did not conduct formal quality assessment of eligible studies included in this report. We also did not undertake a formal synthesis of study results. Our results summaries are organized by the conditions of interest and focus on describing the characteristics of study populations, outcomes (clinical diagnosis, prognosis, and/or treatment response), and study designs (including analytic methods) of eligible studies.

Peer Review
A draft version of this report was reviewed by technical experts as well as clinical leadership.

Supplementary File 4. Included Systematic Reviews by Mental Health Disorder and Outcome

Condition	Author, Year	# Included studies	Diagnosis	Prognosis	
				Response to Treatment	Change in Symptoms or Functioning
Depression	Bruun, 2021[3]	24	X		
	Cohen, 2021[4]	27		X	
	De Crescenzo, 2017[5]	11		X	
	Dichter, 2015[6]	21		X	
	Enneking, 2020[7]	50		X	
	Fu, 2013[8]	20		X	
	Gillett, 2020[9]	21		X	
	Khosla, 2022[10]	132	X	X	
	Levy, 2019[11]	19		X	
	Long, 2020[12]	17		X	
	Masse-Sibille, 2018[13]	58		X	
	Scheepens, 2020[14]	14	X	X	
	Siegel-Ramsay, 2022[15]	88	X		
	Simon, 2021[16]	12	X		
	Sinha, 2020[17]	13	X		X
	van der Vinne, 2017[18]	16	X		
	Widge, 2019[19]	76	X		
Bipolar disorders	Hozer, 2016[20]	63	X	X	
	Librenza-Garcia, 2017[21]	51	X		
	Seeberg, 2018[22]	60		X	
	Whalley, 2012[23]	21	X		
PTSD	Colvonen, 2017[24]	20		X	
	Nelson, 2017[25]	37			X
OCD	Fullana, 2020[26]	352*	X		
Anxiety disorders	Qing, 2021[27]	11	X		
	Santos, 2019[28]	24		X	
	Xu, 2019[29]	29	X		

Supplementary File 5. Disorders Addressed by Included Primary Studies

Author, Year	Condition Studied					
	Depressive Disorders	Bipolar Disorder	Anxiety Disorders	OCD	PTSD	SUD
Achalia, 2020[30]		X				
Adinoff, 2015[31]						X
Almeida, 2013[32]	X	X				
Altuglu, 2020[33]				X		
Ambrosi, 2017[34]	X	X				
Amen, 2017[35]	X					
Amen, 2015[36]					X	
Arns, 2014[37]	X					
Arns, 2012[38]	X					
Arribas, 2010[39]		X				
Bachmann, 2017[40]	X					
Bailey, 2018[41]	X					
Baranger, 2021[42]	X					
Bares, 2019[43]	X					
Bares, 2017[44]	X					
Bares, 2015[45]	X					
Bartlett, 2018[46]	X					
Baskaran, 2018[47]	X					
Bi, 2019[48]	X					
Bi, 2018[49]	X					
Bi, 2016[50]	X					
Brandt, 2021[51]	X					
Braund, 2022[52]	X					
Bruin, 2021[53]	X	X				
Burger, 2017[54]	X	X				
Camchong, 2021[55]						X
Cash, 2019[56]	X					
Chen, 2022[57]	X					
Chen, 2021[58]				X		
Chen, 2020[59]	X					
Chen, 2021[60]	X					
Cheng, 2017[61]	X					
Chin Fatt, 2020[62]	X					
Colle, 2015[63]	X					
Cook, 2020[64]	X					

Author, Year	Condition Studied					
	Depressive Disorders	Bipolar Disorder	Anxiety Disorders	OCD	PTSD	SUD
Cook, 2013[65]	X					
Costafreda, 2011[66]		X				
Crane, 2017[67]	X					
Crowther, 2015[68]	X					
Cui, 2020[69]				X		
Dai, 2021[70]						X
de la Salle, 2020[71]	X					
Deng, 2018[72]	X	X				
Ding, 2019[73]	X					
Drysdale, 2017[74]	X					
Duan, 2020[75]	X					
Dunlop, 2017[76]	X					
Durazzo, 2017[77]						X
Ellard, 2018[78]	X	X				
Erguzel, 2020[79]						X
Erguzel, 2019[80]						X
Erguzel, 2015[81]	X					
Erguzel, 2014[82]	X					
Etkin, 2019[83]					X	
Fan, 2022[84]	X					
Fang, 2012[85]	X					
Farb, 2022[86]	X					
Feder, 2017[87]	X					
Fonzo, 2017[88]					X	
Frangou, 2017[89]	X	X				
Frick, 2020[90]			X			
Gao, 2021[91]	X					
Gao, 2022[92]	X					
Gartner, 2018[93]	X					
Ge, 2020[94]	X					
Ge, 2019[95]	X					
Georgopoulos, 2010[96]					X	
Godlewska, 2018[97]	X					
Godlewska, 2016[98]	X					
Goldstein-Piekarski, 2018[99]	X					
Gong, 2014[100]					X	

Author, Year	Condition Studied					
	Depressive Disorders	Bipolar Disorder	Anxiety Disorders	OCD	PTSD	SUD
Gong, 2014[101]					X	
Gong, 2011[102]	X					
Gosnell, 2019[103]	X					
Gowin, 2015[104]						X
Grieve, 2016[105]	X					
Grotegerd, 2014[106]	X	X				
Guo, 2020[107]	X					
Guo, 2018[108]	X					
Guo, 2012[109]	X					
Guo, 2012[110]	X					
Gyurak, 2016[111]	X					
Hahn, 2015[112]			X			
Hahn, 2011[113]	X					
Hasanzadeh, 2020[114]	X					
Hasanzadeh, 2019[115]	X					
He, 2019[116]	X	X				
Hellewell, 2019[117]	X					
Hopman, 2021[118]	X					
Hou, 2021[119]	X					
Hou, 2018[120]	X					
Hou, 2018[121]	X					
Hou, 2016[122]	X					
Hu, 2019[123]	X					
Hu, 2019[124]				X		
Hu, 2016[125]				X		
Ichikawa, 2020[126]	X					
Im, 2017[127]					X	
Isserles, 2018[128]	X					
James, 2022[129]					X	
Januszko, 2021[130]						X
Jaworska, 2018[131]	X					
Jaworska, 2014[132]	X					
Jaworska, 2013[133]	X					
Jiang, 2021[134]	X					
Jiang, 2020[135]	X					
Jiang, 2018[136]	X					

Author, Year	Condition Studied					
	Depressive Disorders	Bipolar Disorder	Anxiety Disorders	OCD	PTSD	SUD
Kamarajan, 2020[137]						X
Karim, 2018[138]	X					
Kaufman, 2015[139]	X					
Kim, 2020[140]					X	
Kinreich, 2021[141]						X
Kipli, 2015[142]	X					
Klumpp, 2020[143]	X		X		X	
Klumpp, 2017[144]			X			
Koller-Schlaud, 2020[145]	X					
Koo, 2019[146]	X					
Korgaonkar, 2020[147]	X					
Korgaonkar, 2015[148]	X					
Korgaonkar, 2014[149]	X					
Korgaonkar, 2012[150]	X					
Kraus, 2019[151]	X					
Kwak, 2020[152]				X		
Lanka, 2020[153]					X	
Laxminarayan, 2020[154]					X	
Leaver, 2018[155]	X					
Lebedeva, 2017[156]	X					
Lee, 2011[157]	X					
Li, 2021[158]	X					
Li, 2021[159]	X					
Li, 2021[160]	X					
Li, 2020[161]	X					
Li, 2020[162]		X				
Li, 2020[163]	X					
Li, 2019[164]	X					
Li, 2019[165]						X
Li, 2017[166]	X	X				
Li, 2016[167]	X					
Li, 2014[168]				X		
Li, 2021[169]	X					
Liao, 2018[170]	X					
Liu, 2022[171]	X					

Author, Year	Condition Studied					
	Depressive Disorders	Bipolar Disorder	Anxiety Disorders	OCD	PTSD	SUD
Liu, 2021[172]				X		
Liu, 2021[173]				X		
Liu, 2020[174]				X		
Liu, 2020[175]	X					
Liu, 2020[176]	X					
Liu, 2015[177]					X	
Liu, 2015[178]			X			
Liu, 2014[179]	X	X				
Liu, 2012[180]	X					
Lord, 2012[181]	X					
Lu, 2021[182]		X				
Lu, 2013[183]	X					
Luo, 2021[184]				X		
Lv, 2021[185]				X		
Manelis, 2020[186]	X	X				
Matsuo, 2019[187]	X					
Matsuoka, 2017[188]	X					
McHugh, 2014[189]						X
Meng, 2020[190]	X					
Meyer, 2019[191]	X					
Mishra, 2020[192]						X
Modinos, 2013[193]	X					
Mohammadi, 2015[194]	X					
Mourao-Miranda, 2012[195]	X					
Mulders, 2020[196]	X					
Mumtaz, 2019[197]	X					
Mumtaz, 2018[198]	X					
Mumtaz, 2018[199]						X
Mumtaz, 2018[200]	X					
Mumtaz, 2017[201]	X					
Mumtaz, 2017[202]						X
Mumtaz, 2017[203]	X					
Mwangi, 2016[204]		X				
Neumeister, 2013[205]					X	
Nguyen, 2019[206]	X					
Nicholson, 2019[207]					X	

Author, Year	Condition Studied					
	Depressive Disorders	Bipolar Disorder	Anxiety Disorders	OCD	PTSD	SUD
Niida, 2018[208]		X				
Niida, 2012[209]	X	X				
Nogovitsyn, 2020[210]	X					
Nord, 2019[211]	X					
Olbrich, 2012[212]	X					
Oliveira-Maia, 2017[213]	X					
Palaniyappan, 2022[214]	X					
Pang, 2020[215]	X	X				
Pantazatos, 2014[216]			X			
Patel, 2015[217]	X					
Pillai, 2019[218]	X					
Price, 2018[219]			X			
Qiao, 2017[220]			X			
Qin, 2022[221]	X					
Qin, 2015[222]	X					
Qin, 2014[223]	X					
Qiu, 2014[224]	X					
Rabinoff, 2011[225]	X					
Raji, 2015[226]					X	
Rangaprakash, 2018[227]					X	
Rangaprakash, 2017[228]					X	
Rangaprakash, 2019[229]					X	
Redlich, 2014[230]	X	X				
Reggente, 2018[231]				X		
Rentzsch, 2014[232]	X					
Richieri, 2018[233]	X	X				
Richieri, 2011[234]	X	X				
Rive, 2016[235]	X	X				
Rocha-Rego, 2014[236]		X				
Rottstaedt, 2018[237]	X					
Rubin-Falcone, 2018[238]	X	X				
Sacchet, 2015[239]	X	X				
Sadat Shahabi, 2021[240]	X					

Author, Year	Condition Studied					
	Depressive Disorders	Bipolar Disorder	Anxiety Disorders	OCD	PTSD	SUD
Sankar, 2016[241]	X					
Schmaal, 2015[242]	X					
Schnack, 2014[243]		X				
Schnyer, 2017[244]	X					
Schultz, 2018[245]	X					
Sekutowicz, 2019[246]						X
Serpa, 2014[247]	X	X				
Shalbaf, 2018[248]	X					
Shan, 2020[249]		X				
Shan, 2021[250]	X					
Shao, 2019[251]	X	X				
Shi, 2021[252]	X					
Shi, 2018[253]	X	X				
Shim, 2019[254]	X				X	
Shimizu, 2015[255]	X					
Shu, 2014[256]					X	
Siegle, 2012[257]	X					
Squarcina, 2019[258]		X				
Stange, 2020[259]	X	X				
Stout, 2021[260]					X	X
Stoyanov, 2019[261]	X					
Sun, 2021[262]	X					
Sun, 2022[263]	X					
Sun, 2022[264]	X	X				
Sun, 2020[265]	X	X				
Suo, 2020[266]					X	
Sverdlov, 2021[267]	X					
Tahmasian, 2017[268]					X	
Takagi, 2017[269]				X		
Tang, 2022[270]	X	X				
Taylor, 2014[271]	X					
Tekin Erguzel, 2015[272]	X	X				
Tenke, 2011[273]	X					
Tian, 2020[274]	X					
Tsolaki, 2021[275]	X	X				
Uyulan, 2022[276]	X					

Author, Year	Condition Studied					
	Depressive Disorders	Bipolar Disorder	Anxiety Disorders	OCD	PTSD	SUD
van Rooij, 2016[277]					X	
van Waarde, 2015[278]	X					
Voineskos, 2019[279]	X					
Wade, 2017[280]	X					
Wade, 2016[281]	X	X				
Wade, 2017[282]	X					
Wang, 2022[283]	X					
Wang, 2021[284]	X					
Wang, 2020[285]		X				
Wang, 2019[286]				X		
Wang, 2019[287]	X					
Wang, 2017[288]	X					
Wang, 2017[289]	X					
Whitfield-Gabrieli, 2016[290]			X			
Williams, 2015[291]	X					
Wu, 2020[292]	X					
Wu, 2021[293]	X					
Wu, 2021[294]	X					
Wu, 2017[295]		X				
Xi, 2022[296]	X	X				
Xiao, 2021[297]	X					
Xing, 2020[298]			X			
Xue, 2021[299]	X					
Yan, 2020[300]	X					
Yan, 2022[301]	X					
Yan, 2021[302]	X					
Yan, 2021[303]						X
Yan, 2021[304]	X					
Yang, 2022[305]				X		
Yang, 2019[306]				X		
Yang, 2018[307]	X					
Yang, 2018[308]	X					
Yang, 2019[309]		X				
Yang, 2016[310]	X					
Yang, 2021[311]	X	X				
Yeh, 2015[312]	X					

Author, Year	Condition Studied					
	Depressive Disorders	Bipolar Disorder	Anxiety Disorders	OCD	PTSD	SUD
Yoshida, 2017[313]	X					
Yu, 2018[314]	X					
Yun, 2015[315]				X		
Zehong, 2019[316]	X					
Zeng, 2012[317]	X					
Zhai, 2021[318]						X
Zhang, 2022[319]	X					
Zhang, 2022[320]	X					
Zhang, 2021[321]	X					
Zhang, 2020[322]					X	
Zhang, 2016[323]					X	
Zhao, 2020[324]	X					
Zhao, 2017[325]	X	X				
Zhdanov, 2020[326]	X					
Zheng, 2019[327]	X					
Zhong, 2017[328]	X					
Zhou, 2018[329]				X		
Zhu, 2021[330]					X	
Zhu, 2021[331]	X					
Zhu, 2020[332]					X	
Zhu, 2018[333]						X
Zhu, 2018[334]	X					
Zhu, 2019[335]	X					
Zhutovsky, 2019[336]					X	

TOTALS →	441	441	441	239	320	61	22	12	6	6	26	39	25	16	3	3	3	134	174	135	41	16	4	120	24	6	5	13	441	303	32	130	24	295	192	186	102	251	285	202	152	132	85	8	13	132	138	133
			STUDY DESIGN		CONDITION STUDIED													NEUROIMAGING TEST(S) USED											POPULATION CHARACTERISTICS					EVALUATED DIAGNOSTIC ACCURACY	DIAGNOSTIC STANDARD			ANALYTIC METHODS			EVALUATED PROGNOSIS, CHANGE OR RESPONSE	ANALYTIC METHODS		PROGNOSIS OUTCOME				
							Anxiety Disorders						Substance Use Disorders						fMRI																													
FIRST AUTHOR	YEAR	COUNTRY	Cross-sectional vs. Cohort vs. RCT	Machine Learning Approach	Depressive Disorders	Bipolar Disorder	Any	GAD	SAD	Panic	OCD	PTSD	Any	Alcohol	Opioid	Meth	Cocaine	Struct. MRI	Any	Resting	Task	DTI	ASL	Evoked Potentials & EEG	Other	PET	SPECT	MEG	Total N	Healthy Controls	Treatment Resistant	Medication Free	First Episode		Structured Clinical Interview	Other Clinician Assessments	Patient Reported Outcome	Sensitivity/ specificity (or PPV/NPV)	Model Accuracy Assessed	Model Validated		Model Accuracy Assessed	Model Validated	Change in Symptoms Description	Relapse Description	Sensitivity to Treatment Description	Treatment Type	Measure of Response
Achalia	2020	India	Cross-sectional			X													X	X		X							60	X				X	X	X		X	X									
Adamson	2022	US	Cohort/Longitudinal		X																			X					50												X					response (≥50% BDI-II reduction)	rTMS	PRO
Aderinwale	2023	South Korea	Cohort/Longitudinal		X		X			X														X					149	X				X			X		X	X								
Adinoff	2015	US	Cohort/Longitudinal										X				X	X	X	X			X						60	X		X									X	X			relapse (self-reported or UDS-detected use of a stimulant) vs early remission			
Akbari	2022	UK	Cohort/Longitudinal		X																			X					44	X				X				X	X	X								
Al-Ezzi	2023	Malaysia	Cross-sectional	X			X		X															X					88	X		X		X		X		X										
Ali	2022	US	Cohort/Longitudina	X	X																				X	X			60												X	X	X			remission (HDRS≤7)	Antidepress.	Clinician assessment
Almeida	2013	US	Cross-sectional	X	X	X																	X						36					X	X			X	X	X								
Altuglu	2020	Turkey	Cohort/Longitudina	X							X													X					57		X			X	X		X					X	X					
Alyoubi	2023	Saudi Arabia	Cohort/Longitudina	X	X																			X					64	X				X	X			X	X	X								
Ambrosi	2017	US	Cross-sectional		X	X												X											116	X				X	X		X	X	X									
Amen	2017	US	Cross-sectional		X																				X		X		4541					X	X		X	X	X	X								
Amen	2015	US	Cross-sectional									X													X		X		397	X				X	X			X	X									
Andreev	2023	Russia	Cohort/Longitudina	X	X														X	X									85	X				X	X	X			X	X								
Arns	2014	NR	Cohort/Longitudinal		X																			X					90												X	X				response (>50% BDI reduction) or remission (≤12 BDI)	rTMS	PRO
Arns	2012	Netherlands	Cohort/Longitudinal		X																			X					90												X	X				remission (BDI </= 12) or response (<50% BDI reduction)	rTMS	PRO
Arribas	2010	US	Cross-sectional	X		X													X		X								61	X				X	X			X	X	X								
Bachmann	2017	Estonia	Cross-sectional		X																			X					34	X		X		X		X		X	X									
Bailey	2018	Australia	Cohort/Longitudina	X	X																			X					59	X	X										X	X	X			response	rTMS	Clinician assessment
Baranger	2021	US	Cross-sectional	X	X													X											86	X				X	X	X		X	X	X								
Bares	2019	Czech republic	Cohort/Longitudinal		X																			X					103												X	X				response (≥50% MADRS reduction)	Antidepress.	Clinician assessment
Bares	2017	Czech Republic	Cohort/Longitudinal		X																			X					38		X										X	X				response (≥50% MADRS reduction)	Antidepress.	Clinician assessment
Bares	2015	Czech Republic	Cohort/Longitudinal		X																			X					87		X										X	X				response (≥50% MADRS reduction)	Antidepress.	Clinician assessment
Bartlett	2018	US	RCT	X	X													X											184			X									X	X	X			response	Antidepress.	Clinician assessment
Baskaran	2018	Canada	Cohort/Longitudinal		X																			X					44												X	X				response (≥50% MADRS reduction)	Antidepress.	Clinician assessment
Beliveau	2022	Denmark	Cohort/Longitudinal		X													X											79			X									X	X	X			response (≥50% HAMD-6 reduction) and remission (HAMD≤4)	Antidepress.	Clinician assessment
Benschop	2022	Belgium	Cross-sectional		X																			X					40	X				X	X	X	X	X	X									
Berwian	2023	Germany, Switzerla	Cohort/Longitudinal		X																			X					91	X											X	X	X		fulfilling criteria for MDD episode according to DSM-IV		Antidepress.	Clinician assessment + interview. PRO
Bi	2019	China	Cross-sectional		X																	X			X			X	48	X				X	X	X		X	X	X								
Bi	2018	China	Cross-sectional		X													X							X			X	108	X				X	X				X	X								
Bi	2016	China	Cross-sectional	X	X																	X			X			X	52	X		X		X	X	X		X	X	X								
Brandt	2021	Denmark	Cohort/Longitudinal		X														X		X								148	X		X									X	X	X			response (≥50% HAMD reduction) or remission (≤7 HAMD)	Antidepress.	Clinician assessment
Braund	2022	Australia	RCT		X														X		X								294	X											X	X				response (≥50% HRSD/QIDS-SR16 reduction) or remission (≤7 HRSD/≤5 QIDSSR16)	Antidepress.	Clinician assessment, PRO
Bruin	2021	US, Belgium, Denm	Cohort/Longitudina	X	X	X												X	X	X									189		X										X	X	X			response (≥50% HAMD reduction) or remission (≤7 HAMD)	ECT	Clinician assessment
Burger	2017	Germany	Cross-sectional	X	X	X													X		X								108	X				X	X	X	X	X	X	X								
Camchong	2021	US	Cohort/Longitudinal										X	X					X	X									45												X				relapse at 11-mo follow-up			
Cash	2019	Australia	Cohort/Longitudina	X	X														X	X									76	X											X	X	X			response (MADRS)	rTMS	Clinician assessment
Chakravarthi	2022	NR	Cohort/Longitudina	X								X												X					NR					X					X									
Chang	2023	Korea	Cross-sectional		X																			X					93	X				X	X		X	X	X									
Chen	2022	China	Cross-sectional	X	X														X	X									94	X		X		X		X		X	X	X								
Chen	2021	China	Cross-sectional	X							X							X											78	X		X		X	X		X	X	X									
Chen	2020	Taiwan	Cross-sectional		X																			X					36	X				X	X	X		X	X	X								
Chen	2021	China	Cross-sectional	X	X													X											186	X				X		X		X	X	X								
Chen	2022	Russia	Cross-sectional		X														X	X									70	X				X			X	X										
Chen	2022	China	Cross-sectional			X												X											50												X	X	X					
Chen	2023	China	Cross-sectional	X	X													X	X	X									362	X		X		X		X	X	X	X	X								
Chen	2023	China	Cohort/Longitudinal		X	X													X	X									40												X	X		suicidal ideation		response (0 on Beck SSI-part I [day 13], indicating no suicidal ideation)	Ketamine	PRO
Cheng	2017	China	Cohort/Longitudinal		X													X											148	X	X	X									X	X				response (≥50% HAMD reduction) or remission (≤7 HAMD)	Antidepress.	Clinician assessment
Chin Fatt	2020	US	RCT		X														X	X									317	X		X									X	X	X			response	Antidepress.	Clinician assessment
Cirstian	2023	Netherlands	Cross-sectional		X														X	X									72	X		X		X		X	X	X	X	X								
Colle	2015	France	Cross-sectional		X													X											63					X	X	X		X	X									
Cook	2020	US	Cohort/Longitudinal		X																			X					133			X									X	X	X			remission (≤7 HRSD)	Antidepress.	Clinician assessment
Cook	2013	US	RCT		X																			X					220												X	X				sustained remission (≤7 HRSD for that and all subsequent visits)	Antidepress.	Clinician assessment
Costafreda	2011	UK	Cross-sectional			X													X		X								72	X				X		X	X	X	X									
Crane	2017	US	Cohort/Longitudinal		X														X		X								36			X									X	X	X			response (HDRS)	Antidepress.	Clinician assessment
Crowther	2015	US	Cohort/Longitudinal		X														X	X									43	X		X									X					change in BDI	Psychotherapy	PRO
Cui	2020	China	Cross-sectional								X								X	X									78	X		X		X	X	X	X	X	X									
Dai	2021	China	Cross-sectional	X									X	X				X											80	X				X	X			X	X	X								
Dai	2023	China	Cross-sectional		X													X	X	X									615	X				X	X			X	X	X								
de la Salle	2020	Canada	Cohort/Longitudinal		X																			X					47			X									X	X				response (≥50% MADRS reduction)	Antidepress.	Clinician assessment
Deng	2018	China	Cross-sectional	X	X	X																X							112	X		X		X	X	X		X	X									
Ding	2019	China	Cross-sectional	X	X																			X					348	X				X	X			X	X	X								
Ding	2023	China	Cross-sectional								X							X	X	X									100	X		X		X	X	X	X	X	X	X								
Drysdale	2017	Canada, Japan, Ch	Cohort/Longitudina	X	X														X	X									1188	X	X			X	X	X	X	X	X	X	X	X	X			response (>25% HAMD reduction) or full response (>50% reduction)	rTMS	Clinician assessment
Duan	2020	China	Cross-sectional	X	X																			X					32	X				X	X			X	X	X								
Dunlop	2017	US	RCT		X														X	X									122			X									X	X				remission (≤7 HAMD), failure (<30% HAMD reduction), response (≥50% HAMD reduction), partial response (30-49% HAMD reduction)	Antidepress. or psychotherapy	Clinician assessment
Durazzo	2017	US	Cohort/Longitudinal										X	X				X											129												X				relapse (alcohol use reported by participant, relative/close friend, or medical records)			
Ebrahimzadeh	2023	Iran	Cohort/Longitudina	X	X																			X					88												X	X	X			response (>50% BDI-II/HRSD reduction) or remission (BDI≤8/HRSD≤7)	rTMS	Clinician assessment, PRO
Ellard	2018	US	Cross-sectional		X	X												X											98	X				X	X	X		X										
Ellis	2023	Malaysia	Cohort/Longitudina	X	X																			X					58	X				X	X			X	X	X								
Emre	2023	NR	Cohort/Longitudina	X	X						X	X	X		X									X					257	X				X		X		X	X	X								
Erguzel	2020	Turkey	Cross-sectional										X		X									X					134	X				X	X				X									
Erguzel	2019	Turkey	Cross-sectional	X									X		X									X					134	X				X	X				X	X								
Erguzel	2015	Turkey	Cohort/Longitudinal		X																			X					55			X			X	X					X	X	X			response (≥50% HAMD reduction)	rTMS	Clinician assessment
Erguzel	2014	Turkey	Cohort/Longitudina	X	X																			X					147		X	X									X	X	X			response (HAMD)	rTMS	Clinician assessment
Etkin	2019	US	Cohort/Longitudina	X								X							X	X									66	X		X									X	X	X			response (≥50% CAPS reduction)	spTMS	
Fan	2022	China	Cohort/Longitudina	X	X																			X					92												X	X	X			response (HAMD)	Acupuncture	Clinician assessment
Fan	2023	China	Cross-sectional				X	X											X	X									75	X				X	X		X	X	X	X								
Fang	2012	China	Cohort/Longitudina	X	X													X											48	X				X	X	X		X	X	X								
Fang	2023	China	Cross-sectional	X	X														X	X									688	X				X	X	X	X	X	X	X								
Farb	2022	US, Canada	Cohort/Longitudinal		X														X		X								184												X	X			time to relapse/recurrence (SCID)		psychotherapy	Clinician interview
Feder	2017	Germany	Cross-sectional	X	X														X	X									720	X				X	X	X	X		X									
Fisher	2022	Denmark	Cohort/Longitudina	X	X														X		X								204	X		X									X	X	X			remission (>50% HAMD-6 reduction at wk 4 *and* HAMD<5 at wk 8) and response (>25% HAMD-6 reduction at wk 4 and >50% HAMD reduction at wk 8)	Antidepress.	Clinician assessment
Fonzo	2017	US	RCT									X							X		X								66												X	X	X			response (unspecified clinical assessment)	spTMS	
Frangou	2017	US	Cohort/Longitudina	X	X	X													X		X								120	X				X	X	X		X	X	X								
Frick	2020	Sweden	Cohort/Longitudina	X			X	X										X											47			X									X	X	X			response (≥2 CGI-I)	Antidepress.	Clinician assessment
Gai	2023	China	Cross-sectional		X														X	X									475	X		X		X		X		X	X	X								
Gallo	2023	23 cohorts from ac	Cross-sectional	X	X														X	X									2338	X				X	X			X	X	X								
Gao	2021	China	Cross-sectional	X	X													X											432	X			X	X		X		X	X	X								
Gao	2022	China	Cohort/Longitudina	X	X														X	X									98	X		X									X	X	X			response (≥50% HAMD reduction)	Antidepress.	Clinician assessment
Gao	2023	China	Cohort/Longitudina	X		X													X	X									126	X		X		X	X	X	X	X	X	X								
Gao	2023	China	Cross-sectional		X														X	X									146			X		X		X		X	X	X								
Gartner	2018	Germany	Cross-sectional	X	X														X		X								118	X				X	X		X	X	X	X								
Ge	2020	Canada	Cohort/Longitudinal		X														X	X									50		X										X	X	X			response (≥50% HRSD reduction) or remission (<8 HRSD)	rTMS	Clinician assessment
Ge	2019	Canada	Cohort/Longitudinal		X													X	X	X									92	X	X										X	X				remission (≤7 HDRS)	rTMS	Clinician assessment
Ge	2022	Canada	Cross-sectional		X														X	X									38		X										X	X	X	Change in MADRS			rTMS	Clinician assessment
Georgopoulos	2010	US	Cross-sectional	X								X													X			X	324	X				X	X	X			X	X								
Godlewska	2018	UK	Cohort/Longitudinal		X														X		X								32			X									X	X	X			response (≥50% HAMD reduction)	Antidepress.	Clinician assessment
Godlewska	2016	UK	Cohort/Longitudinal		X														X		X								64	X		X									X					response (HAMD)	Antidepress.	Clinician assessment
Goldstein-Pieka	2018	US	RCT		X													X											181	X		X									X	X	X			remission (≤7 HRSD)	Antidepress.	Clinician assessment
Gong	2014	China	Cross-sectional									X							X	X									121					X			X		X									
Gong	2014	China	Cross-sectional									X						X											150	X				X		X	X	X	X									
Gong	2011	China, UK	Cohort/Longitudina	X	X													X											69	X		X		X	X	X		X	X	X	X	X	X			response (≥50% HRSD reduction)	Antidepress.	Clinician assessment
Gosnell	2019	US	Cohort/Longitudinal		X																	X							175										X		X	X				treatment resistance (PHQ-9 ≥9)	Mixed	PRO
Gowin	2015	US	Cohort/Longitudina	X									X			X			X		X								61												X	X			relapse prediction			
Grieve	2016	US, Canada, UK, S	Cohort/Longitudinal		X																	X							157			X									X	X	X			remission (≤7 HDRS)	Antidepress.	Clinician assessment
Grotegerd	2014	Germany	Cross-sectional	X	X	X													X		X								66	X				X	X	X		X	X	X								
Guo	2020	China	Cross-sectional	X	X													X											117	X				X	X			X	X	X								
Guo	2018	China	Cross-sectional	X	X													X											143	X		X	X	X	X	X		X	X	X								
Guo	2012	China	Cross-sectional	X	X													X											66	X		X	X	X	X	X			X	X								
Guo	2012	China	Cross-sectional		X														X	X									64	X	X			X	X	X		X										
Guo	2023	China	Cross-sectional	X	X														X	X									101	X	X			X	X	X		X	X	X								
Gyurak	2016	Australia	RCT		X														X		X								124	X		X									X	X				response (≥50% HRSD reduction) or remission (≤7 HRSD)	Antidepress.	Clinician assessment
Hahn	2015	Germany	Cohort/Longitudina	X			X			X									X		X								49			X									X	X	X			response (HAMA)	psychotherapy	Clinician assessment
Hahn	2011	Germany	Cross-sectional	X	X													X											60	X				X			X	X	X	X								
Han	2023	China	Cohort/Longitudina	X			X			X								X											108	X		X		X	X	X	X	X	X		X	X		Change in HAMA		reduction rates (RRs) of HAMA and HAMD=[baseline -follow-up (4 wks)]/baseline	Antidepress.	PRO
Hao	2023	China	Cross-sectional	X	X														X	X									323	X				X	X		X	X	X	X								
Harris	2022	Canada	Cohort/Longitudina	X	X														X	X									144			X									X	X	X			response (≥50% MADRS reduction)	Antidepress.	Clinician assessment
Hasanzadeh	2020	Iran	Cross-sectional	X	X																			X					49	X				X		X		X	X	X								
Hasanzadeh	2019	Iran	Cohort/Longitudina	X	X																			X					46						X	X	X	X	X	X	X	X	X			response (HRSD and BDI)	rTMS	Clinician assessment, PRO
He	2019	China	Cross-sectional		X	X													X	X			X						84	X				X	X	X		X	X	X								
He	2022	China	Cohort/Longitudina	X	X														X	X									100	X				X		X		X	X	X								
Hellewell	2019	UK	Cohort/Longitudinal		X													X											295	X		X		X	X	X		X	X	X								
Hong	2022	China	Cross-sectional	X	X													X											NR	X				X	X			X	X	X								
Hopman	2021	China	Cohort/Longitudina	X	X														X	X									70		X										X	X	X			response (≥50% MADRS reduction)	rTMS	Clinician assessment
Hou	2021	China	Cross-sectional	X	X														X	X									119	X		X	X	X	X			X	X	X								
Hou	2018	China	Cross-sectional		X														X	X									119	X		X	X	X	X	X		X	X									
Hou	2018	China	Cohort/Longitudinal		X														X	X									124	X			X								X	X				response (≥50% HAMD reduction)	Antidepress.	Clinician assessment

Hou	2016	China	Cohort/Longitudinal		X														X	X									132	X			X		X	X					X	X				response (>50% HAMD reduction)	Antidepress.	Clinician assessment
Hu	2019	China	Cohort/Longitudinal		X													X											93	X		X		X	X	X		X	X									
Hu	2019	China	Cross-sectional								X								X	X									176	X		X		X	X	X		X	X									
Hu	2016	China	Cohort/Longitudinal								X							X											66	X				X	X	X	X	X	X	X								
Hu	2023	China	Cross-sectional	X	X													X											288					X	X	X		X	X	X								
Huang	2022	Taiwan	Cross-sectional	X	X	X																			X			X	NR	X				X	X			X	X	X								
Huang	2023	China	Cohort/Longitudinal								X								X	X									50												X	X	X			response (>50% Y-BOCS reduction)	Psychotherapy	PRO
Ichikawa	2020	Japan	Cohort/Longitudina	X	X														X	X									130	X				X	X	X	X	X	X	X								
Im	2017	South Korea	Cohort/Longitudina	X								X						X											59	X				X	X	X			X									
Isserles	2018	US	RCT		X																			X					190			X									X	X				response (≥50% HRSD reduction)	rTMS	Clinician assessment
James	2022	US	Cross-sectional									X													X			X	432	X				X		X		X	X	X								
Jiang	2023	Korea	Cross-sectional	X	X																			X					62	X		X		X		X	X	X	X	X								
Januszko	2021	Poland	Cohort/Longitudinal										X	X										X					34			X									X				abstinence after treatment			
Jaworska	2018	Canada	RCT	X	X																			X					51			X									X	X	X			response (MADRS)	Antidepress.	Clinician assessment
Jaworska	2014	Canada	Cohort/Longitudinal		X																			X					51			X									X	X				response (HAMD or MADRS)	Antidepress.	Clinician assessment
Jaworska	2013	Canada	Cohort/Longitudinal		X																			X					51			X									X	X				response (50% MADRS reduction)	Antidepress.	Clinician assessment
Jiang	2021	China	Cross-sectional	X	X																			X					30	X				X		X			X	X								
Jiang	2020	China	Cross-sectional	X	X																				X			X	84	X		X		X	X	X		X	X	X								
Jiang	2018	Multiple countries	Cohort/Longitudinal		X													X											82	X											X	X	X			response (≥50% HDRS reduction) or remission (≤7 HDRS)	ECT	Clinician assessment
Jiang	2023	China	Cross-sectional	X			X	X										X											78	X		X		X	X		X	X	X	X								
Jing	2022	China	Cross-sectional	X	X														X	X									858	X		X		X		X	X	X	X	X								
Ju	2022	China	Cohort/Longitudina	X	X													X											307	X		X									X	X	X		separate stable remission (HAMD<8) and recurrence (SCID) at 2 yrs (among those who were in remission at 6 mo)		Antidepress.	Clinician assessment + interview
Kamarajan	2020	US	Cross-sectional	X									X	X										X					60	X				X	X			X	X									
Kamarajan	2022	US	Cross-sectional										X	X					X	X									60					X	X				X	X								
Kang	2023	China	Cross-sectional	X	X																			X					100	X		X		X	X	X			X	X								
Karim	2018	US	Cohort/Longitudina	X	X														X	X	X								49												X	X	X			remission (MADRS=10 for ≥2 weeks)	Antidepress.	Clinician assessment
Kaufman	2015	US	Cross-sectional		X													X							X	X			107	X				X	X	X	X		X									
Khadidos	2023	Malaysia	Cross-sectional	X	X																			X					64	X				X	X			X	X	X								
Kim	2020	South Korea	Cross-sectional									X												X					81	X				X	X		X	X	X									
Kim	2022	Korea	Cross-sectional	X			X		X									X											116					X			X	X	X	X								
Kim	2022	Korea	Cohort/Longitudina	X								X												X					48												X	X				response (>50% CAPS-5 reduction)	tDCS	Clinician assessment
Kinreich	2021	US	Cohort/Longitudina	X									X	X										X					1376												X	X			remission (absence of all AUD criteria other than craving for ≥12 months and either low-risk drinking or abstinence)			
Kipli	2015	US	Cross-sectional	X	X													X											115	X				X	X		X	X	X	X								
Klumpp	2020	US	Cohort/Longitudinal		X		X	X		X		X							X		X								135	X		X									X					response (>50% HAMA/HAMD reduction)	psychotherapy	Clinician assessment
Klumpp	2017	US	Cohort/Longitudinal				X		X										X		X								34			X									X	X	X			response (≥50% LSAS reduction)	psychotherapy	PRO
Koller-Schlaud	2020	Germany	Cross-sectional		X																			X					87	X				X		X		X	X	X								
Kondo	2023	Canada	Cross-sectional	X		X													X		X								36	X				X	X	X	X	X	X	X								
Kong	2023	China	Cross-sectional	X	X													X											187	X				X	X			X	X	X								
Koo	2019	Germany	Cross-sectional		X													X						X					40	X				X	X			X	X									
Korgaonkar	2020	US, Australia	RCT		X														X		X								225	X		X									X					response (HRSD)	Antidepress.	Clinician assessment
Korgaonkar	2015	Australia	Cohort/Longitudinal		X													X											74												X	X	X			remission (HRSD)	Antidepress.	Clinician assessment
Korgaonkar	2014	Australia	Cohort/Longitudinal		X																	X							114	X		X									X	X	X			response (HRSD)	Antidepress.	Clinician assessment
Korgaonkar	2012	US	Cross-sectional		X																	X							46	X				X		X			X	X								
Kraus	2019	Austria	Cohort/Longitudinal		X														X		X								95	X											X	X				remission (<8 HAMD)	Antidepress.	Clinician assessment
Kwak	2020	Korea	Cross-sectional								X								X	X									217	X		X		X		X	X	X										
Lalousis	2022	NR	Cross-sectional	X	X														X	X									422	X				X	X			X	X	X								
Lanka	2020	US	Cross-sectional									X							X	X									2137					X			X		X	X								
Laxminarayan	2020	US	Cross-sectional	X								X												X					78	X				X		X	X	X	X	X								
Leaver	2018	US	Cohort/Longitudina	X	X														X	X									46		X										X		X			response (HDRS, MADRS, and QIDS)	ECT	Clinician assessment, PRO
Lebedeva	2017	Norway	Cohort/Longitudinal		X													X											69					X	X	X		X	X	X								
Lee	2011	China	Cohort/Longitudinal		X																			X					108			X									X	X				response (HDRS)	Antidepress.	Clinician assessment
Lei	2023	China	Cross-sectional	X	X	X												X											129	X				X	X	X	X	X	X									
Li	2021	China	Cross-sectional	X	X															X		X								74	X				X		X		X	X	X								
Li	2021	Taiwan	Cohort/Longitudinal		X																			X					105		X										X	X				response (≥50% HDRS reduction)	Antidepress.	Clinician assessment
Li	2021	China	Cross-sectional	X	X													X	X	X									118	X		X		X		X		X	X	X								
Li	2020	China	Cross-sectional	X	X														X	X									316	X		X		X		X		X	X	X								
Li	2020	China	Cross-sectional	X		X												X	X	X									80	X				X	X	X		X	X									
Li	2020	Taiwan	Cohort/Longitudinal		X													X											105			X									X	X				failure (<50% HDRS improvement)	TBS or rTMS	Clinician assessment
Li	2019	China	Cross-sectional	X	X																			X					48	X			X	X			X	X	X	X								
Li	2019	China	Cross-sectional	X									X			X		X					X						126	X				X	X			X	X	X								
Li	2017	China	Cross-sectional	X	X	X													X	X									66	X			X	X	X	X		X	X	X								
Li	2016	Taiwan	RCT		X													X						X	X	X			36												X	X				response (≥50% HDRS reduction)	Antidepress.	Clinician assessment
Li	2014	China	Cross-sectional	X							X											X							56	X				X	X	X	X	X										
Li	2021	China	Cross-sectional	X	X														x	x									55	X		X		X	X			X	X									
Li	2023	Taiwan	RCT	X	X																			X					96		X	X									X	X	X			response (≥50% HDRS-17 improvement)	rTMS	Clinician assessment
Li	2023	China	Cross-sectional		X																			X					75					X		X			X									
Li	2023	China	Cross-sectional	X		X												X											126					X	X		X	X	X									
Li	2022	China	Cohort/Longitudinal								X							X											58	X											X	X				response (≥35% Y-BOCS reduction)	rTMS	PRO
Li	2022	NR	Cohort/Longitudina	X								X												X					202	X				X			X	X	X	X								
Li	2023	China	Cross-sectional	X	X														X	X		X							251	X				X		X		X	X	X								
Li	2022	China	Cross-sectional	X	X																			X					92					X		X		X	X	X								
Liao	2018	China	Cross-sectional	X	X														X	X									78	X		X	X	X	X	X		X	X	X								
Lin	2023	China	Cross-sectional	X	X													X											127	X				X	X	X		X	X	X								
Lin	2022	Taiwan	Cross-sectional	X	X														X	X									432	X				X		X		X	X									
Liu	2022	China	Cross-sectional	X	X																			X					56	X		X	X	X		X		X	X	X								
Liu	2021	China	Cross-sectional	X							X								X	X									146	X		X		X	X	X	X	X	X									
Liu	2021	China	Cross-sectional								X								X	X									100	X		X		X	X	X	X	X	X									
Liu	2020	China	Cross-sectional								X							X											142	X	X	X		X	X	X	X		X									
Liu	2020	China	Cross-sectional	X	X																			X					39	X				X		X		X	X	X								
Liu	2020	US	Cohort/Longitudina	X	X														X		X								85	X		X		X	X			X	X	X								
Liu	2015	China	Cross-sectional									X							X	X									40	X		X		X		X		X	X	X								
Liu	2015	China	Cross-sectional				X	X										X											40	X		X		X	X	X		X	X	X								
Liu	2014	Taiwan	Cross-sectional		X	X												X							X			X	72	X				X	X	X			X	X								
Liu	2012	China	Cross-sectional	X	X													X											52	X	X			X	X	X		X	X	X								
Liu	2023	China	Cohort/Longitudinal		X														X	X									84	X											X	X				response (≥50% HAMD-17 or SSI-5 reduction)	Antidepress.	Clinician assessment
Liu	2023	China	Cross-sectional	X	X																			X					58	X				X	X	X	X	X	X	X								
Lord	2012	Australia	Cross-sectional	X	X														X	X									43	X			X	X	X	X		X	X									
Lu	2021	China	Cross-sectional	X		X												X											106	X				X		X		X	X	X								
Lu	2013	China	Cross-sectional	X	X																				X			X	44	X		X	X	X	X	X		X	X	X								
Lu	2023	China	Cross-sectional		X	X													X	X									248	X				X	X	X		X	X	X								
Luo	2021	China	Cross-sectional	X							X								X	X									128	X				X		X	X	X	X	X								
Lv	2021	China	Cross-sectional	X							X								X	X									78	X		X		X		X	X	X	X	X								
Lv	2022	China	Cross-sectional								X								X	X									78					X	X	X			X									
Manelis	2020	US	Cross-sectional		X	X													X		X								64	X				X	X				X									
Matsuo	2019	Japan, US	Cross-sectional	X	X													X											1565	X				X		X		X	X	X								
Matsuoka	2017	Japan	Cross-sectional		X													X											62	X				X	X				X									
McHugh	2014	US	Cohort/Longitudinal										X				X		X	X									67	X		X									X	X			relapse (first and any use of cocaine or amphetamine since discharge)			
Meijs	2022	Belgium	Cohort/Longitudinal		X																			X					334												X	X		Change in BDI-II		response (≥50% BDI-II reduction or in QIDS), 3 datasets used to evaluate predictive accuracy	Antidepress. or rTMS + psychotherapy	PRO
Meng	2020	China	Cohort/Longitudina	X	X													X											212	X											X					response (≥50% HAMD reduction) vs non-response (≤20% HAMD reduction)	Antidepress.	Clinician assessment
Meyer	2019	Italy	Cohort/Longitudinal		X													X											88	X		X									X	X	X			response (MADRS)	Antidepress.	Clinician assessment
Mishra	2020	India	Cross-sectional										X	X										X					90	X				X			X	X	X									
Modinos	2013	UK	Cross-sectional	X	X														X		X								34	X				X			X	X	X	X								
Mohammadi	2015	Canada	Cross-sectional	X	X																			X					96	X				X	X	X		X	X	X								
Moukaddam	2023	US	Cross-sectional	X	X	X	X			X		X							X		X								34					X	X				X	X								
Mourao-Miranda	2012	UK	Cross-sectional	X	X														X		X								54	X				X	X	X		X	X	X								
Mulders	2020	Multiple countries	Cohort/Longitudinal		X													X											192												X		X			response (≥50% HAMD reduction)	Antidepress.	Clinician assessment
Mumtaz	2019	Malaysia	Cross-sectional	X	X																			X					63	X				X			X	X	X	X								
Mumtaz	2018	Malaysia	Cross-sectional	X	X																			X					60	X				X			X	X	X	X								
Mumtaz	2018	Malaysia	Cross-sectional	X									X	X										X					60	X				X			X	X	X	X								
Mumtaz	2018	Malaysia	Cross-sectional	X	X																			X					64	X				X			X	X	X	X								
Mumtaz	2017	Malaysia	Cohort/Longitudina	X	X																			X					64	X											X	X	X			response (>50% BDI reduction)	Antidepress.	PRO
Mumtaz	2017	Malaysia	Cross-sectional										X	X										X					45	X				X			X	X	X	X								
Mumtaz	2017	Malaysia	Cross-sectional	X	X																			X					63	X				X	X			X	X	X								
Mwangi	2016	NR	Cross-sectional	X		X												X											256	X				X	X	X		X	X	X								
Nassibi	2022	UK	Cross-sectional	X	X																			X					84	X				X	X		X	X										
Neumeister	2013	US	Cross-sectional									X													X	X			60	X		X		X		X			X									
Nguyen	2019	US	RCT	X	X														X		X								37												X	X	X			response (HAMD)	Antidepress.	Clinician assessment
Nicholson	2019	Canada	Cross-sectional	X								X							X	X									181	X				X	X	X		X	X	X								
Niida	2018	Japan	Cross-sectional			X																X							114	X				X	X	X		X	X									
Niida	2012	Japan	Cross-sectional		X	X												X											120	X				X		X		X	X									
Niu	2022	China	Cross-sectional	X	X														X	X									142	X				X	X		X	X	X	X								
Nobakhsh	2023	Iran	Cohort/Longitudina	X	X																			X					34		X										X	X	X			response (≥50% BDI-II reduction)	rTMS	PRO
Nogovitsyn	2020	Canada	Cohort/Longitudinal		X													X											306	X		X									X					remission (MADRS)	Antidepress.	Clinician assessment
Nord	2019	UK	Cohort/Longitudinal		X														X		X								39			X									X	X	X			response (≥50% HAMD reduction)	tDCS	Clinician assessment
Oakley	2023	US	RCT	X	X																			X					228					X	X	X	X	X	X									
Olbrich	2012	Germany	Cross-sectional		X																			X					60	X		X		X		X	X	X										
Oliveira-Maia	2017	US	Cohort/Longitudinal		X																			X					51		X										X	X				response (HRDS)	rTMS	Clinician assessment
Palaniyappan	2022	China	Cross-sectional	X	X													X											141	X				X	X	X	X	X	X	X								
Pang	2020	China	Cross-sectional	X	X	X													X	X									128	X				X	X	X		X	X	X								
Pantazatos	2014	US	Cohort/Longitudina	X			X		X	X									X		X								82	X		X		X	X	X		X	X	X								
Patel	2015	US	Cohort/Longitudina	X	X														X	X		X							69					X	X	X		X	X	X	X	X	X			response (<10 HDRS)	Antidepress.	Clinician assessment
Peng	2022	US	Cohort/Longitudinal		X																			X					30												X	X	X			responders (CGI=1 or 2) vs. non-responders (CGI>2)	Antidepress.	Clinician assessment
Pillai	2019	US	Cross-sectional		X													X							X	X			41	X				X		X	X	X										
Price	2018	US	Cohort/Longitudinal				X	X											X		X								62			X									X	X	X			response (lowest quartile of the CAPS-Vigilance score)	Other	Clinician assessment
Qiao	2017	China	Cross-sectional				X	X											X	X									40	X		X		X	X	X		X	X									
Qin	2022	China, UK, US	Cross-sectional	X	X														X	X									1586	X			X	X	X	X		X	X	X								
Qin	2015	China	Cross-sectional	X	X														X	X									53	X		X		X	X	X			X	X								
Qin	2014	China	Cross-sectional	X	X													X											59	X				X	X	X		X	X	X								
Qiu	2014	China	Cross-sectional	X	X													X											64	X		X	X	X	X	X		X	X	X								
Rabinoff	2011	US	Cohort/Longitudina	X	X																			X					51			X									X	X	X			response (HAM-D)	Antidepress.	Clinician assessment
Raji	2015	US	Cross-sectional									X													X		X		196					X	X			X	X									
Rangaprakash	2018	US	Cross-sectional									X						X											87	X				X		X	X		X	X								
Rangaprakash	2017	US	Cross-sectional									X							X	X		X							87	X				X		X	X		X									
Rangaprakash	2019	US	Cross-sectional									X							X	X									87	X				X		X	X		X	X								
Ravan	2023	US	Cross-sectional	X	X																			X					142					X	X			X	X									
Redlich	2014	Germany, US	Cross-sectional	X	X	X												X											174	X				X		X		X	X	X								
Reggente	2018	US	Cohort/Longitudinal	X							X								X	X									42												X	X	X			response (YBOCS)	Psychotherapy	PRO

Rentzsch	2014	Germany	Cohort/Longitudinal		X																			X					62	X											X	X				response (≥50% HDRS reduction)	Antidepress.	Clinician assessment
Repple	2023	Germany	Cohort/Longitudina	X	X	X												X											1674					X	X			X	X	X								
Richieri	2018	France	Cohort/Longitudinal		X	X																			X		X		58		X										X	X				response	rTMS	
Richieri	2011	France	Cohort/Longitudinal		X	X																			X		X		33		X										X	X				response (≥50% BDI reduction)	rTMS	PRO
Rive	2016	Netherlands	Cross-sectional	X	X	X													X	X									81			X		X	X	X			X									
Rocha-Rego	2014	US, UK	Cross-sectional			X												X											80	X				X	X	X		X	X	X								
Rottstaedt	2018	Germany	Cross-sectional		X													X											135	X				X	X		X	X	X									
Rubin-Falcone	2018	US	Cross-sectional	X	X	X												X											52			X		X	X	X	X	X	X	X								
Ruiz-Espana	2023	Germany	Cohort/Longitudinal										X	X				X											68					X	X			X	X	X								
Sacchet	2015	US	Cross-sectional	X	X	X												X											193	X				X	X			X	X									
Sadat Shahabi	2021	Iran	Cohort/Longitudina	X	X																			X					30												X	X	X			response (BDI)	Antidepress.	PRO
Sadat Shahabi	2023	Iran	Cohort/Longitudina	X	X																			X					34		X										X	X	X			response (>50% BDI reduction)	rTMS	PRO
Sakib	2023	Bangladesh	Cross-sectional		X																			X					32	X				X			X	X	X	X								
Sankar	2016	UK	Cross-sectional	X	X													X											43	X		X		X	X	X		X	X	X								
Schmaal	2015	Netherlands	Cohort/Longitudina	X	X													X											118												X	X	X	classification of MDD course trajectories				
Schnack	2014	Netherlands	Cross-sectional	X		X												X											334	X				X	X			X	X	X								
Schnyer	2017	US	Cross-sectional	X	X																	X							50	X				X	X		X	X	X	X								
Schultz	2018	Multiple countries	Cohort/Longitudina	X	X														X	X	X								41	X											X	X	X			response (BDI and HAMD)	Antidepress. + psychotherapy	Clinician assessment, PRO
Schwartzmann	2023	Canada	Cohort/Longitudinal		X																			X					30												X	X				response (≥50% MADRS reduction)	Psychotherapy	Clinician assessment
Sekutowicz	2019	Germany	Cohort/Longitudina	X									X	X					X		X								188												X	X	X		relapse (Munich-Composite International Diagnostic Interview)			
Serpa	2014	Brazil	Cohort/Longitudina	X	X	X												X											113	X			X	X	X			X	X	X								
Shahabi	2023	Iran	Cohort/Longitudina	X	X																			X					46												X	X	X			response (≥50% BDI-II reduction)	rTMS	PRO
Shahabi	2023	Iran	Cohort/Longitudinal		X																			X					46												X	X	X			response (>50% BDI-II or HRSD reduction)	rTMS	PRO
Shalbaf	2018	Canada	Cohort/Longitudinal		X																			X					76	X	X										X	X				response (≥50% HDRS reduction)	rTMS	Clinician assessment
Shan	2020	China	Cross-sectional			X													X	X									74	X				X	X	X		X	X									
Shan	2021	China	Cross-sectional	X	X														X	X									91	X		X	X	X		X	X	X	X	X								
Shao	2019	China	Cross-sectional	X	X	X												X											130	X				X	X	X			X	X								
Shao	2023	China	Cohort/Longitudinal		X													X											237	X				X		X			X	X	X	X	X			response (≥50% HRSD reduction)	Antidepress.	Clinician assessment
Shen	2023	China	Cross-sectional		X																			X					35	X				X	X			X	X	X								
Shen	2022	China	Cross-sectional	X			X	X																X					81	X				X		X	X	X	X	X								
Sheynin	2023	US	Cohort/Longitudinal									X							X		X								98	X											X			symptom severity (change in CAPS: 1. no symptoms [no PTSD at both T1 and T3], 2. remission [PTSD at T1, but not at T3], 3. non-remission [PTSD at both T1 and T3])				Clinician assessment
Shi	2021	China	Cross-sectional	X	X													X											1022	X				X		X		X	X	X								
Shi	2018	China	Cohort/Longitudinal		X	X													X	X									102	X				X	X	X		X	X									
Shim	2019	Korea	Cross-sectional	X	X							X												X					157	X				X			X	X	X	X								
Shim	2023	Korea	Cross-sectional		X																			X					98	X				X		X			X	X								
Shim	2022	Korea	Cohort/Longitudina	X								X												X					135	X				X	X		X	X	X	X								
Shimizu	2015	Japan	Cross-sectional	X	X														X		X								62	X		X		X	X			X	X	X								
Shor	2023	Israel	Cross-sectional	X	X																			X					194	X				X	X			X	X	X								
Shu	2014	US	Cross-sectional									X												X					32					X	X	X	X	X	X									
Siegle	2012	US	Cohort/Longitudinal		X														X		X								84	X											X	X				response (50% BDI or HRSD reduction) or remission (BDI <10 or HRSD <7)	Psychotherapy	Clinician assessment, PRO
Singh	2022	India	Cohort/Longitudinal		X																			X					50			X									X					response (>50% HAM-D reduction)	Antidepress.	Clinician assessment
Song	2023	China	Cross-sectional	X	X																			X					82	X				X			X	X	X	X								
Song	2022	China	Cross-sectional	X	X														X	X									97					X	X	X		X	X									
Soni	2023	NR	Cross-sectional	X	X																			X					NR	X				X			X	X	X	X								
Squarcina	2019	Italy	Cross-sectional	X		X												X											75	X				X	X			X	X	X								
Stange	2020	US	Cross-sectional		X	X													X	X									212	X				X		X		X	X									
Stout	2021	US	RCT									X	X	X					X	X									53												X					response (CAPS)	psychotherapy	Clinician assessment
Stoyanov	2019	NR	Cross-sectional	X	X													X											30					X	X	X		X	X	X								
Stoyanov	2022	Bulgaria	Cross-sectional		X	X												X											90	X				X	X	X			X									
Strafella	2023	Canada	Cohort/Longitudinal		X																			X					122												X	X	X			response (≥50% HRSD-17 reduction) and remission (HRSD-17 <8 post-treatment and at 1-week follow-up)	rTMS	Clinician assessment
Sun	2021	China	Cross-sectional	X	X														X	X									116	X				X		X		X	X	X								
Sun	2022	China	Cross-sectional	X	X													X											241	X		X		X	X	X		X	X	X								
Sun	2022	China	Cross-sectional	X	X	X													X	X									126	X				X	X			X	X	X								
Sun	2020	Multiple Countries	Cohort/Longitudina	X	X	X												X											122												X	X	X			response (>50% HDRS reduction) or remission (<7 HDRS)	ECT	Clinician assessment
Suo	2020	China	Cohort/Longitudina	X								X							X	X									122			X		X		X		X	X									
Sverdlov	2021	Netherlands	Cross-sectional		X																			X					40	X				X	X	X		X	X	X								
Tahmasian	2017	Iran	Cross-sectional									X												X					64	X				X	X			X	X									
Takagi	2017	Japan	Cross-sectional								X								X	X									136	X				X	X		X		X	X								
Tang	2022	China	Cross-sectional	X	X	X													X	X									251	X				X	X	X		X	X	X								
Taylor	2014	US	Cohort/Longitudinal		X													X											162	X											X			Change in MADRS			Antidepress.	
Tekin Erguzel	2015	Turkey	Cross-sectional	X	X	X																		X					98					X	X	X		X	X	X								
Tenke	2011	US	Cohort/Longitudinal		X																			X					82	X		X									X					response (CGI-I "much improved" or "very much improved")	Antidepress.	Clinician assessment
Terpou	2022	Canada	Cross-sectional	X								X												X					123	X				X	X	X		X	X	X								
Tian	2020	China	Cohort/Longitudina	X	X													X											106												X	X	X			response (HRDS)	Antidepress.	Clinician assessment
Tian	2023	China	Cohort/Longitudinal		X																				X			X	100			X									X	X				response (≥50% HAMD-17 reduction)	Antidepress.	Clinician assessment
Tian	2023	China	Cross-sectional	X		X													X	X									176					X	X	X			X	X								
Tigga	2023	India	Cross-sectional	X	X																			X					92	X				X			X	X	X	X								
Tong	2023	US	Cohort/Longitudina	X	X																			X					304	X											X					response (change in HAMD17 score)	Antidepress.	Clinician assessment
Tsai	2023	Taiwan	Cohort/Longitudina	X	X																			X					113												X	X	X			response (≥50% HAMD-17 reduction) and remission (HAMD-17 ≤7 post-treatment)	Antidepress. or ECT	Clinician assessment
Tsolaki	2021	US	Cohort/Longitudinal		X	X												X											43		X										X					response (HAMD)	ECT	Clinician assessment
Uyulan	2022	Canada	Cross-sectional	X	X																			X					48	X				X	X	X		X	X	X								
Vai	2023	Italy	Cross-sectional	X		X													X		X								106	X				X		X	X	X	X	X								
van Kleef	2023	Netherlands	RCT	X	X														X	X									71	X		X									X	X	X		≥2 MDD episodes in the past 5 yrs; SCID life-chart method at 18-mo follow-up (report MDD symptomatology since baseline to specify presence of MDD episodes and time to relapse [mo] since baseline)			Clinician interview
van Rooij	2016	Netherlands	Cohort/Longitudinal									X						X											66	X											X					response (CAPS)	Psychotherapy	
van Waarde	2015	Netherlands	Cohort/Longitudina	X	X														X	X									45		X										X	X	X			remission (<10 MADRS)	ECT	Clinician assessment
Vergara	2022	US	Cohort/Longitudina	X									X	X					X	X									102	X				X			X	X	X	X								
Voineskos	2019	canada	Cross-sectional		X																			X					60	X				X		X		X	X									
Wade	2017	US	Cohort/Longitudina	X	X													X											42												X	X	X			response (HAMD)	ECT	Clinician assessment
Wade	2016	US	Cohort/Longitudina	X	X	X												X											86	X	X										X	X	X			response (≥50% HAMD reduction)	ECT	Clinician assessment
Wade	2017	US	Cohort/Longitudina	X	X													X											42		X										X	X	X			relapse (≥50% HAMD reduction and long-term follow-up HAMD ≤17)	ECT	Clinician assessment
Wang	2021	US, Brasil	Cross-sectional	X	X													X											174	X				X		X		X	X	X								
Wang	2020	China	Cross-sectional			X													X	X									41					X	X	X			X									
Wang	2019	China	Cross-sectional								X								X	X									80	X				X			X	X	X	X								
Wang	2019	China	Cohort/Longitudinal		X													X							X			X	77	X		X		X	X	X		X	X									
Wang	2017	China	Cross-sectional		X														X	X									106	X		X		X	X	X		X	X	X								
Wang	2017	China	Cross-sectional	X	X														X	X									60	X				X	X			X	X	X								
Wang	2022	China	Cohort/Longitudinal		X													X							X			X	41			X									X	X				response (>50% HRSD reduction)	Antidepress.	Clinician assessment
Wang	2022	China	Cohort/Longitudinal		X													X											87	X											X	X	X			partial response (25-50% HAMD reduction) or complete response (>50% HAMD reduction)	Antidepress.	Clinician assessment
Wang	2022	China	Cross-sectional										X	X					X	X									58					X	X			X										
Whitfield-Gabrie	2016	US	Cohort/Longitudinal				X	X										X											38			X									X	X	X			response (LSAS)	psychotherapy	PRO
Williams	2015	Australia	RCT		X														X		X								114	X		X									X	X	X			response (≥50% HRSD reduction) or remission (≤7 HRSD)	Antidepress.	Clinician assessment
Winter	2023	Germany	Cross-sectional	X	X													X	X	X		X							1801	X				X	X			X	X	X								
Wu	2020	China	Cross-sectional		X														X	X									74	X		X	X	X		X		X	X									
Wu	2021	China	Cross-sectional		X													X											152	X		X	X	X		X		X	X									
Wu	2021	Taiwan, Japan, US	Cross-sectional	X	X																			X					400	X				X			X	X	X	X								
Wu	2017	US	Cross-sectional	X		X												X											70					X	X	X		X	X	X								
Wu	2022	China	Cohort/Longitudina	X	X														X	X									67			X									X	X	X			remission (HAMD-17 ≤7 post-treatment)	Antidepress.	Clinician assessment
Xi	2022	China, CAN	Cross-sectional	X	X	X												X											146	X				X		X			X	X								
Xiao	2021	China	Cohort/Longitudina		X													X											36			X									X	X				early improvement (>20% QIDS-SR16 reduction)	Antidepress.	PRO
Xiao	2022	China	Cohort/Longitudina	X	X													X											113	X											X	X	X	Change in HAMD			Antidepress.	Clinician assessment
Xie	2022	China	Cross-sectional	X	X		X	X																X					42					X		X			X									
Xing	2020	US	Cross-sectional	X			X	X											X		X								93	X		X		X		X	X	X	X									
Xu	2022	China	Cohort/Longitudina	X	X													X											64		X										X	X	X			remission (>50% HAMD reduction and final HAMD ≤7)	ECT	Clinician assessment
Xu	2022	China	Cross-sectional		X														X	X									211	X				X	X	X			X	X	X	X	X					
Xu	2022	China	Cross-sectional	X	X														X	X									161	X		X		X		X		X	X	X								
Xue	2021	China	Cohort/Longitudina	X	X																	X							76			X									X	X	X			early improvement (≥20% HAMD reduction)	Antidepress.	Clinician assessment
Yan	2020	China	Cross-sectional	X	X														X	X									99	X				X	X	X		X	X	X								
Yan	2022	China	Cross-sectional	X	X																			X					80	X				X		X		X	X	X								
Yan	2021	China	Cross-sectional	X	X														X	X									91	X		X	X	X		X		X	X	X								
Yan	2021	China	Cohort/Longitudina	X									X			X			X	X									69	X											X	X	X			response (50% reduction)	Mixed	
Yan	2021	China	Cross-sectional	X	X													X											91	X				X		X		X	X	X								
Yan	2023	China	Cross-sectional				X	X											X	X									152	X				X			X	X	X									
Yan	2022	China	Cohort/Longitudinal								X								X	X									70			X		X	X	X	X	X	X	X								
Yan	2022	China	Cohort/Longitudinal								X							X											77	X				X	X			X	X	X								
Yan	2022	China	Cross-sectional		X														X	X									80	X				X		X		X	X									
Yang	2022	China	Cross-sectional	X							X								X	X									180	X				X			X	X	X	X								
Yang	2019	China	Cross-sectional								X								X	X									136	X		X		X	X		X	X	X	X								
Yang	2018	US	Cross-sectional	X	X													X											346	X		X		X	X	X			X	X								
Yang	2018	China	Cross-sectional	X	X														X	X									111	X		X		X		X		X	X	X								
Yang	2019	China	Cross-sectional			X												X											177	X				X		X		X	X									
Yang	2016	China	Cross-sectional		X													X											98	X				X	X	X		X	X									
Yang	2021	China	Cross-sectional	X	X	X												X											441	X				X	X	X		X	X	X								
Yang	2023	China	Cross-sectional		X																			X					67	X				X	X			X	X	X								
Yang	2023	China	Cross-sectional		X														X	X									97					X	X	X	X		X									
Yang	2023	China	Cross-sectional		X																			X					53	X				X			X	X	X	X								
Yang	2022	China	Cross-sectional		X														X	X									NR	X		X		X	X	X			X									
Yeh	2015	Taiwan	Cohort/Longitudinal		X																				X	X			78	X		X									X	X				response (≥50% HDRS reduction)	Antidepress.	
Yoshida	2017	Japan	Cross-sectional	X	X														X	X									123	X				X	X		X	X	X	X								
Yu	2018	China	Cross-sectional	X	X													X											72	X				X	X	X		X	X	X								
Yun	2015	Korea	Cohort/Longitudina	X							X							X											131	X		X									X	X	X			response (YBOCS)	Antidepress.	Clinician assessment
Zehong	2019	Taiwan	Cohort/Longitudina	X	X																			X					55		X										X	X	X			response (HDRS)	Ketamine	Clinician assessment
Zeng	2012	China	Cross-sectional	X	X														X	X									53	X		X		X		X		X	X	X								
Zhai	2021	US	Cohort/Longitudina	X									X				X	X											43												X	X	X		relapse at 30 days (CCQ-Brief)			
Zhang	2022	China	Cross-sectional		X														X	X									154	X			X	X		X		X	X									
Zhang	2022	China	Cross-sectional	X	X														X	X									95	X				X		X		X	X	X								

Zhang	2021	China	Cohort/Longitudinal		X														X	X									218	X											X	X				response (50% HAMD reduction)	Antidepress.	Clinician assessment
Zhang	2020	Canada	Cross-sectional	X								X						X							X			X	44					X	X				X									
Zhang	2016	China	Cross-sectional	X								X						X	X	X									57	X		X		X	X	X		X	X	X								
Zhang	2023	China	Cross-sectional	X	X	X												X											115					X	X	X		X	X									
Zhang	2023	China	Cross-sectional		X																			X					53	X				X			X	X	X	X								
Zhang	2022	China	Cross-sectional	X	X													X	X	X									95	X		X		X	X	X	X	X	X	X								
Zhang	2023	China	Cohort/Longitudinal		X													X											39		X										X	X				remission (MADRS ≤10 post-treatment)	Antidepress.	Clinician assessment
Zhang	2023	China	Cross-sectional	X	X																			X					48					X			X		X	X								
Zhang	2023	China	Cohort/Longitudinal		X													X											83	X											X					remission (HAMD ≤7 post-treatment)	Antidepress.	Clinician assessment
Zhang	2023	China	Cross-sectional		X													X											60	X				X	X	X			X	X								
Zhang	2023	China	Cross-sectional		X																			X					68	X				X		X	X	X	X									
Zhang	2023	China	Cross-sectional	X			X		X									X											94	X				X	X			X	X	X								
Zhang	2022	China	Cross-sectional				X		X										X	X									98	X				X	X				X	X								
Zhang	2022	China	Cross-sectional		X														X	X									96	X				X			X	X	X	X								
Zhang	2023	China	Cross-sectional	X								X						X											90					X		X	X	X	X									
Zhao	2020	China	Cross-sectional	X	X														X	X									555	X				X	X			X	X	X								
Zhao	2017	China	Cross-sectional		X	X												X											105	X				X	X	X		X										
Zhao	2022	Canada	Cross-sectional		X																			X					48	X				X			X	X	X	X								
Zhao	2022	China	Cross-sectional		X																			X					53					X			X	X	X									
Zhdanov	2020	Canada	Cohort/Longitudina	X	X																			X					122												X	X	X			response (≥50% MADRS reduction)	Antidepress.	Clinician assessment
Zheng	2019	China	Cross-sectional	X	X													X											154	X		X	X	X	X	X		X	X	X								
Zhong	2017	China	Cross-sectional	X	X														X	X									62	X		X	X	X	X			X	X	X								
Zhou	2018	China	Cross-sectional								X							X											93	X				X	X		X	X	X									
Zhu	2021	China	Cross-sectional	X								X							X	X									217	X		X		X	X	X		X	X									
Zhu	2021	China	Cross-sectional	X	X														X	X									61	X		X	X	X	X			X	X	X								
Zhu	2020	China	Cross-sectional									X						X											116	X		X		X	X	X		X	X									
Zhu	2018	US	Cross-sectional	X									X	X					X	X									92	X		X		X	X			X	X									
Zhu	2018	China	Cohort/Longitudinal		X														X	X									47												X	X				response (≥50% HRSD reduction)	Antidepress.	Clinician assessment
Zhu	2019	China	Cross-sectional	X	X														X	X									74	X				X			X	X	X	X								
Zhu	2023	US	Cohort/Longitudina	X	X														X	X									1601					X	X	X	X	X	X									
Zhutovsky	2019	Netherlands	Cohort/Longitudinal									X						X											72	X											X	X	X			response (≥30% CAPS reduction)	Psychotherapy	Clinician assessment

References

1. Ullman K, Landsteiner A, Anthony M, et al. Neuroimaging and Neurophysiologic Biomarkers for Mental Health: An Evidence Map. Washington, DC: Evidence Synthesis Program, Health Services Research and Development Service, Office of Research and Development, Department of Veterans Affairs. VA ESP Project #09-009; 2022.
2. Runia N, Yucel DE, Lok A, et al. The neurobiology of treatment-resistant depression: A systematic review of neuroimaging studies. *Neurosci Biobehav Rev*. Jan 2022;132:433-448. doi:10.1016/j.neubiorev.2021.12.008
3. Bruun CF, Arnbjerg CJ, Kessing LV. Electroencephalographic Parameters Differentiating Melancholic Depression, Non-melancholic Depression, and Healthy Controls. A Systematic Review. *Front Psychiatry*. 2021;12:648713. doi:10.3389/fpsyt.2021.648713
4. Cohen SE, Zantvoord JB, Wezenberg BN, Bockting CLH, van Wingen GA. Magnetic resonance imaging for individual prediction of treatment response in major depressive disorder: a systematic review and meta-analysis. *Transl Psychiatry*. Mar 15 2021;11(1):168. doi:10.1038/s41398-021-01286-x
5. De Crescenzo F, Ciliberto M, Menghini D, Treglia G, Ebmeier KP, Janiri L. Is (18)F-FDG-PET suitable to predict clinical response to the treatment of geriatric depression? A systematic review of PET studies. *Aging Ment Health*. Sep 2017;21(9):889-894. doi:10.1080/13607863.2016.1247413
6. Dichter GS, Gibbs D, Smoski MJ. A systematic review of relations between resting-state functional-MRI and treatment response in major depressive disorder. *J Affect Disord*. Feb 1 2015;172:8-17. doi:10.1016/j.jad.2014.09.028
7. Enneking V, Leehr EJ, Dannlowski U, Redlich R. Brain structural effects of treatments for depression and biomarkers of response: a systematic review of neuroimaging studies. *Psychol Med*. Jan 2020;50(2):187-209. doi:10.1017/S0033291719003660
8. Fu CH, Steiner H, Costafreda SG. Predictive neural biomarkers of clinical response in depression: a meta-analysis of functional and structural neuroimaging studies of pharmacological and psychological therapies. *Neurobiol Dis*. Apr 2013;52:75-83. doi:10.1016/j.nbd.2012.05.008
9. Gillett G, Tomlinson A, Efthimiou O, Cipriani A. Predicting treatment effects in unipolar depression: A meta-review. *Pharmacol Ther*. Aug 2020;212:107557. doi:10.1016/j.pharmthera.2020.107557
10. Khosla A, Khandnor P, Chand T. Automated diagnosis of depression from EEG signals using traditional and deep learning approaches: A comparative analysis. *Biocybernetics and Biomedical Engineering*. 2022;42(1):108-142. doi:10.1016/j.bbe.2021.12.005
11. Levy A, Taib S, Arbus C, et al. Neuroimaging Biomarkers at Baseline Predict Electroconvulsive Therapy Overall Clinical Response in Depression: A Systematic Review. *J ECT*. Jun 2019;35(2):77-83. doi:10.1097/YCT.0000000000000570
12. Long Z, Du L, Zhao J, Wu S, Zheng Q, Lei X. Prediction on treatment improvement in depression with resting state connectivity: A coordinate-based meta-analysis. *J Affect Disord*. Nov 1 2020;276:62-68. doi:10.1016/j.jad.2020.06.072
13. Masse-Sibille C, Djamila B, Julie G, Emmanuel H, Pierre V, Gilles C. Predictors of Response and Remission to Antidepressants in Geriatric Depression: A Systematic Review. *J Geriatr Psychiatry Neurol*. Nov 2018;31(6):283-302. doi:10.1177/0891988718807099
14. Scheepens DS, van Waarde JA, Lok A, de Vries G, Denys D, van Wingen GA. The Link Between Structural and Functional Brain Abnormalities in Depression: A Systematic Review of

Multimodal Neuroimaging Studies. *Front Psychiatry*. 2020;11:485. doi:10.3389/fpsyt.2020.00485
15. Siegel-Ramsay JE, Bertocci MA, Wu B, Phillips ML, Strakowski SM, Almeida JRC. Distinguishing between depression in bipolar disorder and unipolar depression using magnetic resonance imaging: a systematic review. *Bipolar Disord*. Jan 20 2022;doi:10.1111/bdi.13176
16. Simon L, Blay M, Galvao F, Brunelin J. Using EEG to Predict Clinical Response to Electroconvulsive Therapy in Patients With Major Depression: A Comprehensive Review. *Front Psychiatry*. 2021;12:643710. doi:10.3389/fpsyt.2021.643710
17. Sinha P, Joshi H, Ithal D. Resting State Functional Connectivity of Brain With Electroconvulsive Therapy in Depression: Meta-Analysis to Understand Its Mechanisms. *Front Hum Neurosci*. 2020;14:616054. doi:10.3389/fnhum.2020.616054
18. van der Vinne N, Vollebregt MA, van Putten M, Arns M. Frontal alpha asymmetry as a diagnostic marker in depression: Fact or fiction? A meta-analysis. *Neuroimage Clin*. 2017;16:79-87. doi:10.1016/j.nicl.2017.07.006
19. Widge AS, Bilge MT, Montana R, et al. Electroencephalographic Biomarkers for Treatment Response Prediction in Major Depressive Illness: A Meta-Analysis. *Am J Psychiatry*. Jan 1 2019;176(1):44-56. doi:10.1176/appi.ajp.2018.17121358
20. Hozer F, Houenou J. Can neuroimaging disentangle bipolar disorder? *J Affect Disord*. May 2016;195:199-214. doi:10.1016/j.jad.2016.01.039
21. Librenza-Garcia D, Kotzian BJ, Yang J, et al. The impact of machine learning techniques in the study of bipolar disorder: A systematic review. *Neurosci Biobehav Rev*. Sep 2017;80:538-554. doi:10.1016/j.neubiorev.2017.07.004
22. Seeberg I, Kjaerstad HL, Miskowiak KW. Neural and Behavioral Predictors of Treatment Efficacy on Mood Symptoms and Cognition in Mood Disorders: A Systematic Review. *Front Psychiatry*. 2018;9(JUL):337. doi:10.3389/fpsyt.2018.00337
23. Whalley HC, Papmeyer M, Sprooten E, Lawrie SM, Sussmann JE, McIntosh AM. Review of functional magnetic resonance imaging studies comparing bipolar disorder and schizophrenia. *Bipolar Disord*. Jun 2012;14(4):411-31. doi:10.1111/j.1399-5618.2012.01016.x
24. Colvonen PJ, Glassman LH, Crocker LD, et al. Pretreatment biomarkers predicting PTSD psychotherapy outcomes: A systematic review. *Neurosci Biobehav Rev*. Apr 2017;75:140-156. doi:10.1016/j.neubiorev.2017.01.027
25. Nelson MD, Tumpap AM. Posttraumatic stress disorder symptom severity is associated with left hippocampal volume reduction: a meta-analytic study. *CNS Spectr*. Aug 2017;22(4):363-372. doi:10.1017/S1092852916000833
26. Fullana MA, Abramovitch A, Via E, et al. Diagnostic biomarkers for obsessive-compulsive disorder: A reasonable quest or ignis fatuus? *Neurosci Biobehav Rev*. Nov 2020;118:504-513. doi:10.1016/j.neubiorev.2020.08.008
27. Qing X, Gu L, Li D. Abnormalities of Localized Connectivity in Obsessive-Compulsive Disorder: A Voxel-Wise Meta-Analysis. *Front Hum Neurosci*. 2021;15:739175. doi:10.3389/fnhum.2021.739175
28. Santos VA, Carvalho DD, Van Ameringen M, Nardi AE, Freire RC. Neuroimaging findings as predictors of treatment outcome of psychotherapy in anxiety disorders. *Prog Neuropsychopharmacol Biol Psychiatry*. Apr 20 2019;91:60-71. doi:10.1016/j.pnpbp.2018.04.001

29. Xu J, Van Dam NT, Feng C, et al. Anxious brain networks: A coordinate-based activation likelihood estimation meta-analysis of resting-state functional connectivity studies in anxiety. *Neurosci Biobehav Rev*. Jan 2019;96:21-30. doi:10.1016/j.neubiorev.2018.11.005
30. Achalia R, Sinha A, Jacob A, et al. A proof of concept machine learning analysis using multimodal neuroimaging and neurocognitive measures as predictive biomarker in bipolar disorder. *Asian J Psychiatr*. Apr 2020;50:101984. doi:10.1016/j.ajp.2020.101984
31. Adinoff B, Gu H, Merrick C, et al. Basal Hippocampal Activity and Its Functional Connectivity Predicts Cocaine Relapse. *Biol Psychiatry*. Oct 1 2015;78(7):496-504. doi:10.1016/j.biopsych.2014.12.027
32. Almeida JR, Mourao-Miranda J, Aizenstein HJ, et al. Pattern recognition analysis of anterior cingulate cortex blood flow to classify depression polarity. *Br J Psychiatry*. Sep 2013;203(3):310-1. doi:10.1192/bjp.bp.112.122838
33. Altuglu TB, Metin B, Tulay EE, et al. Prediction of treatment resistance in obsessive compulsive disorder patients based on EEG complexity as a biomarker. *Clin Neurophysiol*. Mar 2020;131(3):716-724. doi:10.1016/j.clinph.2019.11.063
34. Ambrosi E, Arciniegas DB, Madan A, et al. Insula and amygdala resting-state functional connectivity differentiate bipolar from unipolar depression. *Acta Psychiatr Scand*. Jul 2017;136(1):129-139. doi:10.1111/acps.12724
35. Amen DG, Krishnamani P, Meysami S, Newberg A, Raji CA. Classification of Depression, Cognitive Disorders, and Co-Morbid Depression and Cognitive Disorders with Perfusion SPECT Neuroimaging. *J Alzheimers Dis*. 2017;57(1):253-266. doi:10.3233/JAD-161232
36. Amen DG, Raji CA, Willeumier K, et al. Functional Neuroimaging Distinguishes Posttraumatic Stress Disorder from Traumatic Brain Injury in Focused and Large Community Datasets. *PLoS One*. 2015;10(7):e0129659. doi:10.1371/journal.pone.0129659
37. Arns M, Cerquera A, Gutierrez RM, Hasselman F, Freund JA. Non-linear EEG analyses predict non-response to rTMS treatment in major depressive disorder. *Clin Neurophysiol*. Jul 2014;125(7):1392-9. doi:10.1016/j.clinph.2013.11.022
38. Arns M, Drinkenburg WH, Fitzgerald PB, Kenemans JL. Neurophysiological predictors of non-response to rTMS in depression. *Brain Stimul*. Oct 2012;5(4):569-76. doi:10.1016/j.brs.2011.12.003
39. Arribas JI, Calhoun VD, Adali T. Automatic Bayesian classification of healthy controls, bipolar disorder, and schizophrenia using intrinsic connectivity maps from FMRI data. *IEEE Trans Biomed Eng*. Dec 2010;57(12):2850-60. doi:10.1109/TBME.2010.2080679
40. Bachmann M, Lass J, Hinrikus H. Single channel EEG analysis for detection of depression. *Biomedical Signal Processing and Control*. 2017;31:391-397. doi:10.1016/j.bspc.2016.09.010
41. Bailey NW, Hoy KE, Rogasch NC, et al. Responders to rTMS for depression show increased fronto-midline theta and theta connectivity compared to non-responders. *Brain Stimul*. Jan - Feb 2018;11(1):190-203. doi:10.1016/j.brs.2017.10.015
42. Baranger D, Halchenko Y, Satz S, et al. Aberrant Levels of Cortical Myelin Distinguish Individuals With Unipolar Depression From Healthy Controls. *Biological Psychiatry*. 2021;89(9):S364. 2021 Annual Scientific Convention and Meeting. Virtual, Online. doi:10.1016/j.biopsych.2021.02.907

43. Bares M, Novak T, Vlcek P, Hejzlar M, Brunovsky M. Early change of prefrontal theta cordance and occipital alpha asymmetry in the prediction of responses to antidepressants. *Int J Psychophysiol*. Sep 2019;143:1-8. doi:10.1016/j.ijpsycho.2019.06.006
44. Bares M, Novak T, Brunovsky M, Kopecek M, Hoschl C. The Comparison of Effectiveness of Various Potential Predictors of Response to Treatment With SSRIs in Patients With Depressive Disorder. *J Nerv Ment Dis*. Aug 2017;205(8):618-626. doi:10.1097/NMD.0000000000000574
45. Bares M, Novak T, Kopecek M, Brunovsky M, Stopkova P, Hoschl C. The effectiveness of prefrontal theta cordance and early reduction of depressive symptoms in the prediction of antidepressant treatment outcome in patients with resistant depression: analysis of naturalistic data. *Eur Arch Psychiatry Clin Neurosci*. Feb 2015;265(1):73-82. doi:10.1007/s00406-014-0506-8
46. Bartlett EA, DeLorenzo C, Sharma P, et al. Pretreatment and early-treatment cortical thickness is associated with SSRI treatment response in major depressive disorder. *Neuropsychopharmacology*. Oct 2018;43(11):2221-2230. doi:10.1038/s41386-018-0122-9
47. Baskaran A, Farzan F, Milev R, et al. The comparative effectiveness of electroencephalographic indices in predicting response to escitalopram therapy in depression: A pilot study. *J Affect Disord*. Feb 2018;227:542-549. doi:10.1016/j.jad.2017.10.028
48. Bi K, Luo G, Tian S, et al. An enriched granger causal model allowing variable static anatomical constraints. *Neuroimage Clin*. 2019;21:101592. doi:10.1016/j.nicl.2018.11.002
49. Bi K, Chattun MR, Liu X, et al. Abnormal early dynamic individual patterns of functional networks in low gamma band for depression recognition. *J Affect Disord*. Oct 1 2018;238:366-374. doi:10.1016/j.jad.2018.05.078
50. Bi K, Hua L, Wei M, Qin J, Lu Q, Yao Z. Dynamic functional-structural coupling within acute functional state change phases: Evidence from a depression recognition study. *J Affect Disord*. Feb 2016;191:145-55. doi:10.1016/j.jad.2015.11.041
51. Brandt IM, Kohler-Forsberg K, Ganz M, et al. Reward processing in major depressive disorder and prediction of treatment response - Neuropharm study. *Eur Neuropsychopharmacol*. Mar 2021;44:23-33. doi:10.1016/j.euroneuro.2020.12.010
52. Braund TA, Breukelaar IA, Griffiths K, et al. Intrinsic Functional Connectomes Characterize Neuroticism in Major Depressive Disorder and Predict Antidepressant Treatment Outcomes. *Biol Psychiatry Cogn Neurosci Neuroimaging*. Mar 2022;7(3):276-284. doi:10.1016/j.bpsc.2021.07.010
53. Bruin WB, Oltedal L, Bartsch H, et al. Development and validation of a multimodal neuroimaging biomarker for electroconvulsive therapy outcome in depression: a multicenter machine learning analysis. *medRxiv*. 2022;doi:10.1101/2021.07.29.21261206
54. Burger C, Redlich R, Grotegerd D, et al. Differential Abnormal Pattern of Anterior Cingulate Gyrus Activation in Unipolar and Bipolar Depression: an fMRI and Pattern Classification Approach. *Neuropsychopharmacology*. Jun 2017;42(7):1399-1408. doi:10.1038/npp.2017.36
55. Camchong J, Haynos AF, Hendrickson T, et al. Resting Hypoconnectivity of Theoretically Defined Addiction Networks during Early Abstinence Predicts Subsequent Relapse in Alcohol Use Disorder. *Cereb Cortex*. Jun 7 2022;32(12):2688-2702. doi:10.1093/cercor/bhab374

56. Cash RFH, Cocchi L, Anderson R, et al. A multivariate neuroimaging biomarker of individual outcome to transcranial magnetic stimulation in depression. *Hum Brain Mapp*. Nov 1 2019;40(16):4618-4629. doi:10.1002/hbm.24725
57. Chen Q, Bi Y, Zhao X, et al. Regional amplitude abnormities in the major depressive disorder: A resting-state fMRI study and support vector machine analysis. *J Affect Disord*. Jul 1 2022;308:1-9. doi:10.1016/j.jad.2022.03.079
58. Chen Y, Ou Y, Lv D, et al. Decreased Nucleus Accumbens Connectivity at Rest in Medication-Free Patients with Obsessive-Compulsive Disorder. *Neural Plast*. 2021;2021:9966378. doi:10.1155/2021/9966378
59. Chen ST, Ku LC, Chen SJ, Shen TW. The Changes of qEEG Approximate Entropy during Test of Variables of Attention as a Predictor of Major Depressive Disorder. *Brain Sci*. Nov 7 2020;10(11)doi:10.3390/brainsci10110828
60. Chen VC, Wong FT, Tsai YH, et al. Convolutional Neural Network-Based Deep Learning Model for Predicting Differential Suicidality in Depressive Patients Using Brain Generalized q-Sampling Imaging. *J Clin Psychiatry*. Feb 23 2021;82(2)doi:10.4088/JCP.19m13225
61. Cheng Y, Xu J, Arnone D, et al. Resting-state brain alteration after a single dose of SSRI administration predicts 8-week remission of patients with major depressive disorder. *Psychol Med*. Feb 2017;47(3):438-450. doi:10.1017/S0033291716002440
62. Chin Fatt CR, Jha MK, Cooper CM, et al. Effect of Intrinsic Patterns of Functional Brain Connectivity in Moderating Antidepressant Treatment Response in Major Depression. *Am J Psychiatry*. Feb 1 2020;177(2):143-154. doi:10.1176/appi.ajp.2019.18070870
63. Colle R, Chupin M, Cury C, et al. Depressed suicide attempters have smaller hippocampus than depressed patients without suicide attempts. *J Psychiatr Res*. Feb 2015;61:13-8. doi:10.1016/j.jpsychires.2014.12.010
64. Cook IA, Hunter AM, Caudill MM, Abrams MJ, Leuchter AF. Prospective testing of a neurophysiologic biomarker for treatment decisions in major depressive disorder: The PRISE-MD trial. *J Psychiatr Res*. May 2020;124:159-165. doi:10.1016/j.jpsychires.2020.02.028
65. Cook IA, Hunter AM, Gilmer WS, et al. Quantitative electroencephalogram biomarkers for predicting likelihood and speed of achieving sustained remission in major depression: a report from the biomarkers for rapid identification of treatment effectiveness in major depression (BRITE-MD) trial. *J Clin Psychiatry*. Jan 2013;74(1):51-6. doi:10.4088/JCP.10m06813
66. Costafreda SG, Fu CH, Picchioni M, et al. Pattern of neural responses to verbal fluency shows diagnostic specificity for schizophrenia and bipolar disorder. *BMC Psychiatry*. Jan 28 2011;11:18. doi:10.1186/1471-244X-11-18
67. Crane NA, Jenkins LM, Bhaumik R, et al. Multidimensional prediction of treatment response to antidepressants with cognitive control and functional MRI. *Brain*. Feb 2017;140(2):472-486. doi:10.1093/brain/aww326
68. Crowther A, Smoski MJ, Minkel J, et al. Resting-state connectivity predictors of response to psychotherapy in major depressive disorder. *Neuropsychopharmacology*. Jun 2015;40(7):1659-73. doi:10.1038/npp.2015.12
69. Cui G, Ou Y, Chen Y, et al. Altered Global Brain Functional Connectivity in Drug-Naive Patients With Obsessive-Compulsive Disorder. *Front Psychiatry*. 2020;11:98. doi:10.3389/fpsyt.2020.00098

70. Dai X, Gao L, Zhang H, Wei X, Liu Z. A combination of support vector machine and voxel-based morphometry in adult male alcohol use disorder patients with cognitive deficits. *Brain Res*. Nov 15 2021;1771:147644. doi:10.1016/j.brainres.2021.147644
71. de la Salle S, Jaworska N, Blier P, Smith D, Knott V. Using prefrontal and midline right frontal EEG-derived theta cordance and depressive symptoms to predict the differential response or remission to antidepressant treatment in major depressive disorder. *Psychiatry Res Neuroimaging*. Aug 30 2020;302:111109. doi:10.1016/j.pscychresns.2020.111109
72. Deng F, Wang Y, Huang H, et al. Abnormal segments of right uncinate fasciculus and left anterior thalamic radiation in major and bipolar depression. *Prog Neuropsychopharmacol Biol Psychiatry*. Feb 2 2018;81:340-349. doi:10.1016/j.pnpbp.2017.09.006
73. Ding X, Yue X, Zheng R, Bi C, Li D, Yao G. Classifying major depression patients and healthy controls using EEG, eye tracking and galvanic skin response data. *J Affect Disord*. May 15 2019;251:156-161. doi:10.1016/j.jad.2019.03.058
74. Drysdale AT, Grosenick L, Downar J, et al. Resting-state connectivity biomarkers define neurophysiological subtypes of depression. *Nat Med*. Jan 2017;23(1):28-38. Comment in: Nat Med. 2017 Jan 6;23 (1):16-17 PMID: 28060802 [https://www.ncbi.nlm.nih.gov/pubmed/28060802] Erratum in: Nat Med. 2017 Feb 7;23 (2):264 PMID: 28170383 [https://www.ncbi.nlm.nih.gov/pubmed/28170383] Comment in: Neurosci Bull. 2017 Jun;33(3):351-353 PMID: 28224286 [https://www.ncbi.nlm.nih.gov/pubmed/28224286] Comment in: Nature. 2017 Jun 13;546(7658):339 PMID: 28617481 [https://www.ncbi.nlm.nih.gov/pubmed/28617481]. doi:10.1038/nm.4246
75. Duan L, Duan H, Qiao Y, et al. Machine Learning Approaches for MDD Detection and Emotion Decoding Using EEG Signals. *Front Hum Neurosci*. 2020;14:284. doi:10.3389/fnhum.2020.00284
76. Dunlop BW, Rajendra JK, Craighead WE, et al. Functional Connectivity of the Subcallosal Cingulate Cortex And Differential Outcomes to Treatment With Cognitive-Behavioral Therapy or Antidepressant Medication for Major Depressive Disorder. *Am J Psychiatry*. Jun 1 2017;174(6):533-545. Erratum in: Am J Psychiatry. 2017 Jun 1;174(6):604 PMID: 28565951 [https://www.ncbi.nlm.nih.gov/pubmed/28565951] Comment in: Am J Psychiatry. 2017 Jun 1;174(6):503-505 PMID: 28565957 [https://www.ncbi.nlm.nih.gov/pubmed/28565957]. doi:10.1176/appi.ajp.2016.16050518
77. Durazzo TC, Meyerhoff DJ. Psychiatric, Demographic, and Brain Morphological Predictors of Relapse After Treatment for an Alcohol Use Disorder. *Alcohol Clin Exp Res*. Jan 2017;41(1):107-116. doi:10.1111/acer.13267
78. Ellard KK, Zimmerman JP, Kaur N, et al. Functional Connectivity Between Anterior Insula and Key Nodes of Frontoparietal Executive Control and Salience Networks Distinguish Bipolar Depression From Unipolar Depression and Healthy Control Subjects. *Biol Psychiatry Cogn Neurosci Neuroimaging*. May 2018;3(5):473-484. Comment in: Biol Psychiatry Cogn Neurosci Neuroimaging. 2018 May;3(5):411-413 PMID: 29735151 [https://www.ncbi.nlm.nih.gov/pubmed/29735151]. doi:10.1016/j.bpsc.2018.01.013
79. Erguzel TT, Uyulan C, Unsalver B, et al. Entropy: A Promising EEG Biomarker Dichotomizing Subjects With Opioid Use Disorder and Healthy Controls. *Clin EEG Neurosci*. Nov 2020;51(6):373-381. doi:10.1177/1550059420905724
80. Erguzel TT, Noyan CO, Eryilmaz G, et al. Binomial Logistic Regression and Artificial Neural Network Methods to Classify Opioid-Dependent Subjects and Control Group Using

Quantitative EEG Power Measures. *Clin EEG Neurosci*. Sep 2019;50(5):303-310. doi:10.1177/1550059418824450
81. Erguzel TT, Ozekes S, Gultekin S, Tarhan N, Hizli Sayar G, Bayram A. Neural Network Based Response Prediction of rTMS in Major Depressive Disorder Using QEEG Cordance. *Psychiatry Investig*. Jan 2015;12(1):61-5. doi:10.4306/pi.2015.12.1.61
82. Erguzel TT, Ozekes S, Gultekin S, Tarhan N. Ant Colony Optimization Based Feature Selection Method for QEEG Data Classification. *Psychiatry Investig*. Jul 2014;11(3):243-50. doi:10.4306/pi.2014.11.3.243
83. Etkin A, Maron-Katz A, Wu W, et al. Using fMRI connectivity to define a treatment-resistant form of post-traumatic stress disorder. *Sci Transl Med*. Apr 3 2019;11(486)Comment in: Nat Hum Behav. 2019 Jun;3(6):549 PMID: 31097819 [https://www.ncbi.nlm.nih.gov/pubmed/31097819]. doi:10.1126/scitranslmed.aal3236
84. Fan X, Huang X, Zhao Y, Wang L, Yu H, Zhao G. Predicting Prognostic Effects of Acupuncture for Depression Using the Electroencephalogram. *Evid Based Complement Alternat Med*. 2022;2022:1381683. doi:10.1155/2022/1381683
85. Fang P, Zeng LL, Shen H, et al. Increased cortical-limbic anatomical network connectivity in major depression revealed by diffusion tensor imaging. *PLoS One*. 2012;7(9):e45972. doi:10.1371/journal.pone.0045972
86. Farb NAS, Desormeau P, Anderson AK, Segal ZV. Static and treatment-responsive brain biomarkers of depression relapse vulnerability following prophylactic psychotherapy: Evidence from a randomized control trial. *Neuroimage Clin*. 2022;34:102969. doi:10.1016/j.nicl.2022.102969
87. Feder S, Sundermann B, Wersching H, et al. Sample heterogeneity in unipolar depression as assessed by functional connectivity analyses is dominated by general disease effects. *J Affect Disord*. Nov 2017;222:79-87. doi:10.1016/j.jad.2017.06.055
88. Fonzo GA, Goodkind MS, Oathes DJ, et al. PTSD Psychotherapy Outcome Predicted by Brain Activation During Emotional Reactivity and Regulation. *Am J Psychiatry*. Dec 1 2017;174(12):1163-1174. Comment in: Am J Psychiatry. 2017 Dec 1;174(12):1131-1133 PMID: 29191039 [https://www.ncbi.nlm.nih.gov/pubmed/29191039]. doi:10.1176/appi.ajp.2017.16091072
89. Frangou S, Dima D, Jogia J. Towards person-centered neuroimaging markers for resilience and vulnerability in Bipolar Disorder. *Neuroimage*. Jan 15 2017;145(Pt B):230-237. doi:10.1016/j.neuroimage.2016.08.066
90. Frick A, Engman J, Alaie I, et al. Neuroimaging, genetic, clinical, and demographic predictors of treatment response in patients with social anxiety disorder. *J Affect Disord*. Jan 15 2020;261:230-237. doi:10.1016/j.jad.2019.10.027
91. Gao Y, Wang X, Xiong Z, et al. Abnormal Fractional Amplitude of Low-Frequency Fluctuation as a Potential Imaging Biomarker for First-Episode Major Depressive Disorder: A Resting-State fMRI Study and Support Vector Machine Analysis. *Front Neurol*. 2021;12:751400. doi:10.3389/fneur.2021.751400
92. Gao C, Xu Z, Tan T, et al. Combination of spontaneous regional brain activity and HTR1A/1B DNA methylation to predict early responses to antidepressant treatments in MDD. *J Affect Disord*. Apr 1 2022;302:249-257. doi:10.1016/j.jad.2022.01.098
93. Gartner M, Ghisu ME, Scheidegger M, et al. Aberrant working memory processing in major depression: evidence from multivoxel pattern classification. *Neuropsychopharmacology*. Aug 2018;43(9):1972-1979. doi:10.1038/s41386-018-0081-1

94. Ge R, Downar J, Blumberger DM, Daskalakis ZJ, Vila-Rodriguez F. Functional connectivity of the anterior cingulate cortex predicts treatment outcome for rTMS in treatment-resistant depression at 3-month follow-up. *Brain Stimul*. Jan - Feb 2020;13(1):206-214. doi:10.1016/j.brs.2019.10.012
95. Ge R, Downar J, Blumberger DM, Daskalakis ZJ, Lam RW, Vila-Rodriguez F. Structural network integrity of the central executive network is associated with the therapeutic effect of rTMS in treatment resistant depression. *Prog Neuropsychopharmacol Biol Psychiatry*. Jun 8 2019;92:217-225. doi:10.1016/j.pnpbp.2019.01.012
96. Georgopoulos AP, Tan HR, Lewis SM, et al. The synchronous neural interactions test as a functional neuromarker for post-traumatic stress disorder (PTSD): a robust classification method based on the bootstrap. *J Neural Eng*. Feb 2010;7(1):16011. doi:10.1088/1741-2560/7/1/016011
97. Godlewska BR, Browning M, Norbury R, Igoumenou A, Cowen PJ, Harmer CJ. Predicting Treatment Response in Depression: The Role of Anterior Cingulate Cortex. *Int J Neuropsychopharmacol*. Nov 1 2018;21(11):988-996. doi:10.1093/ijnp/pyy069
98. Godlewska BR, Browning M, Norbury R, Cowen PJ, Harmer CJ. Early changes in emotional processing as a marker of clinical response to SSRI treatment in depression. *Transl Psychiatry*. Nov 22 2016;6(11):e957. doi:10.1038/tp.2016.130
99. Goldstein-Piekarski AN, Staveland BR, Ball TM, Yesavage J, Korgaonkar MS, Williams LM. Intrinsic functional connectivity predicts remission on antidepressants: a randomized controlled trial to identify clinically applicable imaging biomarkers. *Transl Psychiatry*. Mar 6 2018;8(1):57. doi:10.1038/s41398-018-0100-3
100. Gong Q, Li L, Du M, et al. Quantitative prediction of individual psychopathology in trauma survivors using resting-state FMRI. *Neuropsychopharmacology*. Feb 2014;39(3):681-7. doi:10.1038/npp.2013.251
101. Gong Q, Li L, Tognin S, et al. Using structural neuroanatomy to identify trauma survivors with and without post-traumatic stress disorder at the individual level. *Psychol Med*. Jan 2014;44(1):195-203. doi:10.1017/S0033291713000561
102. Gong Q, Wu Q, Scarpazza C, et al. Prognostic prediction of therapeutic response in depression using high-field MR imaging. *Neuroimage*. Apr 15 2011;55(4):1497-503. doi:10.1016/j.neuroimage.2010.11.079
103. Gosnell SN, Curtis KN, Velasquez K, et al. Habenular connectivity may predict treatment response in depressed psychiatric inpatients. *J Affect Disord*. Jan 1 2019;242:211-219. doi:10.1016/j.jad.2018.08.026
104. Gowin JL, Ball TM, Wittmann M, Tapert SF, Paulus MP. Individualized relapse prediction: Personality measures and striatal and insular activity during reward-processing robustly predict relapse. *Drug Alcohol Depend*. Jul 1 2015;152:93-101. Erratum in: Drug Alcohol Depend. 2017 Jun 1;175:255 PMID: 28431752 [https://www.ncbi.nlm.nih.gov/pubmed/28431752]. doi:10.1016/j.drugalcdep.2015.04.018
105. Grieve SM, Korgaonkar MS, Gordon E, Williams LM, Rush AJ. Prediction of nonremission to antidepressant therapy using diffusion tensor imaging. *J Clin Psychiatry*. Apr 2016;77(4):e436-43. doi:10.4088/JCP.14m09577
106. Grotegerd D, Stuhrmann A, Kugel H, et al. Amygdala excitability to subliminally presented emotional faces distinguishes unipolar and bipolar depression: an fMRI and pattern classification study. *Hum Brain Mapp*. Jul 2014;35(7):2995-3007. doi:10.1002/hbm.22380

107. Guo M, Wang T, Zhang Z, et al. Diagnosis of major depressive disorder using whole-brain effective connectivity networks derived from resting-state functional MRI. *J Neural Eng*. Oct 23 2020;17(5):056038. doi:10.1088/1741-2552/abbc28
108. Guo W, Cui X, Liu F, et al. Decreased interhemispheric coordination in the posterior default-mode network and visual regions as trait alterations in first-episode, drug-naive major depressive disorder. *Brain Imaging Behav*. Oct 2018;12(5):1251-1258. doi:10.1007/s11682-017-9794-8
109. Guo H, Cao X, Liu Z, Li H, Chen J, Zhang K. Machine learning classifier using abnormal brain network topological metrics in major depressive disorder. *Neuroreport*. Dec 5 2012;23(17):1006-11. doi:10.1097/WNR.0b013e32835a650c
110. Guo WB, Liu F, Chen JD, et al. Abnormal neural activity of brain regions in treatment-resistant and treatment-sensitive major depressive disorder: a resting-state fMRI study. *J Psychiatr Res*. Oct 2012;46(10):1366-73. doi:10.1016/j.jpsychires.2012.07.003
111. Gyurak A, Patenaude B, Korgaonkar MS, Grieve SM, Williams LM, Etkin A. Frontoparietal Activation During Response Inhibition Predicts Remission to Antidepressants in Patients With Major Depression. *Biol Psychiatry*. Feb 15 2016;79(4):274-81. Comment in: Biol Psychiatry. 2016 Feb 15;79(4):262-3 PMID: 26796875 [https://www.ncbi.nlm.nih.gov/pubmed/26796875]. doi:10.1016/j.biopsych.2015.02.037
112. Hahn T, Kircher T, Straube B, et al. Predicting treatment response to cognitive behavioral therapy in panic disorder with agoraphobia by integrating local neural information. *JAMA Psychiatry*. Jan 2015;72(1):68-74. doi:10.1001/jamapsychiatry.2014.1741
113. Hahn T, Marquand AF, Ehlis AC, et al. Integrating neurobiological markers of depression. *Arch Gen Psychiatry*. Apr 2011;68(4):361-8. doi:10.1001/archgenpsychiatry.2010.178
114. Hasanzadeh F, Mohebbi M, Rostami R. Graph theory analysis of directed functional brain networks in major depressive disorder based on EEG signal. *J Neural Eng*. Mar 27 2020;17(2):026010. doi:10.1088/1741-2552/ab7613
115. Hasanzadeh F, Mohebbi M, Rostami R. Prediction of rTMS treatment response in major depressive disorder using machine learning techniques and nonlinear features of EEG signal. *J Affect Disord*. Sep 1 2019;256:132-142. doi:10.1016/j.jad.2019.05.070
116. He Z, Sheng W, Lu F, et al. Altered resting-state cerebral blood flow and functional connectivity of striatum in bipolar disorder and major depressive disorder. *Prog Neuropsychopharmacol Biol Psychiatry*. Mar 2 2019;90:177-185. doi:10.1016/j.pnpbp.2018.11.009
117. Hellewell SC, Welton T, Maller JJ, et al. Profound and reproducible patterns of reduced regional gray matter characterize major depressive disorder. *Transl Psychiatry*. Jul 24 2019;9(1):176. doi:10.1038/s41398-019-0512-8
118. Hopman HJ, Chan SMS, Chu WCW, et al. Personalized prediction of transcranial magnetic stimulation clinical response in patients with treatment-refractory depression using neuroimaging biomarkers and machine learning. *J Affect Disord*. Jul 1 2021;290:261-271. doi:10.1016/j.jad.2021.04.081
119. Hou Z, Kong Y, Yin Y, Zhang Y, Yuan Y. Identification of first-episode unmedicated major depressive disorder using pretreatment features of dominant coactivation patterns. *Prog Neuropsychopharmacol Biol Psychiatry*. Jan 10 2021;104:110038. doi:10.1016/j.pnpbp.2020.110038

120. Hou Z, Kong Y, He X, Yin Y, Zhang Y, Yuan Y. Increased temporal variability of striatum region facilitating the early antidepressant response in patients with major depressive disorder. *Prog Neuropsychopharmacol Biol Psychiatry*. Jul 13 2018;85:39-45. doi:10.1016/j.pnpbp.2018.03.026
121. Hou Z, Gong L, Zhi M, et al. Distinctive pretreatment features of bilateral nucleus accumbens networks predict early response to antidepressants in major depressive disorder. *Brain Imaging Behav*. Aug 2018;12(4):1042-1052. doi:10.1007/s11682-017-9773-0
122. Hou Z, Song X, Jiang W, et al. Prognostic value of imbalanced interhemispheric functional coordination in early therapeutic efficacy in major depressive disorder. *Psychiatry Res Neuroimaging*. Sep 30 2016;255:1-8. doi:10.1016/j.pscychresns.2016.07.011
123. Hu X, Zhang L, Hu X, et al. Abnormal Hippocampal Subfields May Be Potential Predictors of Worse Early Response to Antidepressant Treatment in Drug-Naive Patients With Major Depressive Disorder. *J Magn Reson Imaging*. Jun 2019;49(6):1760-1768. doi:10.1002/jmri.26520
124. Hu X, Zhang L, Bu X, et al. Localized Connectivity in Obsessive-Compulsive Disorder: An Investigation Combining Univariate and Multivariate Pattern Analyses. *Front Behav Neurosci*. 2019;13:122. doi:10.3389/fnbeh.2019.00122
125. Hu X, Liu Q, Li B, et al. Multivariate pattern analysis of obsessive-compulsive disorder using structural neuroanatomy. *Eur Neuropsychopharmacol*. Feb 2016;26(2):246-254. doi:10.1016/j.euroneuro.2015.12.014
126. Ichikawa N, Lisi G, Yahata N, et al. Primary functional brain connections associated with melancholic major depressive disorder and modulation by antidepressants. *Sci Rep*. Feb 26 2020;10(1):3542. Erratum in: Sci Rep. 2020 Oct 14;10(1):17650 PMID: 33057026 [https://www.ncbi.nlm.nih.gov/pubmed/33057026]. doi:10.1038/s41598-020-60527-z
127. Im JJ, Kim B, Hwang J, et al. Diagnostic potential of multimodal neuroimaging in posttraumatic stress disorder. *PLoS One*. 2017;12(5):e0177847. doi:10.1371/journal.pone.0177847
128. Isserles M, Daskalakis ZJ, George MS, Blumberger DM, Sackeim HA, Shahaf G. Simple Electroencephalographic Treatment-Emergent Marker Can Predict Repetitive Transcranial Magnetic Stimulation Antidepressant Response-A Feasibility Study. *J ECT*. Dec 2018;34(4):274-282. doi:10.1097/YCT.0000000000000551
129. James LM, Leuthold AF, Georgopoulos AP. Classification of posttraumatic stress disorder and related outcomes in women veterans using magnetoencephalography. *Exp Brain Res*. Apr 2022;240(4):1117-1125. doi:10.1007/s00221-022-06320-y
130. Januszko P, Gmaj B, Piotrowski T, et al. Delta resting-state functional connectivity in the cognitive control network as a prognostic factor for maintaining abstinence: An eLORETA preliminary study. *Drug Alcohol Depend*. Jan 1 2021;218:108393. doi:10.1016/j.drugalcdep.2020.108393
131. Jaworska N, de la Salle S, Ibrahim MH, Blier P, Knott V. Leveraging Machine Learning Approaches for Predicting Antidepressant Treatment Response Using Electroencephalography (EEG) and Clinical Data. *Front Psychiatry*. 2018;9:768. doi:10.3389/fpsyt.2018.00768
132. Jaworska N, Blondeau C, Tessier P, et al. Examining relations between alpha power as well as anterior cingulate cortex-localized theta activity and response to single or dual antidepressant pharmacotherapies. *J Psychopharmacol*. Jun 2014;28(6):587-95. doi:10.1177/0269881114523862

133. Jaworska N, Blondeau C, Tessier P, et al. Response prediction to antidepressants using scalp and source-localized loudness dependence of auditory evoked potential (LDAEP) slopes. *Prog Neuropsychopharmacol Biol Psychiatry*. Jul 1 2013;44:100-7. doi:10.1016/j.pnpbp.2013.01.012
134. Jiang C, Li Y, Tang Y, Guan C. Enhancing EEG-Based Classification of Depression Patients Using Spatial Information. *IEEE Trans Neural Syst Rehabil Eng*. 2021;29:566-575. doi:10.1109/TNSRE.2021.3059429
135. Jiang H, Dai Z, Lu Q, Yao Z. Magnetoencephalography resting-state spectral fingerprints distinguish bipolar depression and unipolar depression. *Bipolar Disord*. Sep 2020;22(6):612-620. doi:10.1111/bdi.12871
136. Jiang R, Abbott CC, Jiang T, et al. SMRI Biomarkers Predict Electroconvulsive Treatment Outcomes: Accuracy with Independent Data Sets. *Neuropsychopharmacology*. Apr 2018;43(5):1078-1087. doi:10.1038/npp.2017.165
137. Kamarajan C, Ardekani BA, Pandey AK, et al. Random Forest Classification of Alcohol Use Disorder Using EEG Source Functional Connectivity, Neuropsychological Functioning, and Impulsivity Measures. *Behav Sci (Basel)*. Mar 1 2020;10(3)doi:10.3390/bs10030062
138. Karim HT, Wang M, Andreescu C, et al. Acute trajectories of neural activation predict remission to pharmacotherapy in late-life depression. *Neuroimage Clin*. 2018;19:831-839. doi:10.1016/j.nicl.2018.06.006
139. Kaufman J, Sullivan GM, Yang J, et al. Quantification of the Serotonin 1A Receptor Using PET: Identification of a Potential Biomarker of Major Depression in Males. *Neuropsychopharmacology*. Jun 2015;40(7):1692-9. doi:10.1038/npp.2015.15
140. Kim YW, Kim S, Shim M, et al. Riemannian classifier enhances the accuracy of machine-learning-based diagnosis of PTSD using resting EEG. *Prog Neuropsychopharmacol Biol Psychiatry*. Aug 30 2020;102:109960. doi:10.1016/j.pnpbp.2020.109960
141. Kinreich S, McCutcheon VV, Aliev F, et al. Predicting alcohol use disorder remission: a longitudinal multimodal multi-featured machine learning approach. *Transl Psychiatry*. Mar 15 2021;11(1):166. doi:10.1038/s41398-021-01281-2
142. Kipli K, Kouzani AZ. Degree of contribution (DoC) feature selection algorithm for structural brain MRI volumetric features in depression detection. *Int J Comput Assist Radiol Surg*. Jul 2015;10(7):1003-16. doi:10.1007/s11548-014-1130-9
143. Klumpp H, Jimmy J, Burkhouse KL, et al. Brain response to emotional faces in anxiety and depression: neural predictors of cognitive behavioral therapy outcome and predictor-based subgroups following therapy. *Psychol Med*. Nov 10 2020:1-11. doi:10.1017/S0033291720003979
144. Klumpp H, Roberts J, Kennedy AE, et al. Emotion regulation related neural predictors of cognitive behavioral therapy response in social anxiety disorder. *Prog Neuropsychopharmacol Biol Psychiatry*. Apr 3 2017;75:106-112. doi:10.1016/j.pnpbp.2017.01.010
145. Koller-Schlaud K, Strohle A, Barwolf E, Behr J, Rentzsch J. EEG Frontal Asymmetry and Theta Power in Unipolar and Bipolar Depression. *J Affect Disord*. Nov 1 2020;276:501-510. doi:10.1016/j.jad.2020.07.011
146. Koo PC, Berger C, Kronenberg G, et al. Combined cognitive, psychomotor and electrophysiological biomarkers in major depressive disorder. *Eur Arch Psychiatry Clin Neurosci*. Oct 2019;269(7):823-832. doi:10.1007/s00406-018-0952-9

147. Korgaonkar MS, Goldstein-Piekarski AN, Fornito A, Williams LM. Intrinsic connectomes are a predictive biomarker of remission in major depressive disorder. *Mol Psychiatry*. Jul 2020;25(7):1537-1549. doi:10.1038/s41380-019-0574-2
148. Korgaonkar MS, Rekshan W, Gordon E, et al. Magnetic Resonance Imaging Measures of Brain Structure to Predict Antidepressant Treatment Outcome in Major Depressive Disorder. *EBioMedicine*. Jan 2015;2(1):37-45. doi:10.1016/j.ebiom.2014.12.002
149. Korgaonkar MS, Williams LM, Song YJ, Usherwood T, Grieve SM. Diffusion tensor imaging predictors of treatment outcomes in major depressive disorder. *Br J Psychiatry*. Oct 2014;205(4):321-8. doi:10.1192/bjp.bp.113.140376
150. Korgaonkar MS, Cooper NJ, Williams LM, Grieve SM. Mapping inter-regional connectivity of the entire cortex to characterize major depressive disorder: a whole-brain diffusion tensor imaging tractography study. *Neuroreport*. Jun 20 2012;23(9):566-71. doi:10.1097/WNR.0b013e3283546264
151. Kraus C, Klobl M, Tik M, et al. The pulvinar nucleus and antidepressant treatment: dynamic modeling of antidepressant response and remission with ultra-high field functional MRI. *Mol Psychiatry*. May 2019;24(5):746-756. Erratum in: Mol Psychiatry. 2018 Mar 8;: PMID: 29520037 [https://www.ncbi.nlm.nih.gov/pubmed/29520037]. doi:10.1038/s41380-017-0009-x
152. Kwak S, Kim M, Kim T, et al. Defining data-driven subgroups of obsessive-compulsive disorder with different treatment responses based on resting-state functional connectivity. *Transl Psychiatry*. Oct 26 2020;10(1):359. doi:10.1038/s41398-020-01045-4
153. Lanka P, Rangaprakash D, Dretsch MN, Katz JS, Denney TS, Jr., Deshpande G. Supervised machine learning for diagnostic classification from large-scale neuroimaging datasets. *Brain Imaging Behav*. Dec 2020;14(6):2378-2416. doi:10.1007/s11682-019-00191-8
154. Laxminarayan S, Wang C, Oyama T, Cashmere JD, Germain A, Reifman J. Identification of Veterans With PTSD Based on EEG Features Collected During Sleep. *Front Psychiatry*. 2020;11:532623. doi:10.3389/fpsyt.2020.532623
155. Leaver AM, Wade B, Vasavada M, et al. Fronto-Temporal Connectivity Predicts ECT Outcome in Major Depression. *Front Psychiatry*. 2018;9:92. doi:10.3389/fpsyt.2018.00092
156. Lebedeva AK, Westman E, Borza T, et al. MRI-Based Classification Models in Prediction of Mild Cognitive Impairment and Dementia in Late-Life Depression. *Front Aging Neurosci*. 2017;9:13. doi:10.3389/fnagi.2017.00013
157. Lee TW, Wu YT, Yu YW, Chen MC, Chen TJ. The implication of functional connectivity strength in predicting treatment response of major depressive disorder: a resting EEG study. *Psychiatry Res*. Dec 30 2011;194(3):372-377. doi:10.1016/j.pscychresns.2011.02.009
158. Li R, Yang J, Li L, et al. Integrating Multilevel Functional Characteristics Reveals Aberrant Neural Patterns during Audiovisual Emotional Processing in Depression. *Cereb Cortex*. Nov 23 2021;32(1):1-14. doi:10.1093/cercor/bhab185
159. Li CT, Cheng CM, Juan CH, et al. Task-Modulated Brain Activity Predicts Antidepressant Responses of Prefrontal Repetitive Transcranial Magnetic Stimulation: A Randomized Sham-Control Study. *Chronic Stress (Thousand Oaks)*. Jan-Dec 2021;5:24705470211006855. doi:10.1177/24705470211006855
160. Li H, Song S, Wang D, et al. Individualized diagnosis of major depressive disorder via multivariate pattern analysis of thalamic sMRI features. *BMC Psychiatry*. Aug 20 2021;21(1):415. doi:10.1186/s12888-021-03414-9

161. Li J, Chen H, Fan F, et al. White-matter functional topology: a neuromarker for classification and prediction in unmedicated depression. *Transl Psychiatry*. Oct 30 2020;10(1):365. doi:10.1038/s41398-020-01053-4
162. Li H, Cui L, Cao L, et al. Identification of bipolar disorder using a combination of multimodality magnetic resonance imaging and machine learning techniques. *BMC Psychiatry*. Oct 6 2020;20(1):488. doi:10.1186/s12888-020-02886-5
163. Li CT, Cheng CM, Chen MH, et al. Antidepressant Efficacy of Prolonged Intermittent Theta Burst Stimulation Monotherapy for Recurrent Depression and Comparison of Methods for Coil Positioning: A Randomized, Double-Blind, Sham-Controlled Study. *Biol Psychiatry*. Mar 1 2020;87(5):443-450. Comment in: Biol Psychiatry. 2020 Mar 1;87(5):384-385 PMID: 32029073 [https://www.ncbi.nlm.nih.gov/pubmed/32029073]. doi:10.1016/j.biopsych.2019.07.031
164. Li X, La R, Wang Y, et al. EEG-based mild depression recognition using convolutional neural network. *Med Biol Eng Comput*. Jun 2019;57(6):1341-1352. doi:10.1007/s11517-019-01959-2
165. Li Y, Cui Z, Liao Q, et al. Support vector machine-based multivariate pattern classification of methamphetamine dependence using arterial spin labeling. *Addict Biol*. Nov 2019;24(6):1254-1262. doi:10.1111/adb.12705
166. Li M, Das T, Deng W, et al. Clinical utility of a short resting-state MRI scan in differentiating bipolar from unipolar depression. *Acta Psychiatr Scand*. Sep 2017;136(3):288-299. doi:10.1111/acps.12752
167. Li CT, Hsieh JC, Huang HH, et al. Cognition-Modulated Frontal Activity in Prediction and Augmentation of Antidepressant Efficacy: A Randomized Controlled Pilot Study. *Cereb Cortex*. Jan 2016;26(1):202-10. doi:10.1093/cercor/bhu191
168. Li F, Huang X, Tang W, et al. Multivariate pattern analysis of DTI reveals differential white matter in individuals with obsessive-compulsive disorder. *Hum Brain Mapp*. Jun 2014;35(6):2643-51. doi:10.1002/hbm.22357
169. Li Y, Dai X, Wu H, Wang L. Establishment of Effective Biomarkers for Depression Diagnosis With Fusion of Multiple Resting-State Connectivity Measures. *Front Neurosci*. 2021;15:729958. doi:10.3389/fnins.2021.729958
170. Liao W, Li J, Duan X, Cui Q, Chen H, Chen H. Static and dynamic connectomics differentiate between depressed patients with and without suicidal ideation. *Hum Brain Mapp*. Oct 2018;39(10):4105-4118. doi:10.1002/hbm.24235
171. Liu S, Liu X, Yan D, et al. Alterations in Patients With First-Episode Depression in the Eyes-Open and Eyes-Closed Conditions: A Resting-State EEG Study. *IEEE Trans Neural Syst Rehabil Eng*. 2022;30:1019-1029. doi:10.1109/TNSRE.2022.3166824
172. Liu J, Bu X, Hu X, et al. Temporal variability of regional intrinsic neural activity in drug-naive patients with obsessive-compulsive disorder. *Hum Brain Mapp*. Aug 15 2021;42(12):3792-3803. doi:10.1002/hbm.25465
173. Liu W, Hua M, Qin J, et al. Disrupted pathways from frontal-parietal cortex to basal ganglia and cerebellum in patients with unmedicated obsessive compulsive disorder as observed by whole-brain resting-state effective connectivity analysis - a small sample pilot study. *Brain Imaging Behav*. Jun 2021;15(3):1344-1354. doi:10.1007/s11682-020-00333-3
174. Liu W, Qin J, Tang Q, et al. Disrupted pathways from the frontal-parietal cortices to basal nuclei and the cerebellum are a feature of the obsessive-compulsive disorder spectrum and can be used to aid in early differential diagnosis. *Psychiatry Res*. Nov 2020;293:113436. doi:10.1016/j.psychres.2020.113436

175. Liu W, Zhang C, Wang X, et al. Functional connectivity of major depression disorder using ongoing EEG during music perception. *Clin Neurophysiol*. Oct 2020;131(10):2413-2422. doi:10.1016/j.clinph.2020.06.031
176. Liu Y, Admon R, Mellem MS, et al. Machine Learning Identifies Large-Scale Reward-Related Activity Modulated by Dopaminergic Enhancement in Major Depression. *Biol Psychiatry Cogn Neurosci Neuroimaging*. Feb 2020;5(2):163-172. Comment in: Biol Psychiatry Cogn Neurosci Neuroimaging. 2020 Feb;5(2):133-134 PMID: 32035610 [https://www.ncbi.nlm.nih.gov/pubmed/32035610]. doi:10.1016/j.bpsc.2019.10.002
177. Liu F, Xie B, Wang Y, et al. Characterization of post-traumatic stress disorder using resting-state fMRI with a multi-level parametric classification approach. *Brain Topogr*. Mar 2015;28(2):221-37. doi:10.1007/s10548-014-0386-2
178. Liu F, Guo W, Fouche JP, et al. Multivariate classification of social anxiety disorder using whole brain functional connectivity. *Brain Struct Funct*. Jan 2015;220(1):101-15. doi:10.1007/s00429-013-0641-4
179. Liu TY, Chen YS, Su TP, Hsieh JC, Chen LF. Abnormal early gamma responses to emotional faces differentiate unipolar from bipolar disorder patients. *Biomed Res Int*. 2014;2014:906104. doi:10.1155/2014/906104
180. Liu F, Guo W, Yu D, et al. Classification of different therapeutic responses of major depressive disorder with multivariate pattern analysis method based on structural MR scans. *PLoS One*. 2012;7(7):e40968. doi:10.1371/journal.pone.0040968
181. Lord A, Horn D, Breakspear M, Walter M. Changes in community structure of resting state functional connectivity in unipolar depression. *PLoS One*. 2012;7(8):e41282. doi:10.1371/journal.pone.0041282
182. Lu F, Cui Q, He Z, et al. Prefrontal-limbic-striatum dysconnectivity associated with negative emotional endophenotypes in bipolar disorder during depressive episodes. *J Affect Disord*. Dec 1 2021;295:422-430. doi:10.1016/j.jad.2021.08.055
183. Lu Q, Jiang H, Luo G, Han Y, Yao Z. Multichannel matching pursuit of MEG signals for discriminative oscillation pattern detection in depression. *Int J Psychophysiol*. May 2013;88(2):206-12. doi:10.1016/j.ijpsycho.2013.04.010
184. Luo Q, Liu W, Jin L, Chang C, Peng Z. Classification of Obsessive-Compulsive Disorder Using Distance Correlation on Resting-State Functional MRI Images. *Front Neuroinform*. 2021;15:676491. doi:10.3389/fninf.2021.676491
185. Lv D, Ou Y, Wang Y, et al. Altered Functional Connectivity Strength at Rest in Medication-Free Obsessive-Compulsive Disorder. *Neural Plast*. 2021;2021:3741104. doi:10.1155/2021/3741104
186. Manelis A, Iyengar S, Swartz HA, Phillips ML. Prefrontal cortical activation during working memory task anticipation contributes to discrimination between bipolar and unipolar depression. *Neuropsychopharmacology*. May 2020;45(6):956-963. doi:10.1038/s41386-020-0638-7
187. Matsuo K, Harada K, Fujita Y, et al. Distinctive Neuroanatomical Substrates for Depression in Bipolar Disorder versus Major Depressive Disorder. *Cereb Cortex*. Jan 1 2019;29(1):202-214. doi:10.1093/cercor/bhx319
188. Matsuoka K, Yasuno F, Kishimoto T, et al. Microstructural Differences in the Corpus Callosum in Patients With Bipolar Disorder and Major Depressive Disorder. *J Clin Psychiatry*. Jan 2017;78(1):99-104. doi:10.4088/JCP.15m09851

189. McHugh MJ, Demers CH, Salmeron BJ, Devous MD, Sr., Stein EA, Adinoff B. Cortico-amygdala coupling as a marker of early relapse risk in cocaine-addicted individuals. *Front Psychiatry*. 2014;5:16. doi:10.3389/fpsyt.2014.00016
190. Meng Q, Zhang A, Cao X, et al. Brain Imaging Study on the Pathogenesis of Depression & Therapeutic Effect of Selective Serotonin Reuptake Inhibitors. *Psychiatry Investig*. Jul 2020;17(7):688-694. doi:10.30773/pi.2020.0041
191. Meyer BM, Rabl U, Huemer J, et al. Prefrontal networks dynamically related to recovery from major depressive disorder: a longitudinal pharmacological fMRI study. *Transl Psychiatry*. Feb 4 2019;9(1):64. doi:10.1038/s41398-019-0395-8
192. Mishra P, Nizamie SH, Jahan M, et al. Predictors of chronicity in alcohol use disorder: an evoked response potential study. *J Addict Dis*. Oct-Dec 2020;38(4):411-419. doi:10.1080/10550887.2020.1780185
193. Modinos G, Mechelli A, Pettersson-Yeo W, Allen P, McGuire P, Aleman A. Pattern classification of brain activation during emotional processing in subclinical depression: psychosis proneness as potential confounding factor. *PeerJ*. 2013;1:e42. doi:10.7717/peerj.42
194. Mohammadi M, Al-Azab F, Raahemi B, et al. Data mining EEG signals in depression for their diagnostic value. *BMC Med Inform Decis Mak*. Dec 23 2015;15:108. doi:10.1186/s12911-015-0227-6
195. Mourao-Miranda J, Almeida JR, Hassel S, et al. Pattern recognition analyses of brain activation elicited by happy and neutral faces in unipolar and bipolar depression. *Bipolar Disord*. Jun 2012;14(4):451-60. doi:10.1111/j.1399-5618.2012.01019.x
196. Mulders PCR, Llera A, Beckmann CF, et al. Structural changes induced by electroconvulsive therapy are associated with clinical outcome. *Brain Stimul*. May - Jun 2020;13(3):696-704. Comment in: Brain Stimul. 2020 Sep - Oct;13(5):1226-1231 PMID: 32442625 [https://www.ncbi.nlm.nih.gov/pubmed/32442625]. doi:10.1016/j.brs.2020.02.020
197. Mumtaz W, Qayyum A. A deep learning framework for automatic diagnosis of unipolar depression. *Int J Med Inform*. Dec 2019;132:103983. doi:10.1016/j.ijmedinf.2019.103983
198. Mumtaz W, Malik AS. A Comparative Study of Different EEG Reference Choices for Diagnosing Unipolar Depression. *Brain Topogr*. Sep 2018;31(5):875-885. doi:10.1007/s10548-018-0651-x
199. Mumtaz W, Saad M, Kamel N, Ali SSA, Malik AS. An EEG-based functional connectivity measure for automatic detection of alcohol use disorder. *Artif Intell Med*. Jan 2018;84:79-89. doi:10.1016/j.artmed.2017.11.002
200. Mumtaz W, Ali SSA, Yasin MAM, Malik AS. A machine learning framework involving EEG-based functional connectivity to diagnose major depressive disorder (MDD). *Med Biol Eng Comput*. Feb 2018;56(2):233-246. doi:10.1007/s11517-017-1685-z
201. Mumtaz W, Xia L, Mohd Yasin MA, Azhar Ali SS, Malik AS. A wavelet-based technique to predict treatment outcome for Major Depressive Disorder. *PLoS One*. 2017;12(2):e0171409. doi:10.1371/journal.pone.0171409
202. Mumtaz W, Vuong PL, Xia L, Malik AS, Rashid RBA. An EEG-based machine learning method to screen alcohol use disorder. *Cogn Neurodyn*. Apr 2017;11(2):161-171. doi:10.1007/s11571-016-9416-y
203. Mumtaz W, Xia L, Ali SSA, Yasin MAM, Hussain M, Malik AS. Electroencephalogram (EEG)-based computer-aided technique to diagnose major depressive disorder (MDD). *Biomedical Signal Processing and Control*. 2017;31:108-115. doi:10.1016/j.bspc.2016.07.006

204. Mwangi B, Wu MJ, Cao B, et al. Individualized Prediction and Clinical Staging of Bipolar Disorders using Neuroanatomical Biomarkers. *Biol Psychiatry Cogn Neurosci Neuroimaging*. Mar 1 2016;1(2):186-194. doi:10.1016/j.bpsc.2016.01.001
205. Neumeister A, Normandin MD, Pietrzak RH, et al. Elevated brain cannabinoid CB1 receptor availability in post-traumatic stress disorder: a positron emission tomography study. *Mol Psychiatry*. Sep 2013;18(9):1034-40. doi:10.1038/mp.2013.61
206. Nguyen KP, Fatt CC, Treacher A, Mellema C, Trivedi MH, Montillo A. Predicting Response to the Antidepressant Bupropion using Pretreatment fMRI. *Predict Intell Med*. Oct 2019;11843:53-62. doi:10.1007/978-3-030-32281-6_6
207. Nicholson AA, Densmore M, McKinnon MC, et al. Machine learning multivariate pattern analysis predicts classification of posttraumatic stress disorder and its dissociative subtype: a multimodal neuroimaging approach. *Psychol Med*. Sep 2019;49(12):2049-2059. doi:10.1017/S0033291718002866
208. Niida R, Yamagata B, Niida A, Uechi A, Matsuda H, Mimura M. Aberrant Anterior Thalamic Radiation Structure in Bipolar Disorder: A Diffusion Tensor Tractography Study. *Front Psychiatry*. 2018;9:522. doi:10.3389/fpsyt.2018.00522
209. Niida A, Niida R, Matsuda H, Inada T, Motomura M, Uechi A. Identification of atrophy of the subgenual anterior cingulate cortex, in particular the subcallosal area, as an effective auxiliary means of diagnosis for major depressive disorder. *Int J Gen Med*. 2012;5:667-74. doi:10.2147/IJGM.S34093
210. Nogovitsyn N, Muller M, Souza R, et al. Hippocampal tail volume as a predictive biomarker of antidepressant treatment outcomes in patients with major depressive disorder: a CAN-BIND report. *Neuropsychopharmacology*. Jan 2020;45(2):283-291. doi:10.1038/s41386-019-0542-1
211. Nord CL, Halahakoon DC, Limbachya T, et al. Neural predictors of treatment response to brain stimulation and psychological therapy in depression: a double-blind randomized controlled trial. *Neuropsychopharmacology*. Aug 2019;44(9):1613-1622. doi:10.1038/s41386-019-0401-0
212. Olbrich S, Sander C, Minkwitz J, et al. EEG vigilance regulation patterns and their discriminative power to separate patients with major depression from healthy controls. *Neuropsychobiology*. Jun 2012;65(4):188-94. doi:10.1159/000337000
213. Oliveira-Maia AJ, Press D, Pascual-Leone A. Modulation of motor cortex excitability predicts antidepressant response to prefrontal cortex repetitive transcranial magnetic stimulation. *Brain Stimul*. Jul - Aug 2017;10(4):787-794. doi:10.1016/j.brs.2017.03.013
214. Yang J, Palaniyappan L, Xi C, et al. Aberrant integrity of the cortico-limbic-striatal circuit in major depressive disorder with suicidal ideation. *J Psychiatr Res*. Apr 2022;148:277-285. doi:10.1016/j.jpsychires.2022.02.003
215. Pang Y, Zhang H, Cui Q, et al. Combined static and dynamic functional connectivity signatures differentiating bipolar depression from major depressive disorder. *Aust N Z J Psychiatry*. Aug 2020;54(8):832-842. doi:10.1177/0004867420924089
216. Pantazatos SP, Talati A, Schneier FR, Hirsch J. Reduced anterior temporal and hippocampal functional connectivity during face processing discriminates individuals with social anxiety disorder from healthy controls and panic disorder, and increases following treatment. *Neuropsychopharmacology*. Jan 2014;39(2):425-34. doi:10.1038/npp.2013.211
217. Patel MJ, Andreescu C, Price JC, Edelman KL, Reynolds CF, 3rd, Aizenstein HJ. Machine learning approaches for integrating clinical and imaging features in late-life depression

classification and response prediction. *Int J Geriatr Psychiatry*. Oct 2015;30(10):1056-67. doi:10.1002/gps.4262
218. Pillai RL, Zhang M, Yang J, et al. Molecular connectivity disruptions in males with major depressive disorder. *J Cereb Blood Flow Metab*. Aug 2019;39(8):1623-1634. doi:10.1177/0271678X18764053
219. Price RB, Cummings L, Gilchrist D, et al. Towards personalized, brain-based behavioral intervention for transdiagnostic anxiety: Transient neural responses to negative images predict outcomes following a targeted computer-based intervention. *J Consult Clin Psychol*. Dec 2018;86(12):1031-1045. doi:10.1037/ccp0000309
220. Qiao J, Li A, Cao C, Wang Z, Sun J, Xu G. Aberrant Functional Network Connectivity as a Biomarker of Generalized Anxiety Disorder. *Front Hum Neurosci*. 2017;11:626. doi:10.3389/fnhum.2017.00626
221. Qin K, Lei D, Pinaya WHL, et al. Using graph convolutional network to characterize individuals with major depressive disorder across multiple imaging sites. *EBioMedicine*. Apr 2022;78:103977. doi:10.1016/j.ebiom.2022.103977
222. Qin J, Shen H, Zeng LL, Jiang W, Liu L, Hu D. Predicting clinical responses in major depression using intrinsic functional connectivity. *Neuroreport*. Aug 19 2015;26(12):675-80. doi:10.1097/WNR.0000000000000407
223. Qin J, Wei M, Liu H, et al. Abnormal hubs of white matter networks in the frontal-parieto circuit contribute to depression discrimination via pattern classification. *Magn Reson Imaging*. Dec 2014;32(10):1314-20. doi:10.1016/j.mri.2014.08.037
224. Qiu L, Huang X, Zhang J, et al. Characterization of major depressive disorder using a multiparametric classification approach based on high resolution structural images. *Journal of psychiatry & neuroscience : JPN*. 2014;39(2):78-86.
225. Rabinoff M, Kitchen CM, Cook IA, Leuchter AF. Evaluation of quantitative EEG by classification and regression trees to characterize responders to antidepressant and placebo treatment. *Open Med Inform J*. 2011;5:1-8. doi:10.2174/1874431101105010001
226. Raji CA, Willeumier K, Taylor D, et al. Functional neuroimaging with default mode network regions distinguishes PTSD from TBI in a military veteran population. *Brain Imaging Behav*. Sep 2015;9(3):527-34. doi:10.1007/s11682-015-9385-5
227. Rangaprakash D, Dretsch MN, Venkataraman A, Katz JS, Denney TS, Jr., Deshpande G. Identifying disease foci from static and dynamic effective connectivity networks: Illustration in soldiers with trauma. *Hum Brain Mapp*. Jan 2018;39(1):264-287. doi:10.1002/hbm.23841
228. Rangaprakash D, Deshpande G, Daniel TA, et al. Compromised hippocampus-striatum pathway as a potential imaging biomarker of mild-traumatic brain injury and posttraumatic stress disorder. *Hum Brain Mapp*. Jun 2017;38(6):2843-2864. doi:10.1002/hbm.23551
229. Rangaprakash D, Dretsch MN, Katz JS, Denney TS, Jr., Deshpande G. Dynamics of Segregation and Integration in Directional Brain Networks: Illustration in Soldiers With PTSD and Neurotrauma. *Front Neurosci*. 2019;13:803. doi:10.3389/fnins.2019.00803
230. Redlich R, Almeida JJ, Grotegerd D, et al. Brain morphometric biomarkers distinguishing unipolar and bipolar depression. A voxel-based morphometry-pattern classification approach. *JAMA Psychiatry*. Nov 2014;71(11):1222-30. Erratum in: JAMA Psychiatry. 2014 Dec 1;71(12):1408. doi:10.1001/jamapsychiatry.2014.1100
231. Reggente N, Moody TD, Morfini F, et al. Multivariate resting-state functional connectivity predicts response to cognitive behavioral therapy in obsessive-compulsive disorder. *Proc Natl Acad Sci U S A*. Feb 27 2018;115(9):2222-2227. doi:10.1073/pnas.1716686115

232. Rentzsch J, Adli M, Wiethoff K, Gomez-Carrillo de Castro A, Gallinat J. Pretreatment anterior cingulate activity predicts antidepressant treatment response in major depressive episodes. *Eur Arch Psychiatry Clin Neurosci*. Apr 2014;264(3):213-23. doi:10.1007/s00406-013-0424-1
233. Richieri R, Verger A, Boyer L, et al. Predictive value of dorso-lateral prefrontal connectivity for rTMS response in treatment-resistant depression: A brain perfusion SPECT study. *Brain Stimul*. Sep - Oct 2018;11(5):1093-1097. doi:10.1016/j.brs.2018.05.010
234. Richieri R, Boyer L, Farisse J, et al. Predictive value of brain perfusion SPECT for rTMS response in pharmacoresistant depression. *Eur J Nucl Med Mol Imaging*. Sep 2011;38(9):1715-22. doi:10.1007/s00259-011-1850-9
235. Rive MM, Redlich R, Schmaal L, et al. Distinguishing medication-free subjects with unipolar disorder from subjects with bipolar disorder: state matters. *Bipolar Disord*. Nov 2016;18(7):612-623. doi:10.1111/bdi.12446
236. Rocha-Rego V, Jogia J, Marquand AF, Mourao-Miranda J, Simmons A, Frangou S. Examination of the predictive value of structural magnetic resonance scans in bipolar disorder: a pattern classification approach. *Psychol Med*. Feb 2014;44(3):519-32. doi:10.1017/S0033291713001013
237. Rottstaedt F, Weidner K, Strauss T, et al. Size matters - The olfactory bulb as a marker for depression. *J Affect Disord*. Mar 15 2018;229:193-198. doi:10.1016/j.jad.2017.12.047
238. Rubin-Falcone H, Zanderigo F, Thapa-Chhetry B, et al. Pattern recognition of magnetic resonance imaging-based gray matter volume measurements classifies bipolar disorder and major depressive disorder. *J Affect Disord*. Feb 2018;227:498-505. doi:10.1016/j.jad.2017.11.043
239. Sacchet MD, Livermore EE, Iglesias JE, Glover GH, Gotlib IH. Subcortical volumes differentiate Major Depressive Disorder, Bipolar Disorder, and remitted Major Depressive Disorder. *J Psychiatr Res*. Sep 2015;68:91-8. doi:10.1016/j.jpsychires.2015.06.002
240. Sadat Shahabi M, Shalbaf A, Maghsoudi A. Prediction of drug response in major depressive disorder using ensemble of transfer learning with convolutional neural network based on EEG. *Biocybernetics and Biomedical Engineering*. 2021;41(3):946-959. doi:10.1016/j.bbe.2021.06.006
241. Sankar A, Zhang T, Gaonkar B, et al. Diagnostic potential of structural neuroimaging for depression from a multi-ethnic community sample. *BJPsych Open*. Jul 2016;2(4):247-254. doi:10.1192/bjpo.bp.115.002493
242. Schmaal L, Marquand AF, Rhebergen D, et al. Predicting the Naturalistic Course of Major Depressive Disorder Using Clinical and Multimodal Neuroimaging Information: A Multivariate Pattern Recognition Study. *Biol Psychiatry*. Aug 15 2015;78(4):278-86. doi:10.1016/j.biopsych.2014.11.018
243. Schnack HG, Nieuwenhuis M, van Haren NE, et al. Can structural MRI aid in clinical classification? A machine learning study in two independent samples of patients with schizophrenia, bipolar disorder and healthy subjects. *Neuroimage*. Jan 1 2014;84:299-306. doi:10.1016/j.neuroimage.2013.08.053
244. Schnyer DM, Clasen PC, Gonzalez C, Beevers CG. Evaluating the diagnostic utility of applying a machine learning algorithm to diffusion tensor MRI measures in individuals with major depressive disorder. *Psychiatry Res Neuroimaging*. Jun 30 2017;264:1-9. doi:10.1016/j.pscychresns.2017.03.003

245. Schultz J, Becker B, Preckel K, et al. Improving therapy outcome prediction in major depression using multimodal functional neuroimaging: A pilot study. *Personalized Medicine in Psychiatry*. 2018;11-12:7-15. doi:10.1016/j.pmip.2018.09.001
246. Sekutowicz M, Guggenmos M, Kuitunen-Paul S, et al. Neural Response Patterns During Pavlovian-to-Instrumental Transfer Predict Alcohol Relapse and Young Adult Drinking. *Biol Psychiatry*. Dec 1 2019;86(11):857-863. Comment in: Biol Psychiatry. 2019 Dec 1;86(11):807-808 PMID: 31668220 [https://www.ncbi.nlm.nih.gov/pubmed/31668220]. doi:10.1016/j.biopsych.2019.06.028
247. Serpa MH, Ou Y, Schaufelberger MS, et al. Neuroanatomical classification in a population-based sample of psychotic major depression and bipolar I disorder with 1 year of diagnostic stability. *Biomed Res Int*. 2014;2014:706157. doi:10.1155/2014/706157
248. Shalbaf R, Brenner C, Pang C, et al. Non-linear Entropy Analysis in EEG to Predict Treatment Response to Repetitive Transcranial Magnetic Stimulation in Depression. *Front Pharmacol*. 2018;9:1188. doi:10.3389/fphar.2018.01188
249. Shan X, Qiu Y, Pan P, et al. Disrupted Regional Homogeneity in Drug-Naive Patients With Bipolar Disorder. *Front Psychiatry*. 2020;11:825. doi:10.3389/fpsyt.2020.00825
250. Shan X, Cui X, Liu F, et al. Shared and distinct homotopic connectivity changes in melancholic and non-melancholic depression. *J Affect Disord*. May 15 2021;287:268-275. doi:10.1016/j.jad.2021.03.038
251. Shao J, Dai Z, Zhu R, et al. Early identification of bipolar from unipolar depression before manic episode: Evidence from dynamic rfMRI. *Bipolar Disord*. Dec 2019;21(8):774-784. doi:10.1111/bdi.12819
252. Shi Y, Zhang L, He C, et al. Sleep disturbance-related neuroimaging features as potential biomarkers for the diagnosis of major depressive disorder: A multicenter study based on machine learning. *J Affect Disord*. Dec 1 2021;295:148-155. doi:10.1016/j.jad.2021.08.027
253. Shi J, Geng J, Yan R, et al. Differentiation of Transformed Bipolar Disorder From Unipolar Depression by Resting-State Functional Connectivity Within Reward Circuit. *Front Psychol*. 2018;9:2586. doi:10.3389/fpsyg.2018.02586
254. Shim M, Jin MJ, Im CH, Lee SH. Machine-learning-based classification between post-traumatic stress disorder and major depressive disorder using P300 features. *Neuroimage Clin*. 2019;24:102001. doi:10.1016/j.nicl.2019.102001
255. Shimizu Y, Yoshimoto J, Toki S, et al. Toward Probabilistic Diagnosis and Understanding of Depression Based on Functional MRI Data Analysis with Logistic Group LASSO. *PLoS One*. 2015;10(5):e0123524. doi:10.1371/journal.pone.0123524
256. Shu IW, Onton JA, O'Connell RM, Simmons AN, Matthews SC. Combat veterans with comorbid PTSD and mild TBI exhibit a greater inhibitory processing ERP from the dorsal anterior cingulate cortex. *Psychiatry Res*. Oct 30 2014;224(1):58-66. doi:10.1016/j.pscychresns.2014.07.010
257. Siegle GJ, Thompson WK, Collier A, et al. Toward clinically useful neuroimaging in depression treatment: prognostic utility of subgenual cingulate activity for determining depression outcome in cognitive therapy across studies, scanners, and patient characteristics. *Arch Gen Psychiatry*. Sep 2012;69(9):913-24. doi:10.1001/archgenpsychiatry.2012.65
258. Squarcina L, Dagnew TM, Rivolta MW, Bellani M, Sassi R, Brambilla P. Automated cortical thickness and skewness feature selection in bipolar disorder using a semi-supervised learning method. *J Affect Disord*. Sep 1 2019;256:416-423. doi:10.1016/j.jad.2019.06.019

259. Stange JP, Jenkins LM, Pocius S, et al. Using resting-state intrinsic network connectivity to identify suicide risk in mood disorders. *Psychol Med*. Oct 2020;50(14):2324-2334. doi:10.1017/S0033291719002356
260. Stout DM, Harle KM, Norman SB, Simmons AN, Spadoni AD. Resting-state connectivity subtype of comorbid PTSD and alcohol use disorder moderates improvement from integrated prolonged exposure therapy in Veterans. *Psychol Med*. Apr 30 2021:1-10. doi:10.1017/S0033291721001513
261. Stoyanov D, Kandilarova S, Paunova R, Barranco Garcia J, Latypova A, Kherif F. Cross-Validation of Functional MRI and Paranoid-Depressive Scale: Results From Multivariate Analysis. *Front Psychiatry*. 2019;10:869. doi:10.3389/fpsyt.2019.00869
262. Sun F, Liu Z, Yang J, Fan Z, Yang J. Differential Dynamical Pattern of Regional Homogeneity in Bipolar and Unipolar Depression: A Preliminary Resting-State fMRI Study. *Front Psychiatry*. 2021;12:764932. doi:10.3389/fpsyt.2021.764932
263. Sun K, Liu Z, Chen G, et al. A two-center radiomic analysis for differentiating major depressive disorder using multi-modality MRI data under different parcellation methods. *J Affect Disord*. Mar 1 2022;300:1-9. doi:10.1016/j.jad.2021.12.065
264. Sun F, Liu Z, Fan Z, Zuo J, Xi C, Yang J. Dynamical regional activity in putamen distinguishes bipolar type I depression and unipolar depression. *J Affect Disord*. Jan 15 2022;297:94-101. doi:10.1016/j.jad.2021.10.021
265. Sun H, Jiang R, Qi S, et al. Preliminary prediction of individual response to electroconvulsive therapy using whole-brain functional magnetic resonance imaging data. *Neuroimage Clin*. 2020;26:102080. doi:10.1016/j.nicl.2019.102080
266. Suo X, Lei D, Li W, et al. Individualized Prediction of PTSD Symptom Severity in Trauma Survivors From Whole-Brain Resting-State Functional Connectivity. *Front Behav Neurosci*. 2020;14:563152. doi:10.3389/fnbeh.2020.563152
267. Sverdlov O, Curcic J, Hannesdottir K, et al. A Study of Novel Exploratory Tools, Digital Technologies, and Central Nervous System Biomarkers to Characterize Unipolar Depression. *Front Psychiatry*. 2021;12:640741. doi:10.3389/fpsyt.2021.640741
268. Tahmasian M, Jamalabadi H, Abedini M, et al. Differentiation chronic post traumatic stress disorder patients from healthy subjects using objective and subjective sleep-related parameters. *Neurosci Lett*. May 22 2017;650:174-179. doi:10.1016/j.neulet.2017.04.042
269. Takagi Y, Sakai Y, Lisi G, et al. A Neural Marker of Obsessive-Compulsive Disorder from Whole-Brain Functional Connectivity. *Sci Rep*. Aug 8 2017;7(1):7538. doi:10.1038/s41598-017-07792-7
270. Tang Q, Cui Q, Chen Y, et al. Shared and distinct changes in local dynamic functional connectivity patterns in major depressive and bipolar depressive disorders. *J Affect Disord*. Feb 1 2022;298(Pt A):43-50. doi:10.1016/j.jad.2021.10.109
271. Taylor WD, McQuoid DR, Payne ME, Zannas AS, MacFall JR, Steffens DC. Hippocampus atrophy and the longitudinal course of late-life depression. *Am J Geriatr Psychiatry*. Dec 2014;22(12):1504-12. doi:10.1016/j.jagp.2013.11.004
272. Tekin Erguzel T, Tas C, Cebi M. A wrapper-based approach for feature selection and classification of major depressive disorder-bipolar disorders. *Comput Biol Med*. Sep 2015;64:127-37. doi:10.1016/j.compbiomed.2015.06.021
273. Tenke CE, Kayser J, Manna CG, et al. Current source density measures of electroencephalographic alpha predict antidepressant treatment response. *Biol Psychiatry*. Aug 15 2011;70(4):388-94. doi:10.1016/j.biopsych.2011.02.016

274. Tian S, Sun Y, Shao J, et al. Predicting escitalopram monotherapy response in depression: The role of anterior cingulate cortex. *Hum Brain Mapp*. Apr 1 2020;41(5):1249-1260. doi:10.1002/hbm.24872
275. Tsolaki E, Narr KL, Espinoza R, et al. Subcallosal Cingulate Structural Connectivity Differs in Responders and Nonresponders to Electroconvulsive Therapy. *Biol Psychiatry Cogn Neurosci Neuroimaging*. Jan 2021;6(1):10-19. doi:10.1016/j.bpsc.2020.05.010
276. Uyulan C, de la Salle S, Erguzel TT, et al. Depression Diagnosis Modeling With Advanced Computational Methods: Frequency-Domain eMVAR and Deep Learning. *Clin EEG Neurosci*. Jan 2022;53(1):24-36. doi:10.1177/15500594211018545
277. van Rooij SJ, Kennis M, Vink M, Geuze E. Predicting Treatment Outcome in PTSD: A Longitudinal Functional MRI Study on Trauma-Unrelated Emotional Processing. *Neuropsychopharmacology*. Mar 2016;41(4):1156-65. doi:10.1038/npp.2015.257
278. van Waarde JA, Scholte HS, van Oudheusden LJ, Verwey B, Denys D, van Wingen GA. A functional MRI marker may predict the outcome of electroconvulsive therapy in severe and treatment-resistant depression. *Mol Psychiatry*. May 2015;20(5):609-14. doi:10.1038/mp.2014.78
279. Voineskos D, Blumberger DM, Zomorrodi R, et al. Altered Transcranial Magnetic Stimulation-Electroencephalographic Markers of Inhibition and Excitation in the Dorsolateral Prefrontal Cortex in Major Depressive Disorder. *Biol Psychiatry*. Mar 15 2019;85(6):477-486. doi:10.1016/j.biopsych.2018.09.032
280. Wade BSC, Sui J, Njau S, et al. Data-Driven Cluster Selection for Subcortical Shape and Cortical Thickness Predicts Recovery from Depressive Symptoms. *Proc IEEE Int Symp Biomed Imaging*. Apr 2017;2017:502-506. doi:10.1109/ISBI.2017.7950570
281. Wade BS, Joshi SH, Njau S, et al. Effect of Electroconvulsive Therapy on Striatal Morphometry in Major Depressive Disorder. *Neuropsychopharmacology*. Sep 2016;41(10):2481-91. doi:10.1038/npp.2016.48
282. Wade BSC, Sui J, Hellemann G, et al. Inter and intra-hemispheric structural imaging markers predict depression relapse after electroconvulsive therapy: a multisite study. *Transl Psychiatry*. Dec 8 2017;7(12):1270. doi:10.1038/s41398-017-0020-7
283. Wang Q, Tian S, Zhao P, Cao Q, Lu Q, Yao Z. Association Between Antidepressant Efficacy and Interactions of Three Core Depression-Related Brain Networks in Major Depressive Disorder. *Front Psychiatry*. 2022;13:862507. doi:10.3389/fpsyt.2022.862507
284. Wang Y, Gong N, Fu C. Major depression disorder diagnosis and analysis based on structural magnetic resonance imaging and deep learning. *J Integr Neurosci*. Dec 30 2021;20(4):977-984. doi:10.31083/j.jin2004098
285. Wang Y, Sun K, Liu Z, et al. Classification of Unmedicated Bipolar Disorder Using Whole-Brain Functional Activity and Connectivity: A Radiomics Analysis. *Cereb Cortex*. Mar 14 2020;30(3):1117-1128. doi:10.1093/cercor/bhz152
286. Wang YM, Cai XL, Zhang RT, et al. Searchlight classification based on Amplitude of Low Frequency Fluctuation and functional connectivity in individuals with obsessive-compulsive symptoms. *Cogn Neuropsychiatry*. Sep 2019;24(5):322-334. doi:10.1080/13546805.2019.1658575
287. Wang Q, Tian S, Tang H, et al. Identification of major depressive disorder and prediction of treatment response using functional connectivity between the prefrontal cortices and subgenual anterior cingulate: A real-world study. *J Affect Disord*. Jun 1 2019;252:365-372. doi:10.1016/j.jad.2019.04.046

288. Wang Y, Wang J, Jia Y, et al. Topologically convergent and divergent functional connectivity patterns in unmedicated unipolar depression and bipolar disorder. *Transl Psychiatry*. Jul 4 2017;7(7):e1165. doi:10.1038/tp.2017.117
289. Wang X, Ren Y, Zhang W. Depression Disorder Classification of fMRI Data Using Sparse Low-Rank Functional Brain Network and Graph-Based Features. *Comput Math Methods Med*. 2017;2017:3609821. doi:10.1155/2017/3609821
290. Whitfield-Gabrieli S, Ghosh SS, Nieto-Castanon A, et al. Brain connectomics predict response to treatment in social anxiety disorder. *Mol Psychiatry*. May 2016;21(5):680-5. doi:10.1038/mp.2015.109
291. Williams LM, Korgaonkar MS, Song YC, et al. Amygdala Reactivity to Emotional Faces in the Prediction of General and Medication-Specific Responses to Antidepressant Treatment in the Randomized iSPOT-D Trial. *Neuropsychopharmacology*. Sep 2015;40(10):2398-408. doi:10.1038/npp.2015.89
292. Wu Z, Wang C, Ma Z, et al. Abnormal functional connectivity of habenula in untreated patients with first-episode major depressive disorder. *Psychiatry Res*. Jan 31 2020;285:112837. doi:10.1016/j.psychres.2020.112837
293. Wu P, Zhang A, Sun N, et al. Cortical Thickness Predicts Response Following 2 Weeks of SSRI Regimen in First-Episode, Drug-Naive Major Depressive Disorder: An MRI Study. *Front Psychiatry*. 2021;12:751756. doi:10.3389/fpsyt.2021.751756
294. Wu CT, Huang HC, Huang S, et al. Resting-State EEG Signal for Major Depressive Disorder Detection: A Systematic Validation on a Large and Diverse Dataset. *Biosensors (Basel)*. Dec 6 2021;11(12)doi:10.3390/bios11120499
295. Wu MJ, Mwangi B, Bauer IE, et al. Identification and individualized prediction of clinical phenotypes in bipolar disorders using neurocognitive data, neuroimaging scans and machine learning. *Neuroimage*. Jan 15 2017;145(Pt B):254-264. doi:10.1016/j.neuroimage.2016.02.016
296. Xi C, Liu Z, Zeng C, et al. The centrality of working memory networks in differentiating bipolar type I depression from unipolar depression: A task-fMRI study. *Can J Psychiatry*. Mar 4 2022:7067437221078646. doi:10.1177/07067437221078646
297. Xiao H, Yuan M, Li H, et al. Functional connectivity of the hippocampus in predicting early antidepressant efficacy in patients with major depressive disorder. *J Affect Disord*. Aug 1 2021;291:315-321. doi:10.1016/j.jad.2021.05.013
298. Xing M, Fitzgerald JM, Klumpp H. Classification of Social Anxiety Disorder With Support Vector Machine Analysis Using Neural Correlates of Social Signals of Threat. *Front Psychiatry*. 2020;11:144. doi:10.3389/fpsyt.2020.00144
299. Xue L, Pei C, Wang X, et al. Predicting Neuroimaging Biomarkers for Antidepressant Selection in Early Treatment of Depression. *J Magn Reson Imaging*. Aug 2021;54(2):551-559. doi:10.1002/jmri.27577
300. Yan B, Xu X, Liu M, et al. Quantitative Identification of Major Depression Based on Resting-State Dynamic Functional Connectivity: A Machine Learning Approach. *Front Neurosci*. 2020;14:191. doi:10.3389/fnins.2020.00191
301. Yan DD, Zhao LL, Song XW, Zang XH, Yang LC. Automated detection of clinical depression based on convolution neural network model. *Biomed Tech (Berl)*. Apr 26 2022;67(2):131-142. doi:10.1515/bmt-2021-0232

302. Yan M, Cui X, Liu F, et al. Abnormal Default-Mode Network Homogeneity in Melancholic and Nonmelancholic Major Depressive Disorder at Rest. *Neural Plast*. 2021;2021:6653309. doi:10.1155/2021/6653309
303. Yan C, Yang X, Yang R, et al. Treatment Response Prediction and Individualized Identification of Short-Term Abstinence Methamphetamine Dependence Using Brain Graph Metrics. *Front Psychiatry*. 2021;12:583950. doi:10.3389/fpsyt.2021.583950
304. Yan M, He Y, Cui X, et al. Disrupted Regional Homogeneity in Melancholic and Non-melancholic Major Depressive Disorder at Rest. *Front Psychiatry*. 2021;12:618805. doi:10.3389/fpsyt.2021.618805
305. Yang P, Zhao C, Yang Q, et al. Diagnosis of obsessive-compulsive disorder via spatial similarity-aware learning and fused deep polynomial network. *Med Image Anal*. Jan 2022;75:102244. doi:10.1016/j.media.2021.102244
306. Yang X, Hu X, Tang W, et al. Multivariate classification of drug-naive obsessive-compulsive disorder patients and healthy controls by applying an SVM to resting-state functional MRI data. *BMC Psychiatry*. Jul 5 2019;19(1):210. doi:10.1186/s12888-019-2184-6
307. Yang J, Zhang M, Ahn H, et al. Development and evaluation of a multimodal marker of major depressive disorder. *Hum Brain Mapp*. Nov 2018;39(11):4420-4439. doi:10.1002/hbm.24282
308. Yang J, Yin Y, Zhang Z, et al. Predictive brain networks for major depression in a semi-multimodal fusion hierarchical feature reduction framework. *Neurosci Lett*. Feb 5 2018;665:163-169. doi:10.1016/j.neulet.2017.12.009
309. Yang J, Pu W, Ouyang X, et al. Abnormal Connectivity Within Anterior Cortical Midline Structures in Bipolar Disorder: Evidence From Integrated MRI and Functional MRI. *Front Psychiatry*. 2019;10:788. doi:10.3389/fpsyt.2019.00788
310. Yang H, Li L, Peng H, et al. Alterations in regional homogeneity of resting-state brain activity in patients with major depressive disorder screening positive on the 32-item hypomania checklist (HCL-32). *J Affect Disord*. Oct 2016;203:69-76. doi:10.1016/j.jad.2016.05.004
311. Yang T, Frangou S, Lam RW, et al. Probing the clinical and brain structural boundaries of bipolar and major depressive disorder. *Transl Psychiatry*. Jan 14 2021;11(1):48. doi:10.1038/s41398-020-01169-7
312. Yeh YW, Ho PS, Kuo SC, et al. Disproportionate Reduction of Serotonin Transporter May Predict the Response and Adherence to Antidepressants in Patients with Major Depressive Disorder: A Positron Emission Tomography Study with 4-[18F]-ADAM. *Int J Neuropsychopharmacol*. Jan 7 2015;18(7):pyu120. doi:10.1093/ijnp/pyu120
313. Yoshida K, Shimizu Y, Yoshimoto J, et al. Prediction of clinical depression scores and detection of changes in whole-brain using resting-state functional MRI data with partial least squares regression. *PLoS One*. 2017;12(7):e0179638. doi:10.1371/journal.pone.0179638
314. Yu H, Li F, Wu T, et al. Functional brain abnormalities in major depressive disorder using the Hilbert-Huang transform. *Brain Imaging Behav*. Dec 2018;12(6):1556-1568. doi:10.1007/s11682-017-9816-6
315. Yun JY, Jang JH, Kim SN, Jung WH, Kwon JS. Neural Correlates of Response to Pharmacotherapy in Obsessive-Compulsive Disorder: Individualized Cortical Morphology-Based Structural Covariance. *Prog Neuropsychopharmacol Biol Psychiatry*. Dec 3 2015;63:126-33. doi:10.1016/j.pnpbp.2015.06.009

316. Zehong C, Chin-Teng L, Weiping D, Mu-Hong C, Cheng-Ta L, Tung-Ping S. Identifying Ketamine Responses in Treatment-Resistant Depression Using a Wearable Forehead EEG. *IEEE Trans Biomed Eng*. Jun 2019;66(6):1668-1679. doi:10.1109/TBME.2018.2877651
317. Zeng LL, Shen H, Liu L, et al. Identifying major depression using whole-brain functional connectivity: a multivariate pattern analysis. *Brain*. May 2012;135(Pt 5):1498-507. doi:10.1093/brain/aws059
318. Zhai T, Gu H, Yang Y. Cox Regression Based Modeling of Functional Connectivity and Treatment Outcome for Relapse Prediction and Disease Subtyping in Substance Use Disorder. *Front Neurosci*. 2021;15:768602. doi:10.3389/fnins.2021.768602
319. Zhang A, Wang X, Li J, et al. Resting-State fMRI in Predicting Response to Treatment With SSRIs in First-Episode, Drug-Naive Patients With Major Depressive Disorder. *Front Neurosci*. 2022;16:831278. doi:10.3389/fnins.2022.831278
320. Zhang B, Liu S, Liu X, et al. Discriminating subclinical depression from major depression using multi-scale brain functional features: A radiomics analysis. *J Affect Disord*. Jan 15 2022;297:542-552. doi:10.1016/j.jad.2021.10.122
321. Zhang Y, Kong Y, Liu X, et al. Desynchronized Functional Activities Between Brain White and Gray Matter in Major Depression Disorder. *J Magn Reson Imaging*. May 2021;53(5):1375-1386. doi:10.1002/jmri.27466
322. Zhang J, Richardson JD, Dunkley BT. Classifying post-traumatic stress disorder using the magnetoencephalographic connectome and machine learning. *Sci Rep*. Apr 3 2020;10(1):5937. doi:10.1038/s41598-020-62713-5
323. Zhang Q, Wu Q, Zhu H, et al. Multimodal MRI-Based Classification of Trauma Survivors with and without Post-Traumatic Stress Disorder. *Front Neurosci*. 2016;10:292. doi:10.3389/fnins.2016.00292
324. Zhao J, Huang J, Zhi D, et al. Functional network connectivity (FNC)-based generative adversarial network (GAN) and its applications in classification of mental disorders. *J Neurosci Methods*. Jul 15 2020;341:108756. doi:10.1016/j.jneumeth.2020.108756
325. Zhao L, Wang Y, Jia Y, et al. Microstructural Abnormalities of Basal Ganglia and Thalamus in Bipolar and Unipolar Disorders: A Diffusion Kurtosis and Perfusion Imaging Study. *Psychiatry Investig*. Jul 2017;14(4):471-482. doi:10.4306/pi.2017.14.4.471
326. Zhdanov A, Atluri S, Wong W, et al. Use of Machine Learning for Predicting Escitalopram Treatment Outcome From Electroencephalography Recordings in Adult Patients With Depression. *JAMA Netw Open*. Jan 3 2020;3(1):e1918377. doi:10.1001/jamanetworkopen.2019.18377
327. Zheng Y, Chen X, Li D, et al. Treatment-naive first episode depression classification based on high-order brain functional network. *J Affect Disord*. Sep 1 2019;256:33-41. doi:10.1016/j.jad.2019.05.067
328. Zhong X, Shi H, Ming Q, et al. Whole-brain resting-state functional connectivity identified major depressive disorder: A multivariate pattern analysis in two independent samples. *J Affect Disord*. Aug 15 2017;218:346-352. doi:10.1016/j.jad.2017.04.040
329. Zhou C, Cheng Y, Ping L, et al. Support Vector Machine Classification of Obsessive-Compulsive Disorder Based on Whole-Brain Volumetry and Diffusion Tensor Imaging. *Front Psychiatry*. 2018;9:524. doi:10.3389/fpsyt.2018.00524
330. Zhu Z, Lei D, Qin K, et al. Combining Deep Learning and Graph-Theoretic Brain Features to Detect Posttraumatic Stress Disorder at the Individual Level. *Diagnostics (Basel)*. Aug 5 2021;11(8)doi:10.3390/diagnostics11081416

331. Zhu X, Yuan F, Zhou G, et al. Cross-network interaction for diagnosis of major depressive disorder based on resting state functional connectivity. *Brain Imaging Behav*. Jun 2021;15(3):1279-1289. doi:10.1007/s11682-020-00326-2
332. Zhu H, Yuan M, Qiu C, et al. Multivariate classification of earthquake survivors with post-traumatic stress disorder based on large-scale brain networks. *Acta Psychiatr Scand*. Mar 2020;141(3):285-298. doi:10.1111/acps.13150
333. Zhu X, Du X, Kerich M, Lohoff FW, Momenan R. Random forest based classification of alcohol dependence patients and healthy controls using resting state MRI. *Neurosci Lett*. May 29 2018;676:27-33. doi:10.1016/j.neulet.2018.04.007
334. Zhu J, Cai H, Yuan Y, et al. Variance of the global signal as a pretreatment predictor of antidepressant treatment response in drug-naive major depressive disorder. *Brain Imaging Behav*. Dec 2018;12(6):1768-1774. doi:10.1007/s11682-018-9845-9
335. Zhu Y, Qi S, Zhang B, et al. Connectome-Based Biomarkers Predict Subclinical Depression and Identify Abnormal Brain Connections With the Lateral Habenula and Thalamus. *Front Psychiatry*. 2019;10:371. doi:10.3389/fpsyt.2019.00371
336. Zhutovsky P, Thomas RM, Olff M, et al. Individual prediction of psychotherapy outcome in posttraumatic stress disorder using neuroimaging data. *Transl Psychiatry*. Dec 2 2019;9(1):326. doi:10.1038/s41398-019-0663-7
